# Multi-Target In-Silico modeling strategies to discover novel angiotensin converting enzyme and neprilysin dual inhibitors

**DOI:** 10.1038/s41598-024-66230-7

**Published:** 2024-07-10

**Authors:** Sapan K. Shah, Dinesh R. Chaple, Vijay H. Masand, Rahul D. Jawarkar, Somdatta Chaudhari, A. Abiramasundari, Magdi E. A. Zaki, Sami A. Al-Hussain

**Affiliations:** 1 Department of Pharmaceutical Chemistry, Priyadarshini J. L. College of Pharmacy, Hingna Road, Nagpur, 440016 Maharashtra India; 2 Department of Chemistry, Vidya Bharati Mahavidyalaya, Amravati, 444602 Maharashtra India; 3 Department of Medicinal Chemistry and Drug Discovery, Dr. Rajendra Gode Institute of Pharmacy, University Mardi Road, Amravati, 444603 India; 4 Department of Pharmaceutical Chemistry, Modern College of Pharmacy, Nigdi, Pune, India; 5 Biobay, Ahmedabad, India; 6 Department of Chemistry, College of Science, Imam Mohammad Ibn Saud Islamic University, Riyadh, 11623 Saudi Arabia

**Keywords:** Cardiovascular diseases, Multi-target inhibitors, Machine learning, Heterocyclic Scaffold, Molecular Simulations, ADMET, Drug-likeness, Computational biology and bioinformatics, Drug discovery

## Abstract

Cardiovascular diseases, including heart failure, stroke, and hypertension, affect 608 million people worldwide and cause 32% of deaths. Combination therapy is required in 60% of patients, involving concurrent Renin–Angiotensin–Aldosterone-System (RAAS) and Neprilysin inhibition. This study introduces a novel multi-target *in-silico* modeling technique (mt-QSAR) to evaluate the inhibitory potential against Neprilysin and Angiotensin-converting enzymes. Using both linear (GA-LDA) and non-linear (RF) algorithms, mt-QSAR classification models were developed using 983 chemicals to predict inhibitory effects on Neprilysin and Angiotensin-converting enzymes. The Box-Jenkins method, feature selection method, and machine learning algorithms were employed to obtain the most predictive model with ~ 90% overall accuracy. Additionally, the study employed virtual screening of designed scaffolds (Chalcone and its analogues, 1,3-Thiazole, 1,3,4-Thiadiazole) applying developed mt-QSAR models and molecular docking. The identified virtual hits underwent successive filtration steps, incorporating assessments of drug-likeness, ADMET profiles, and synthetic accessibility tools. Finally, Molecular dynamic simulations were then used to identify and rank the most favourable compounds. The data acquired from this study may provide crucial direction for the identification of new multi-targeted cardiovascular inhibitors.

## Introduction

CVDs such as heart failure, stroke, and hypertension affect 607.6 million people worldwide and are responsible for > 30% of all deaths^1,2^. Minimization of high blood pressure to normal is preferred to reduce the risk of CVD^3^. To achieve the desired goal of hypertension, a single targeting agent failed, and combination therapy was required in more than 60% of patients^4,5^, evident from clinical trials [ALLHAT (60%)^6^; PROGRESS (58%)^7^, INVEST (70%)^8^, INCLUSIVE (70%)^9^, and SHIELD (74%)^10^]. Randomized controlled trials (RCTs) are recommended to include four primary classes of antihypertensive medications viz. ACE Inhibitors (ACEIs), Angiotensin Receptor Blocker (ARBs), Calcium Channel Blocker (CCBs), and Thiazides-Type Diuretics (TTDs)^11^. Experimental evidence suggests ACEIs and/or ARBs are crucial for preventing and managing CVD, with JNC8 guidelines suggesting preferred classes of drugs like ACEIs and Neprilysin Inhibitor^12–14^. ACE which catalyzes the conversion of angiotensin-I to angiotensin-II is a membrane-bound dipeptidyl carboxyl peptidase that has two important active sites viz. zinc-binding site and cationic binding site and occupies an important niche in the regulation of extracellular volume by the rennin angiotensin system^15,16^. Selective inhibition of C-domain ACE (cACE) has the advantage of reducing angiotensin II production, a potent vasoconstrictor implicated in hypertension while preserving N-domain ACE (nACE) activity involved in bradykinin degradation, leading to increased vasodilatory effects and lower blood pressure. Furthermore, selective cACE inhibition may result in fewer side effects compared to non-selective ACEIs^17^. Neprilysin (NEP), a membrane-bound zinc endopeptidase, is inhibited by the Renin–Angiotensin–Aldosterone-System (RAAS), reducing blood pressure, arterial stiffness, cardiac hypertrophy, and fibrosis^18^. Lowered cardiovascular risk and blood pressure can be achieved with both ACE inhibitors and ARBs, with the former having fewer adverse effects than the latter^19^. Clinical trials have shown that RAAS blockade through the use of ACEIs, ARBs, and mineralocorticoid receptor antagonists can significantly lower risks for heart failure hospitalization and cardiovascular mortality. Therefore, inhibition of the RAAS system is an important strategy for preventing and managing cardiovascular disease^13,14^. The combination of ACE and NEP inhibitors has been shown to decrease heart failure and mortality rates, indicating a variety of CVD management strategies^12,20–27^. However, due to the synergistically suppressed bradykinin breakdown, there is an increased risk of angioedema, thus care must be taken while using the above combination^28^. Multi-targeted drugs can provide a more comprehensive treatment approach that addresses the underlying complexity of these diseases^29^. Therefore, endeavors have been made for synthesizing effective unique molecules having inhibitory effects on multiple targets^30^. Fixed-dose drug combinations can cause complications due to complex PK/PD relationships, but designing a molecule with potent activity at different targets offers an alternative^31,32^. These highlight the necessity for a distinctive molecule that effectively inhibits the RAAS and NEP.

In silico techniques are crucial in drug discovery due to their multifaceted use for data collection, pre-processing, analysis, and inference. Molecular modelling and virtual experimentation foundational concepts have minimized wet-lab chemical experimentation^33,34^. Quantitative Structure Activity Relationship (QSAR) and molecular simulations are widely used in drug design for providing critical and superlative evidence for lead/drug optimization^35^. QSAR investigations replace expensive synthesis and bioassay with computational models to accelerate drug selection in the early stages of development^36^. High-throughput virtual screening can be accelerated in the drug development process by using molecular docking and dynamics approaches^37^.

Candoxatrilat and Ecadotril demonstrated successful results in promoting natriuresis and increasing urine ANP discharge. As previously stated, the continuous utilization of Candoxatril did not maintain the initial reduction in blood pressure, resulting in the termination of further studies^38,39^. Conversely, the disappointing clinical results of NEP inhibitors as monotherapy may be overcome by combination with RAAS blockade^40^. The findings of the PARADIGM-HF, PARAGON-HF, and OCTAVE studies indicate an increased reduction in heart failure hospitalization and mortality rate using a combination of RAAS blocker and NEP inhibitor^24–27^.

Recent advancements in the pharmaceutical industry, such as the development of a Losartan and metabolite co-drug with NEP inhibitors, demonstrate innovative approaches in drug discovery^41^. The discovery of orally active TD-0212 and Gyrophoric acid further emphasizes the necessity of dual AT1/NEP inhibition with potentially lower angioedema risk^42,43^. Omapatrilat and LCZ696 (Entresto®) are molecules discovered to target the renin–angiotensin–aldosterone system and neprilysin together^44–47^. Ilepatril, (AVE7688) was designed to have improved specificity and prolong the ACE inhibition^48^. The research is crucial in light of the rising CVD prevalence and the limitations of current treatments. By concurrently modulating multiple targets, the proposed agents have the potential to significantly improve treatment outcomes.

The objective of this study is to identify novel multi-target inhibitors for the RAAS and NEP enzymes, which can effectively manage hypertension and lower the risk of CVDs. The study provides a rational drug design approach, using computational tools like molecular simulations and virtual screening based on QSAR before the synthesis and pharmacological assessment of novel multi-targeted cardiovascular agents. Machine learning algorithms are employed to develop mt-QSAR models for the screening of dual inhibitors of the ACE and NEP enzymes. Applicability domain (AD) studies are conducted for both machine learning and regression-based QSAR models, ensuring a rigorous selection process based on inhibitory potential (pIC_50_). Molecular docking and dynamics investigations are utilized to enhance comprehension of the interactions between the target and the selected screening compounds. Furthermore, ADMET screening, processed by QSAR-based models, enhances the evaluation of compound suitability.

## Material and methods

### Development of mt-QSAR models for two endpoints: ACE and NEP enzyme inhibition

#### Dataset collection, curation, and descriptor calculations

The molecular structural and biological data for 983 compounds which includes ACE (474) and NEP (509) with inhibitory activity against ACE and NEP enzymes of *Rattus norvegicus* (Supporting file S1) were extracted from the ChEMBL database (https://www.ebi.ac.uk/chembl). The selected compounds dataset was subjected to biological and chemical curation^49^ by removing the molecules that lack information such as SMILES, units of activity, and duplicates. Structures were standardised, neutralised, and cleaned and salts were removed to get a dataset of 715 compounds which makes ACE (357) and NEP (358) (Supporting file S2) followed by 3D optimization using MMFF94 force-field by OpenBabel^50,51^. we have calculated a variety of descriptors, namely, Py-Descriptor (Constitutional, geometric, circular fingerprint, quantum chemical and topological, no. of descriptors > 16,250), Alvadesc-Descriptors (Constitutional, topological, connectivity indices, 2D matrix-based, ETA indices, atom type E-state, functional group count, 2D atom pair, atom-centered fragments, molecular properties and drug like indices, no. of descriptors ≥ 3117), PaDEL-Descriptor 2.20 (for extended topochemical atom indices, no. of descriptors = 242) through freely available web server OCHEM (https://ochem.eu/home/show.do) (Supporting file S3).

Alvadesc descriptors calculated based on chemical structure do not show any discrimination when a specific molecule is assayed under more than one experimental condition. Box-Jenkins operators provide a solution to the above problem, as they calculate successive average values of a defined property at different time intervals. In Box–Jenkins operators are used to calculate modified descriptors which are capable of discriminating the influence on the chemical structure when a specific molecule is assayed under more than one experimental condition (Supporting file S4). In Box–Jenkins-based mt-QSAR modeling, the arithmetic average of any molecular descriptors for a specific experimental condition is calculated as follows-

1 $$avg {({D}_{i})}_{{C}_{j}}={\sum }_{i=1}^{n({C}_{i})}{\text{D}}_{i}$$

where the *avg*(D_i_)c_j_ is thus the arithmetic mean of the descriptors (D_i_) for a specific experimental condition (c_j_). After generating the avg(D_i_)c_j_ values, the final modified descriptors (∆(D_i_) c_j_) are subsequently generated using the following formula-

2 $$\Delta {({D}_{i})}_{{C}_{j}}={D}_{i}-avg {({D}_{i})}_{{C}_{j}}$$

where ∆(D_i_)c_j_ is a deviation descriptor that measures to what extent a chemical structurally deviates from a set of compounds assigned as active and tested against the same experimental condition.

#### Development of mt-QSAR model

**Figure 1:**
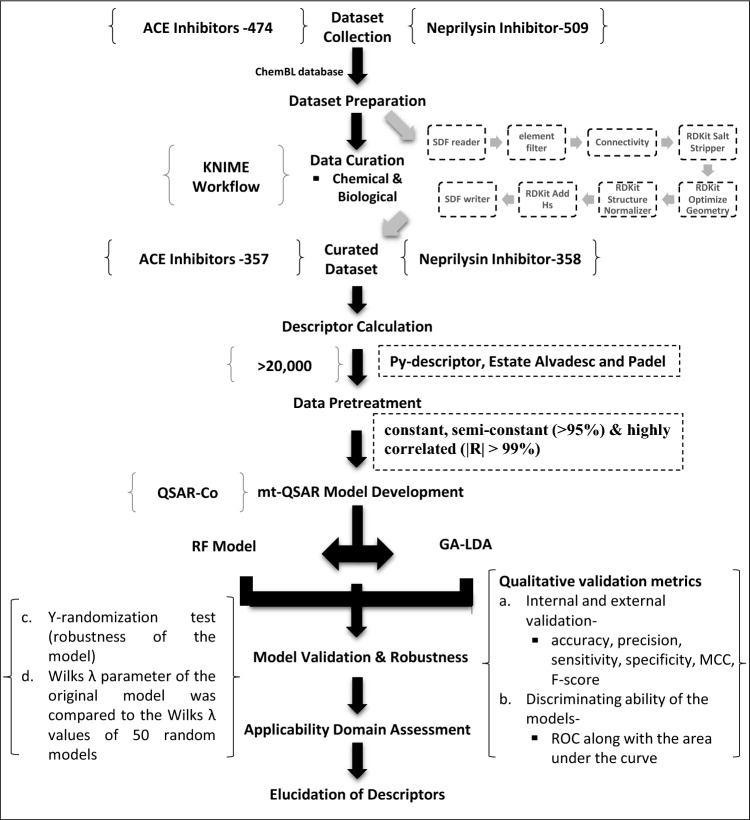
Sequential steps involved in the development of mt-QSAR models for dual endpoint detection (ACE and NEP enzyme inhibition).

The QSAR-Co program develops mt-QSAR models employing GA-LDA and RF algorithms simultaneously predict two endpoints, angiotensin-converting-enzyme, and neprilysin enzyme inhibition, under a variety of experimental and theoretical conditions using a single QSAR model Eq. ^52^. Three experimental conditions (Tn-ACE/NEP, St-IC_50_/KI, At-B/F) were considered for developing respective mt-QSAR models (denoted as ‘C’). The experimental condition Cj, a combination of conditions, is represented as an ecosystem with Tn, At, and St. Data points are tagged for target inhibition (TN) under Cj, with TNi(Cj) representing active or inactive classes. In the class assignments, compounds with IC_50_/Ki values ≤ 600 nM were classified as active, while the other data samples were considered inactive. The cutoff value selected in the sub-micromolar range confirms the meticulous search for potent hits^53,54^. The selected cut-off upholds the number of molecules annotated as active as high as possible and magnifies the chemical diversity, necessary to rationally design new molecules. This facilitates a way to have a balance between the number of molecules assigned as active and those labeled as inactive. The dataset in this work was randomly split into a test set (30%) and a training set (70%) using the random approach, to create an mt-QSAR model for dual endpoint ACE and NEP inhibition prediction. Two machine learning approaches, Random Forest (RF)^55^ and Linear Discriminant Analysis (LDA)^56,57^, were used to construct the final mt-models in the QSAR-Co programme. The mt-QSAR models were constructed using the QSAR-Co software's default parameter settings. The sequential steps involved in the development of mt-QSAR models are depicted in Fig. 1**.**

#### Validation of mt-QSAR model

Based on the qualitative metrics for validation calculated for the training set, the best LDA and RF models were assessed and selected. The selected models were then externally validated using the test set. For internal and external validation, respectively, qualitative validation measures including accuracy, precision, sensitivity, specificity, MCC, and F-score were computed for the training and test sets^58^. In order to ascertain the developed LDA and RF models' ability to discriminate, the receiver operating characteristics (ROC) curve and area under the curve (AUC) were examined^58^. Using the Y-randomization test, which involves randomly generating 50 LDA models after the dependent variable (response class) of the training set is scrambled 50 times, the robustness of the LDA model was additionally evaluated^59^. To rule out the possibility that the original LDA model was created by chance, the Wilks λ parameter of the model was also compared to the Wilks λ values of 50 random models. Ultimately, the standardization technique was used to identify the applicability domain for both LDA and RF models^60^.

### Combinatorial library designing of novel chalcone derivatives, thiazole derivatives, and thiadiazole derivatives

**Figure 2:**
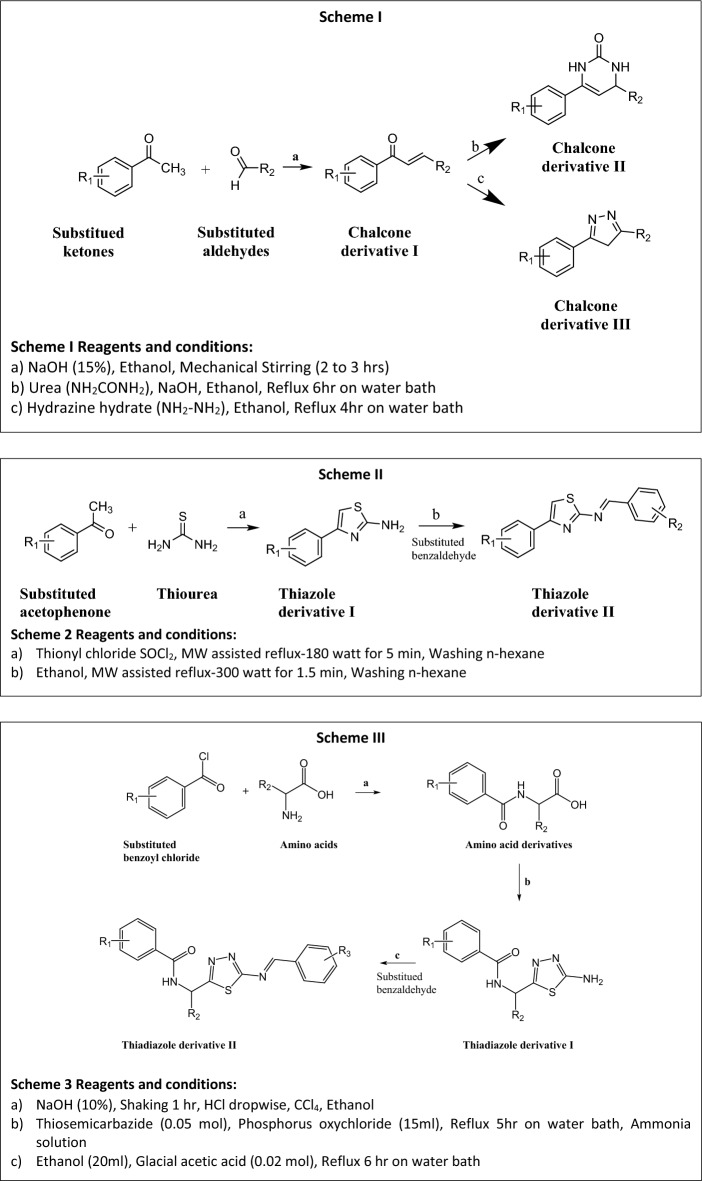
Scheme utilized for designing of novel chalcone derivatives, thiazole derivatives, and thiadiazole derivatives.

Three heterocyclic scaffolds viz. chalcones and its derivatives (235 compounds), 1, 3-Thiazole (24 compounds), and 1,3,4-Thiadiazole (107 compounds) were designed using DataWarrior software version 0.5.05.00** (**Fig. 2**)**. Considering the structural requirements indicated by the developed QSAR model, the chemical moieties were designed and synthesized (Supporting file S5).

The chemical similarity analysis with the compounds database in CAS reveals that designed chemical structures are novel and have not been studied previously for RAAS and NEP inhibition. All the designed compounds were sketched and were 3D optimized using MMFF94 force-field by OpenBabel, and descriptors (PyDescriptor, Alvadesc, Estate, and Padel) were calculated. The optimized designed compounds' applicability domain was predicted using the most robust and statistically acceptable QSAR models.

### Screening of designed derivatives using mt-QSAR models

In addition to model development, the QSAR-Co tool also allows the screening of large datasets. Using this screening facility, the mt-QSAR-LDA and RF models were used to screen external validation sets of designed chalcone, 1,3-thiazole, 1,3,4-thiadiazole, and quinazoline derivatives. This helps in the classification or determination of two classes of active dual inhibitors viz. i) ACE and NEP enzymes.

### Constraint-based molecular docking study of selected designed derivatives

In this analysis, we have applied the molecular docking studies to investigate the binding pattern of selected screened designed molecules with the cACE (PDB ID: 1O86) and NEP (PDB ID: 5JMY) obtained from the protein data bank (https://www.rcsb.org).

#### A platform for molecular docking

The computational docking assessment of selected compounds with ACE as a target was executed via MOE 2019 software (Chemical Computing Group, Montreal, Canada), software.

#### Protein selection

**Figure 3:**
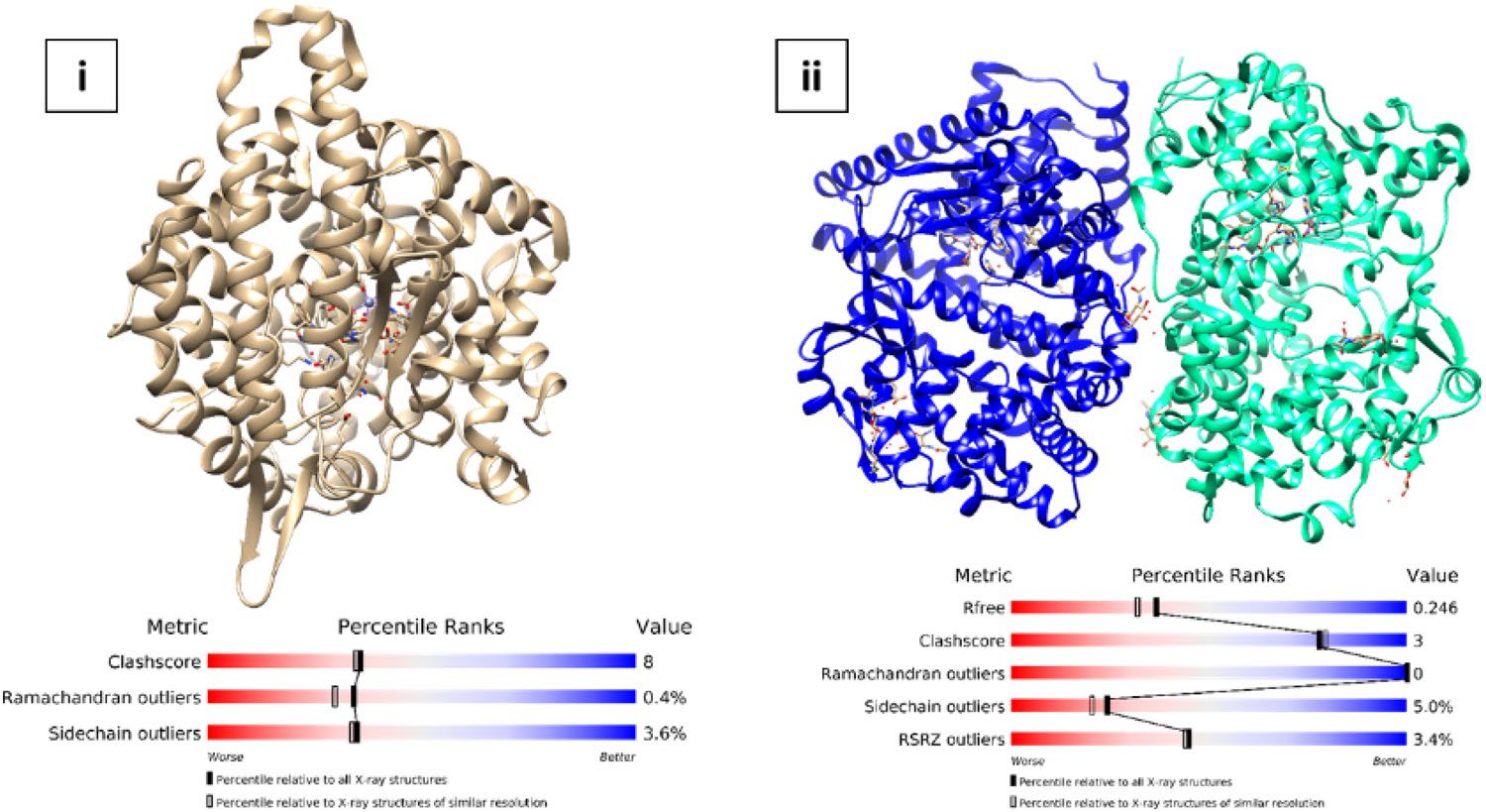
Crystal structures of the selected target for molecular simulations study (**i**) cACE (PDB ID: 1O86) (**ii**) (**ii**) NEP (PDB ID: 5JMY).

The X-ray crystal structures of the target proteins selected for molecular docking are given in (Fig. 3). i) cACE (PDB ID: 1O86, crystal structure of human ACE in complex with Lisinopril, Resolution: 2.00 Å) and ii) NEP (PDB ID: 5JMY, NEP complexed with LBQ657, Resolution: 2.00 Å).

#### Molecular docking

##### Protein preparation

**Figure 4:**
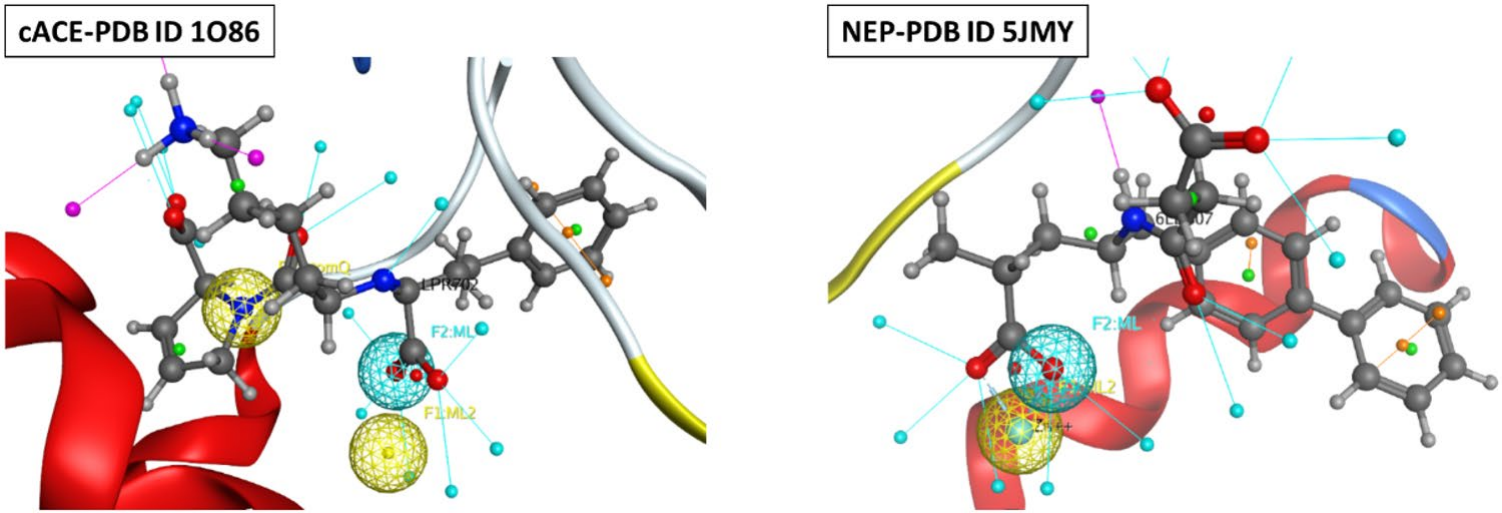
An illustration of the metal chelation and positional constraints created and implemented for docking chemical compounds against cACE (PDB ID: 1o86) and NEP (PDB ID: 5JMY).

The selected models were prepared by deleting co-crystallized water molecules, unwanted chains, and nonstandard residues. The free target protein was then subjected to the QuickPrep procedure of MOE including corrections for missing atoms, alternate geometries, or other crystallographic artifacts, removing water molecules farther than 4.5 Å from any receptor or ligand atom, and 3D protonation. In the case of cACE and NEP docking, pharmacophore constraints were generated using the pharmacophore query editor containing metal chelation constraint and one positional constrain amide/amine Nitrogen with 1 Å constraint sphere (Fig. 4).

##### Ligand preparations

All chemical structures are sketched in MarvinSketch^61^ and converted into SDF format using OpenBabel considering 2D geometry optimization^62^. The ligands dataset was further subjected to 3D optimization using MMFF94 force field using the Konstanz Information Miner (KNIME) workflow (https://www.knime.org/)^50^.

##### Docking procedure

The selected metal chelation constraint and one positional constraint amide/amine nitrogen constraints were implicated in the docking using the pharmacophore placement method at the site centered on co-crystallized ligand atoms and the top 1000 poses ranked by the London dG scoring function. From these poses, the best 30 poses were ranked and then minimized using MMFF94 × forcefield within a rigid receptor. The resulting poses were then refined and scored using the Generalized-Born Volume Integral/Weighted Surface area (GBVI/WSA) dG scoring function which estimates the binding free energy for an obtained pose of the ligand. The final results were analyzed, and visualized based on docking scores and pose using Discovery Studio 2020 Client^63^, and PyMol software^64^ considering bound ligand as standard. Visualization of protein–ligand interaction reflects, the number of interactions and active residues responsible for the significant binding at the active site target enzyme.

### Molecular dynamics study and MM-GBSA calculations

Desmond version 2020.1 with OPLS3e force field from Schrodinger was used to study the dynamic behavior of selected molecule complexes in the presence of explicit water molecules^65^. The System Builder module was used for system preparation using the SPC module for solvation and volume occupancy in an orthorhombic box with periodic boundary conditions. The solvated system was neutralized by the addition of appropriate anion (Cl^-^) and cation (Na^+^) with a salt concentration of 0.15 mol. The Nose–Hoover chain coupling approach was employed to build up the NPT ensemble with a temperature of 300 K, leisure time of 1.0 ps, and pressure of 1 bar, which was once as soon as maintained in all simulations using a 2 fs time step. The barostat approach with the Martyna–Tuckerman–Klein chain coupling scheme was originally utilized for pressure control with a leisure time of 2 ps. The particle mesh Ewald technique was used to calculate long-range electrostatic interactions with a radius of 9 for Coulomb interactions. The non-bonded forces were estimated using the RESPA integrator. The root mean square deviation (RMSD), root mean square fluctuation (RMSF), radius of gyration (Rg), and protein–ligand interactions were assessed to check the stability of the complex in MD simulations^66^.

Moreover, Prime Molecular Mechanics with Generalised Born Surface Area (MM-GBSA) Schrodinger, NY, 2019 (Release, 2017) was used to ascertain the relative binding affinity of the ligands towards selected target proteins. The solvent model and force field for the MM-GBSA^67^ computations were OPLS3^65^ and VSGB^68^. The binding free energy in MM/GBSA was calculated using the equation that follows^69,70^.

$$\Delta {G}_{bind}={G}_{complex}-{G}_{protein}-{G}_{ligand}$$

$$=\Delta H+{\Delta G}_{solvation}-T\Delta S$$

$$=\Delta {E}_{MM}+{\Delta G}_{GB}+\Delta {G}_{SA}-T\Delta S$$

### Drug-likeness, PAINS assay, and In silico toxicology study of selected design compounds

In this study, we conducted a comprehensive evaluation of selected design compounds through a multi-step process. First, compounds were filtered based on drug-likeness using Lipinski, Ghose, Veber, Egan, and Muegge rules via the SwissADME webserver (http://www.swissadme.ch). Subsequently, a PAINS assay was performed to identify potential interference compounds. In silico toxicology, predictions were then carried out, including LD_50_ estimation using Protox-II webserver (https://tox-new.charite.de) and calculation of mutagenicity, carcinogenicity, hepatotoxicity, cardiotoxicity, etc. using VEGA-QSAR software (https://www.vegahub.eu/portfolio-item/vega-qsar)^71^. The integrated results informed the identification of promising compounds with favorable drug-like properties and reduced risk of adverse effects, guiding further drug discovery efforts.

### Ethics approval

All authors certify that they have no affiliations with or involvement in any organization or entity with any financial interest or non-financial interest in the subject matter or materials discussed in this manuscript.

## Results and discussion

### Development of multi-target QSAR models to screen novel-designed chalcone, 1,3-thiazole, and 1,3,4-thiadiazole derivatives as dual inhibitors of ACE and NEP enzymes

The developed models have significant discriminating power, as demonstrated by the optimum values obtained for statistical parameters including accuracy, precision, sensitivity, specificity, F-measure, and Mathew's Correlation Coefficient (MCC). All of the models demonstrated similar results for each compound in the test set. Thus, it can be concluded that the developed mt-QSAR models (LDA and RF) are properly capable of determining two endpoints for dual inhibition of ACE and NEP of newly designed compounds.

#### Linear discriminant analysis (LDA) based mt-QSAR model development

The QSAR-Co tool's multi-target modeling leverages the Box-Jenkins method. This method modifies molecular descriptors for each dataset compound, incorporating diverse conditions to enhance the predictive power of the model. The mt-QSAR model was constructed using a dataset consisting of a sub-training set (n = 501) and a test set (n = 214). The best-fit mt-QSAR-LDA model was chosen from all models with the least Wilks (λ)_train_ and highest MCC_train_, as shown in in Table 1 and accompanied by statistical parameters.

**Table 1:** The best-fit mt-QSAR-LDA model (Standard coefficients and fitness scores) for dual inhibition of ACE and NEP enzymes.

Coefficient	Descriptors	Coefficient	Descriptors	Coefficient	Descriptors
65.24	SpMAD_X_tn	− 2.822	O_lipo_3Ac_at	90.58	VE2sign_Dz(Z)_at
− 47.02	JGI2_tn	27.57	JGI1_at	0.109	ringC_MSA4_at
− 0.572	F04[O-S]_at	0.758	MaxsssCH_st	1.597	C_S_6Bc_st
− 0.297	fdonringC6B_at	0.379	fdonringC8B_at	0.199	VE3sign_B(v)_at
− 54.61	X5Av_st	8.26	acc_N_5Ac_tn	− 63.27	VE2sign_Dz(i)_at
2.911	ringN_C_8Ac_at	0.06	N_ringC_4A_st	− 0.293	fringNringC4B_st
0.728	O_C_5Ac_at	− 18.68	Eta_D_epsiA_tn	− 0.224	VE3sign_B(e)_st
1.543	MaxdssC_st	− 0.294	C1SP3_at	− 0.416	fringCC4B_at
137.23	JGI8_tn	0.577	minus_ringN_1B_tn	0.29	com_donminus_4A_tn
− 0.208	don_HASA3_tn	0.28	fdonacc8A_tn	0.208	com_sp2O_4A_st
− 0.159	VE3sign_B(p)_tn	0.445	fNdon3B_st	1.219	faccS4B_tn
0.432	fdonO3B_st	− 0.22	com_Cplus_4A_st	− 0.348	C_O_1B_tn
0.098	don_MSA2_tn	0.647	fringCdon4B_tn	0.051	fNC7B_at
				− 0.248	fringCO4B_tn
Intercept					0.677
Fitness score(GA-LDA; Using a training set only)					0.827
Wilks λ_(train)_					0.479
Wilks λ_(test)_					0.488

Table 1 shows standardized coefficient values, indicating selected descriptor's contribution to inhibitory activity. ‘JGI8_tn’ has maximum (positive) contribution (coefficient value = 137.2343) and ‘VE2sign_Dz(i)_at’ has the maximum (negative) contribution (coefficient value = − 63.265) towards enzymes inhibition. A succinct explanation of the significance, source, and contribution of each descriptor used for the final LDA model, as shown in Table 2.

**Table 2:** Symbols and definitions for the descriptors selected in the mt-QSAR (LDA) model for dual inhibition of ACE and NEP enzymes.

Descriptor (type)	Brief description	Descriptor type
SpMAD_X_tn	Spectral mean absolute deviation from chi matrix	2D matrix-based descriptors
JGI2_tn	Mean topological charge index of order 2	2D autocorrelations (Topological charge)
F04[O-S]_at	Frequency of O – S at topological distance 4	Pharmacophore descriptors
fdonringC6B_at	Frequency of occurrence of ring carbon atoms exactly at 6 bonds from donor atoms	Circular fingerprint
X5Av_st	Average valence connectivity index of order 5	Connectivity indices
ringN_C_8Ac_at	Sum of partial charges of carbon atoms within 8 A.U. from ring N atoms	Quantum chemical
O_C_5Ac_at	Sum of partial charges of carbon atoms within 5 A.U. from oxygen atoms	Quantum chemical
MaxdssC_st	Maximum atom-type E-State: = C <	Atom-type electro-topological state
JGI8_tn	Mean topological charge index of order 8	2D autocorrelations (Topological charge)
don_HASA3_tn	Solvent accessible surface area of donor atoms having partial charge in the range 0.0000 to -0.0999	Geometric
VE3sign_B(p)_tn	Logarithmic coefficient sum of the last eigenvector from the Burden matrix weighted by polarizability	2D matrix-based descriptors
fdonO3B_st	Frequency of occurrence of oxygen atom exactly at three bonds from donor atoms	Circular fingerprint
don_MSA2_tn	Molecular surface area of donor atoms with partial charge in the range -0.0999 to 0	Geometric
O_lipo_3Ac_at	Sum of partial charges of lipophilic atoms within 3 A.U. from oxygen atoms	Quantum chemical
JGI1_at	mean topological charge index of order 1	2D autocorrelations (Topological charge)
MaxsssCH_st	Maximum atom-type E-State: > CH-	Atom-type electrotopological state
fdonringC8B_at	Frequency of occurrence of ring carbon atoms exactly at eight bonds from donor atoms	Circular fingerprint
acc_N_5Ac_tn	Sum of partial charges of N atoms within 5 A.U. from acceptor atoms	Quantum chemical
N_ringC_4A_st	Presence of ring C atom within a distance of 4 A.U. from Nitrogen atoms	Topological
Eta_D_epsiA_tn	Eta measure of unsaturation and electronegative atom count	ETA indices
C1SP3_at	Singly bound carbon bound to one other carbon	PaDEL carbon type
minus_ringN_1B_tn	Number of negatively charged atoms from ring Nitrogen atoms within 1 bond i.e. directly attached to it	Topological
fdonacc8A_tn	Frequency of occurrence of acceptor atoms exactly at 8 Å from donor atoms	Circular fingerprint
fNdon3B_st	Frequency of occurrence of donor atoms exactly at three bonds from nitrogen atoms	Circular fingerprint
com_Cplus_4A_st	Number of positively charged carbon present within 4 Å from the center of mass (com),	Circular fingerprint
fringCdon4B_tn	Frequency of occurrence of donor atoms exactly at four bonds from ring carbon atoms	Circular fingerprint
VE2sign_Dz(Z)_at	Average coefficient of the last eigenvector from the Barysz matrix weighted by atomic number	2D matrix-based descriptors
ringC_MSA4_at	Molecular surface area of the ring carbon atom having partial charge in the range of 0.1000–0.1999	Geometric
C_S_6Bc_st	Sum of partial charges of carbon atoms present within six bonds from the sulfur atom	Quantum chemical
VE3sign_B(v)_at	Logarithmic coefficient sum of the last eigenvector from the Burden matrix weighted by Van der Waals volume	2D matrix-based descriptors
VE2sign_Dz(i)_at	Average coefficient of the last eigenvector from the Barysz matrix weighted by ionization potential	2D matrix-based descriptors
fringNringC4B_st	Frequency of occurrence of ring carbon atoms exactly at four bonds from ring Nitrogen atoms	Circular fingerprint
VE3sign_B(e)_st	Logarithmic coefficient sum of the last eigenvector from Burden matrix weighted by Sanderson electronegativity	
fringCC4B_at	Frequency of occurrence of carbon atoms exactly at four bonds from ring carbon atoms	Circular fingerprint
com_donminus_4A_tn	Number of negatively charged donor atoms present within 9 Å from the center of mass	Circular fingerprint
com_sp2O_4A_st	Number of sp2 hybridized oxygen atoms present within 4 Å from the center of mass	Circular fingerprint
faccS4B_tn	Frequency of occurrence of sulfur atoms exactly at four bonds from acceptor groups	Circular fingerprint
C_O_1B_tn	Presence of carbon atoms at a distance of 1 bond i.e. attached to oxygen atoms	Topological
fNC7B_at	Frequency of occurrence of carbon atoms exactly at seven bonds from nitrogen atoms	Circular fingerprint
fringCO4B_tn	Frequency of occurrence of oxygen atoms exactly at four bonds from ring carbon atoms	Circular fingerprint

The mt-QSAR-LDA model meets the requirements for robustness, quality of fit, and statistical significance. The Wilks λ statistic, with a value of 0.479, indicates the model's adequate discriminatory power. Table 3 presents the classification results for both the sub-training and test sets, providing an overview of the overall performance of the mt-QSAR-LDA model.

**Table 3:** Overall statistical performance of the final mt-QSAR (LDA and RF) models for dual inhibition of ACE and NEP enzymes.

Classification models evaluation parameters	LDA model		RF model	
	Training set	Test set	Training set	Test set
Total No. of compounds:	501	214	572	143
True positive	335	150	401.0	103.0
False positive	24	13	7.0	10.0
Sensitivity (%)	94.37	93.17	98.77	93.64
True negative	122	40	159.0	23.0
False negative	20	11	5.0	7.0
Specificity (%)	83.56	75.47	95.78	69.7
Accuracy (%)	91.22	88.79	97.90	88.11
Precision (%)	93.31	92.02	98.28	91.15
F-measure	0.9384	0.9259	0.9853	0.9238
MCC	0.7858	0.6954	0.9489	0.6554

Table 3 demonstrates the model's strong discrimination ability. Accuracy reached 91.22% and 88.79% for the sub-training and test sets, respectively. Moreover, it accurately classified 94.37% of active samples and 83.56% of inactive ones in the sub-training set, while achieving similar performance in the test set with 93.17% accuracy for active samples and 75.47% for inactive ones. These results support the high degree of efficiency of the mt-QSAR-LDA model to distinguish between active and inactive inhibitors. MCC values (0.7858 for sub-training, 0.6954 for test) further confirm the model's statistical robustness^72^.

**Figure 5:**
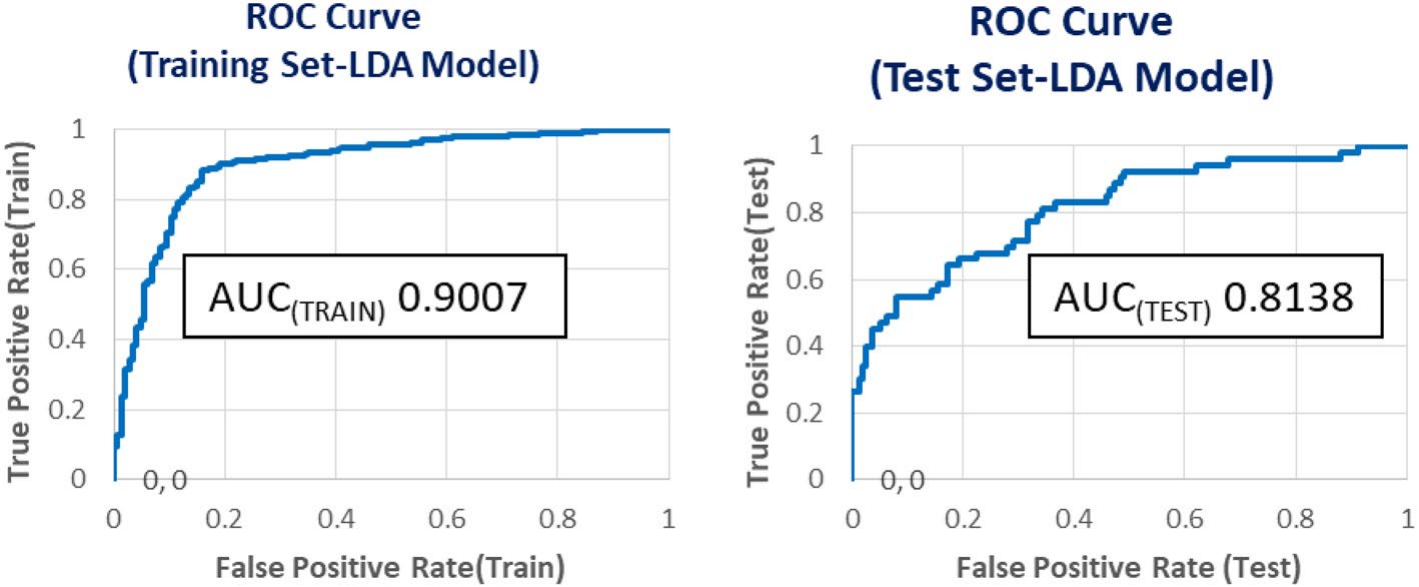
ROC (using tenfold cross-validation) plots for the best LDA model for dual inhibition of ACE and NEP enzymes.

Figure 5 shows the receiver operating characteristic curve (ROC) plot for the training and test set. The area under the ROC curve (AUROC) values of 0.9007 and 0.8138 were obtained, indicating good statistical significance of the mt-QSAR model. The higher AUROC for the training set is expected, as the model is built on this data. However, a value of 0.8138 on the test set suggests good generalizability to unseen data.

**Figure 6:**
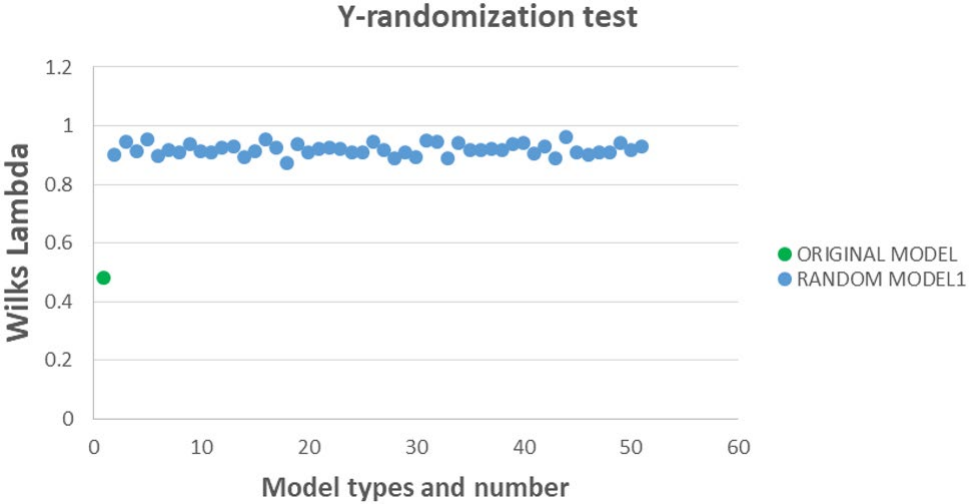
Y-randomization test results for the developed LDA model for dual inhibition of ACE and NEP enzymes.

The Y-randomization test^59^ indicates that the mt-QSAR model is not created by chance, as shown in Fig. 6, with Wilk's lambda values (50 model average λ_random_ = 0.9201) significantly higher than the original value (λ_train_ = 0.4791). The QSAR-Co software's standardization approach^60^ determined the applicability domain, revealing 6 out of 501 training data points and 5 out of 214 test data points as possible outliers and outside the applicability domain.

#### Non-linear mt-QSAR (Random Forest) model development for dual inhibition of ACE and NEP enzymes

**Figure 7:**
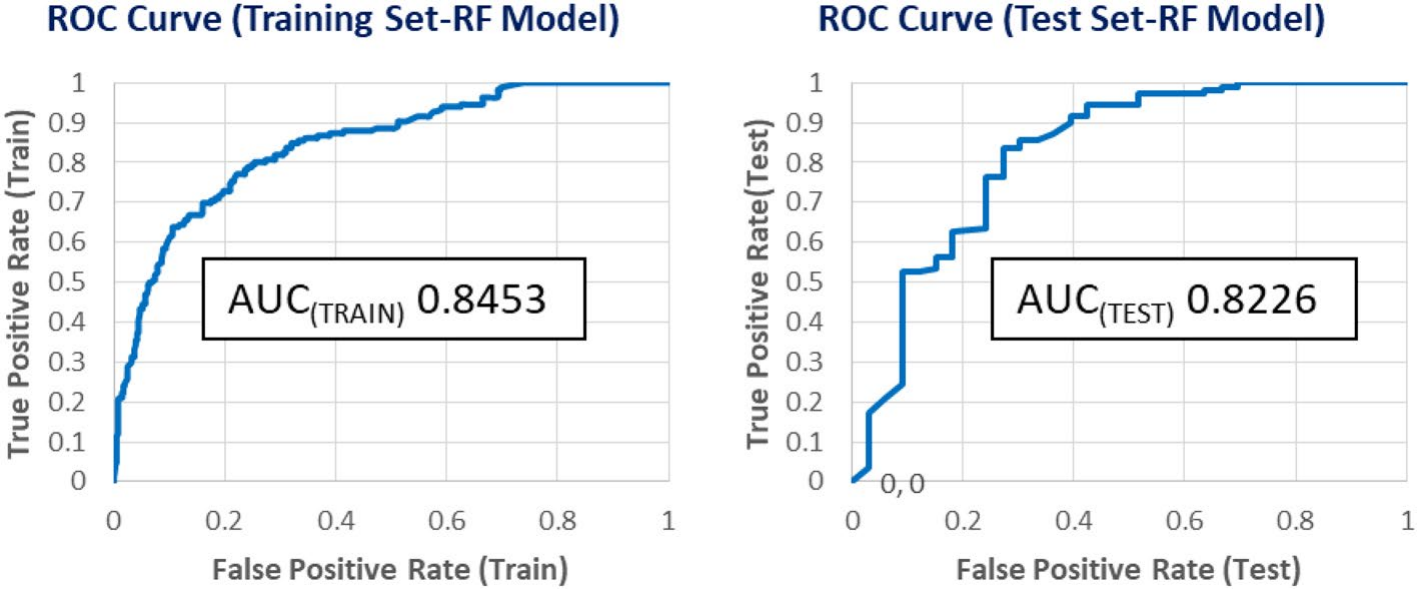
ROC (using tenfold cross-validation) plots for the best RF model.

The Random Forest (RF) technique was used to construct a non-linear classification-based mt-QSAR model using training and test sets, developed using QSAR-Co software and Weka version 3.9.3 library^73^. The RF model of QSAR-Co, with its default parameters (tenfold cross-validation procedure), demonstrated superior overall statistical prediction quality compared to the LDA model, as detailed in Table 3. The RF model outperforms the LDA model in predicting inactive and active compounds due to its superior specificity, precision, and accuracy values. Accordingly, employing both models would be always beneficial to perform consensus predictions for queries or newly designed compounds. Further, Fig. 7 shows the plots of the corresponding ROC curves for the RF model, and the AUC values for both the training set (= 0.8453) and the test set (= 0.8225) show that the model has significant discriminatory power.

Non-linear models with all computed descriptors often produce better predictive models than linear models with a subset of descriptors, but their interpretability is inferior. RF is a method for group categorization that averages predicted outcomes from several different decision trees to produce its predictions. The great precision and superiority of RF have drawn a lot of interest recently^74–77^. RF has multiple advantages, one of which is that it is less vulnerable to constructing overfitted models. Consequently, RF can be favoured over several other non-linear machine learning techniques to generate highly accurate mt-QSAR models^78,79^.

### Screening of designed derivatives using validated developed mt-QSAR models

**Figure 8:**
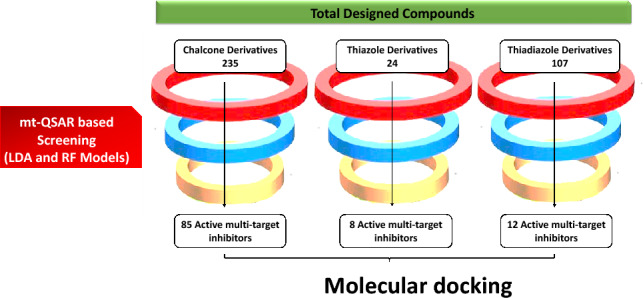
Screening of designed compounds using developed mt-QSAR models (LDA and RF). Compounds found active through both models are only selected for molecular docking study.

Using the facility available to screen large designed datasets in QSAR-Co software, the mt-QSAR models were used to screen externally designed derivatives set of four heterocyclic scaffolds viz. chalcones and its derivatives (235 compounds), 1, 3- Thiazole (24 compounds) and 1,3,4-Thiadiazole (107 compounds). Correspondingly, both LDA and RF models are faster in screening large-size databases with an accuracy of equivalent to 90% and an MCC value greater than 0.5. The selection of ligands is based on two primary criteria. Firstly, the ligand must fall within the applicability domain of our LDA models. Secondly, they are required to show a positive score for activity against both ACE and NEP under any given set of experimental conditions, as indicated by the results from our developed LDA and RF models for each of the designed heterocyclic derivatives. Details of this screened dataset and calculated descriptors, as well as the results of the predictions, are provided in Supporting file S6. Out of the 235 designed chalcone derivatives, 85 compounds meet all the criteria. Likewise, out of the 24 designed thiazoles, 8 meet the criteria; out of the 107 designed thiadiazoles, 12 compounds exhibit positive results. Thus, all designed compounds are screened using developed models, and only those molecules that follow the applicability domain and show positive prediction are processed further for molecular docking study **(**Fig. 8**).** Altogether, these diverse statistics demonstrate the high internal quality as well as the predictive power of the derived mt-QSAR models.

### Molecular docking studies of selected screened designed derivatives

In an effort to identify new starting point leads for novel multi-target inhibitors of ACE (C-domain selective) and NEP enzymes, molecular docking simulations were performed for the screened active hit suggested by mt-QSAR models from a combinatorial library of designed derivatives. There are a total of 85 chalcone derivatives, 8 thiazoles, and 12 thiadiazoles derivatives selected for molecular docking which passes the AD criteria of developed mt-QSAR models.

#### Validation of docking protocol

**Figure 9:**
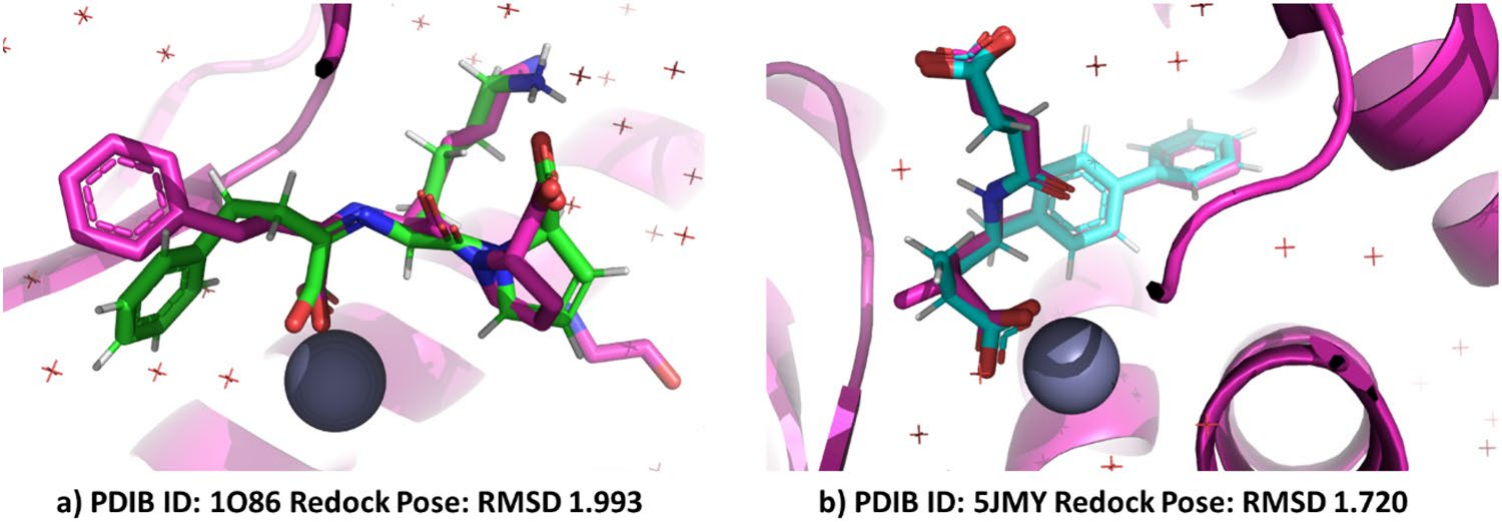
Validation of docking protocol by re-docking the native ligands (Lisinopril and LBQ657) at active binding site and interacting amino acid residues (Magenta color original poses, green color redocked pose of lisinopril and cyan color redocked pose of LBQ657).

The docking procedure was validated by re-docking the natural ligands (lisinopril and LBQ657 compounds) from crystal structures (PDB ID: 1O86 and 5JMY) into its binding pocket before virtual screening of chosen compounds. The large size and shape of the binding site pose challenges for molecular docking on targets. In cACE and NEP molecular docking calculations, constraints are applied to obtain reliable orientations of ligands, including hydrogen bonds with His353 and/or His513 (cACE) and His711 (NEP) along with metal-chelator interactions^43,80^. The study found that native ligands in protein structure are maximally superimposed with co-crystallized ligands, confirming the docking protocol's agreement with previous work and confirming the interaction of native ligands(Fig. 9)^17,81^. This suggests that the docking methodology is suitable for the virtual screening of dataset compounds.

#### Molecular docking results

The binding affinity results of the standard drug molecules, Omapatrilate against the cACE and NEP enzyme are summarized in Table 4. Conventional ACE inhibitors bind to the catalytic region of the active sites of cACE and NEP via chelation with the central Zn^2+^, while the groups P2′, P1′, P1, and P2, mimicking substrate peptides, are placed inside these subsites. Most of the selected ligands were successfully docked with a plausible pose into the active sites using one of the applied constraint conditions. The resulting docking pose that has metal-acceptor interaction and bonding interactions with His353 and/or His513 (cACE) or bonding with His711 (nACE) is selected for assessment^80^. The results of molecular docking of selected screened designed derivatives with higher or equivalent docking scores compared to standard drugs with their interacting residues are given in Tables S1 to S3 (Supporting file S8).

**Table 4:** Molecular docking results of selected screened designed derivatives against cACE (PDB ID: 1O86) and NEP (PDB ID: 5JMY) enzyme.

ID	Docking score		ID	Docking score		ID	Docking score	
	cACE	NEP		cACE	NEP		cACE	NEP
Chalcone derivatives								
C105	− 5.6880	− 6.0413	C148	− 5.6612	− 7.1215	C101	− 5.8123	− 6.2563
C146	− 5.5313	− 5.8255	C26	− 6.0520	− 6.1498	C99	− 5.7909	− 6.7488
C191	− 5.8248	− 6.1590	C164	− 6.3250	− 6.4206	C24	− 5.9405	− 6.3306
C165	− 5.8290	− 5.9617	C145	− 6.0280	− 6.7257	C93	− 5.6422	− 6.4874
C97	− 6.2998	− 6.2006	C169	− 5.5621	− 6.4222	C98	− 5.8513	− 7.1859
C115	− 5.7009	− 7.1057	C96	− 6.2587	− 5.8640	C102	− 5.9095	− 6.8404
C167	− 6.0243	− 6.2916	C14	− 6.6276	− 6.1051	C229	− 6.4089	− 6.5466
1,3 − Thiazole derivatives								
T1	− 7.7185	− 6.5466	T10	− 4.9357	No pose	T17	− 4.2001	− 4.6230
T3	− 6.9584	− 7.1859	T11	− 5.6430	− 5.8640	T20	No pose	− 4.7410
T9	− 4.7256	− 5.1339	T16	No pose	− 5.1430			
1,3,4 − Thiadiazole derivatives								
TD6	− 5.5672	− 5.1171	TD75	− 7.6001	− 5.3231	TD104	− 8.1032	− 7.5842
TD7	− 5.4932	− 5.2422	TD98	− 9.4353	− 8.7272	TD105	− 5.1424	− 8.3799
TD33	− 6.3711	− 5.8966	TD101	− 0.0854	− 7.4954	TD106	− 5.7235	− 7.7640
TD64	− 6.1358	− 6.1683	TD103	− 5.2566	No pose	TD107	− 7.3480	No pose
Standard reference compounds								
Omapatrilate							− 5.2088	− 5.2360

Chalcone derivatives exhibited varying docking scores for both cACE and NEP (See Supporting file S7). Notably, C115 demonstrated a docking score of − 5.7009 for cACE and − 7.1057 for NEP, while C148 had a docking score of − 5.6612 for cACE and − 7.1215 for NEP. Compound C105, a member of the chalcone derivatives, displayed a favorable docking score of − 5.6880 for cACE. These results suggest variations in the binding affinities of chalcone derivatives to the target enzymes.

Likewise, the 1,3-thiazole derivatives showed a range of docking scores. T1 exhibited a high affinity for the binding of cACE and NEP, with docking scores of − 7.7185 and − 6.5466, respectively. The docking scores for T3 interactions with the enzymes were − 6.9584 for cACE and − 7.1859 for NEP. In comparison, T10 and T20 received a docking score of − 4.9357 and − 4.7410 for NEP and no pose for cACE.

Among the 1,3,4-Thiadiazole derivatives, TD7 displayed a docking score of − 5.4932 for cACE and − 5.2422 for NEP. TD104 also exhibited a strong binding affinity with a docking score of − 8.1032 for cACE and − 7.5842 for NEP. TD98 achieved an impressive docking score of − 9.4353 for cACE and − 8.7272 for NEP. TD101 had a minimal docking score of − 0.0854 for cACE and − 7.4954 for NEP. TD64 had a docking score of − 6.1358 for cACE and − 6.1683 for NEP, while D103 had no pose for cACE. TD107 also had no pose for either cACE or NEP.

**Figure 10:**
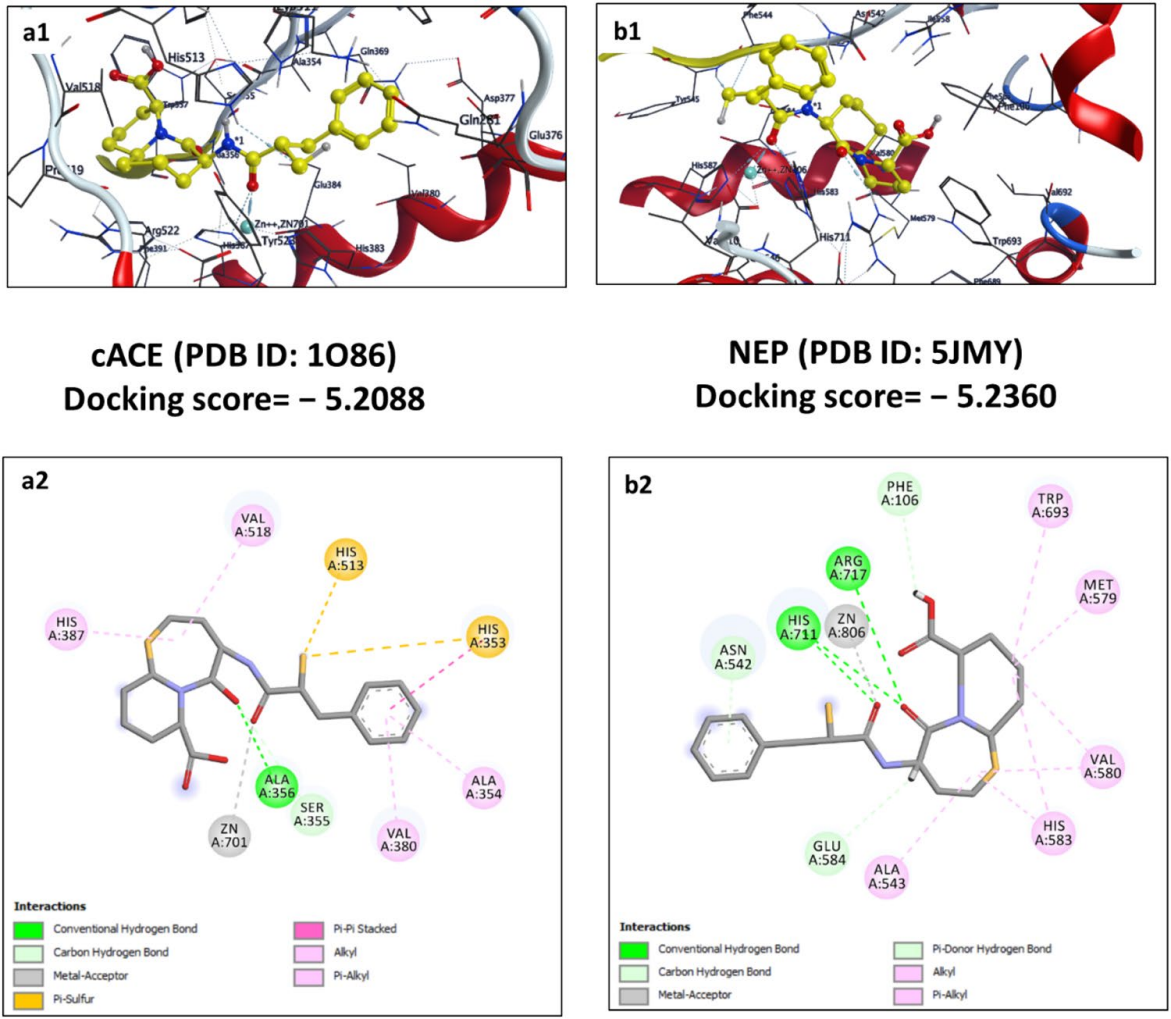
Schematic representation of 2D (**a2** and **b2**) and 3D (**a1** and **b1**) docking poses of standard drug omapatrilate against cACE and NEP target, binding to the catalytic region of the active sites via a chelation interaction with the zinc atom.

**Figure 11:**
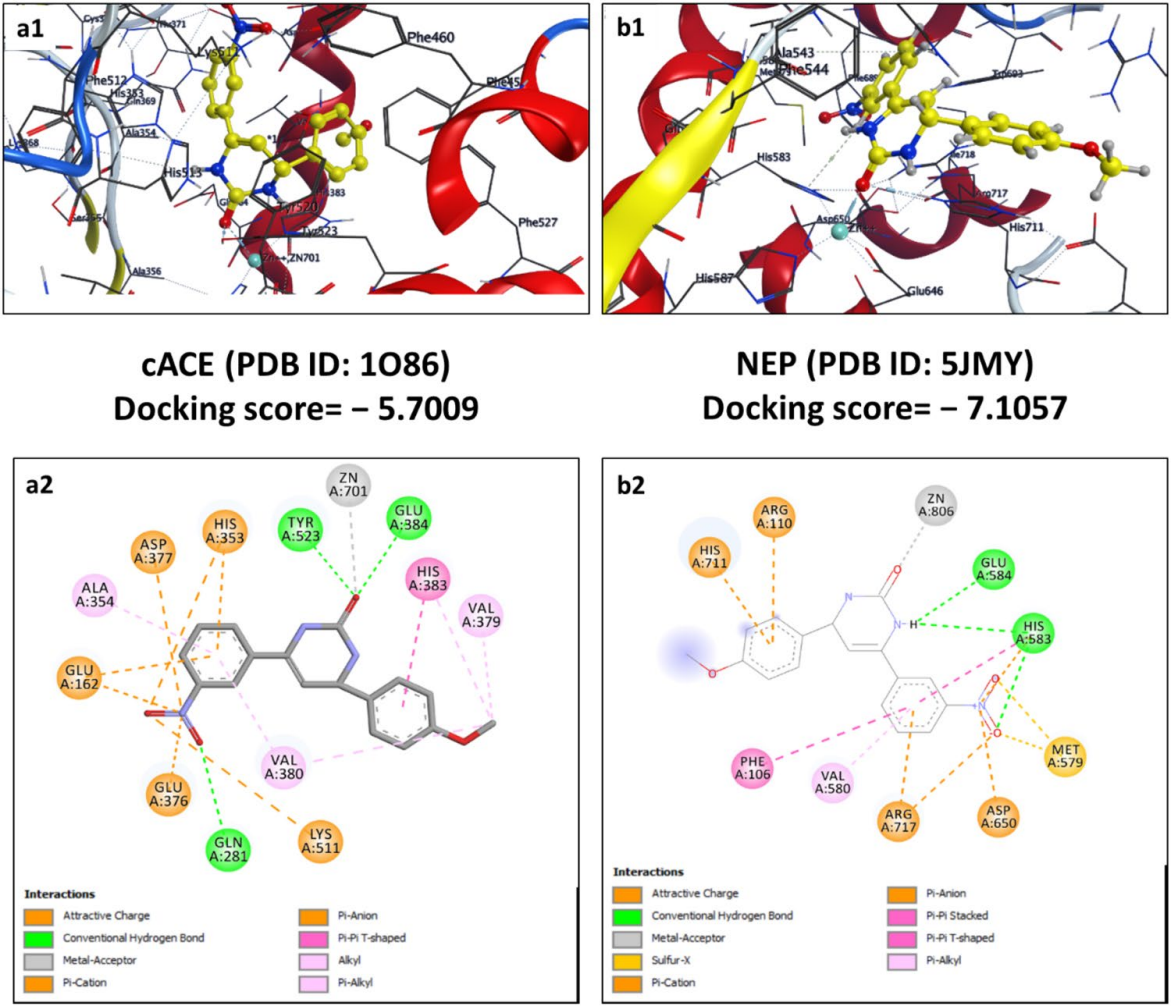
Schematic representation of 2D (**a2** and **b2**) and 3D (**a1** and **b1**) docking poses of Chalcone derivative (C115) against cACE and NEP target, binding to the catalytic region of the active sites via a chelation interaction with the zinc atom.

**Figure 12:**
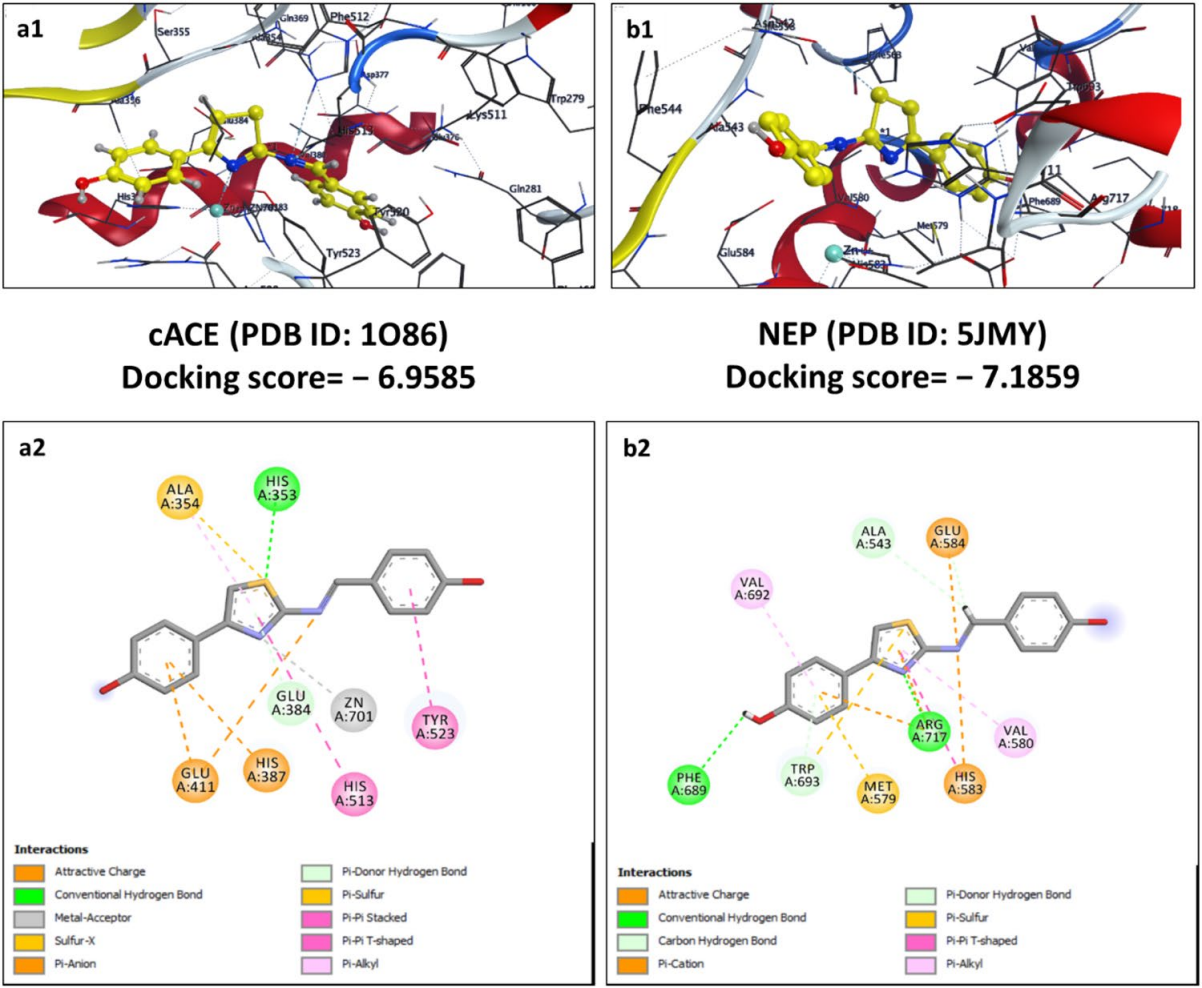
Schematic representation of 2D (**a2** and **b2**) and 3D (**a1** and **b1**) docking poses of 1,3-thiazole derivative (T3) against cACE and NEP target, binding to the catalytic region of the active sites via a chelation interaction with the zinc atom.

**Figure 13:**
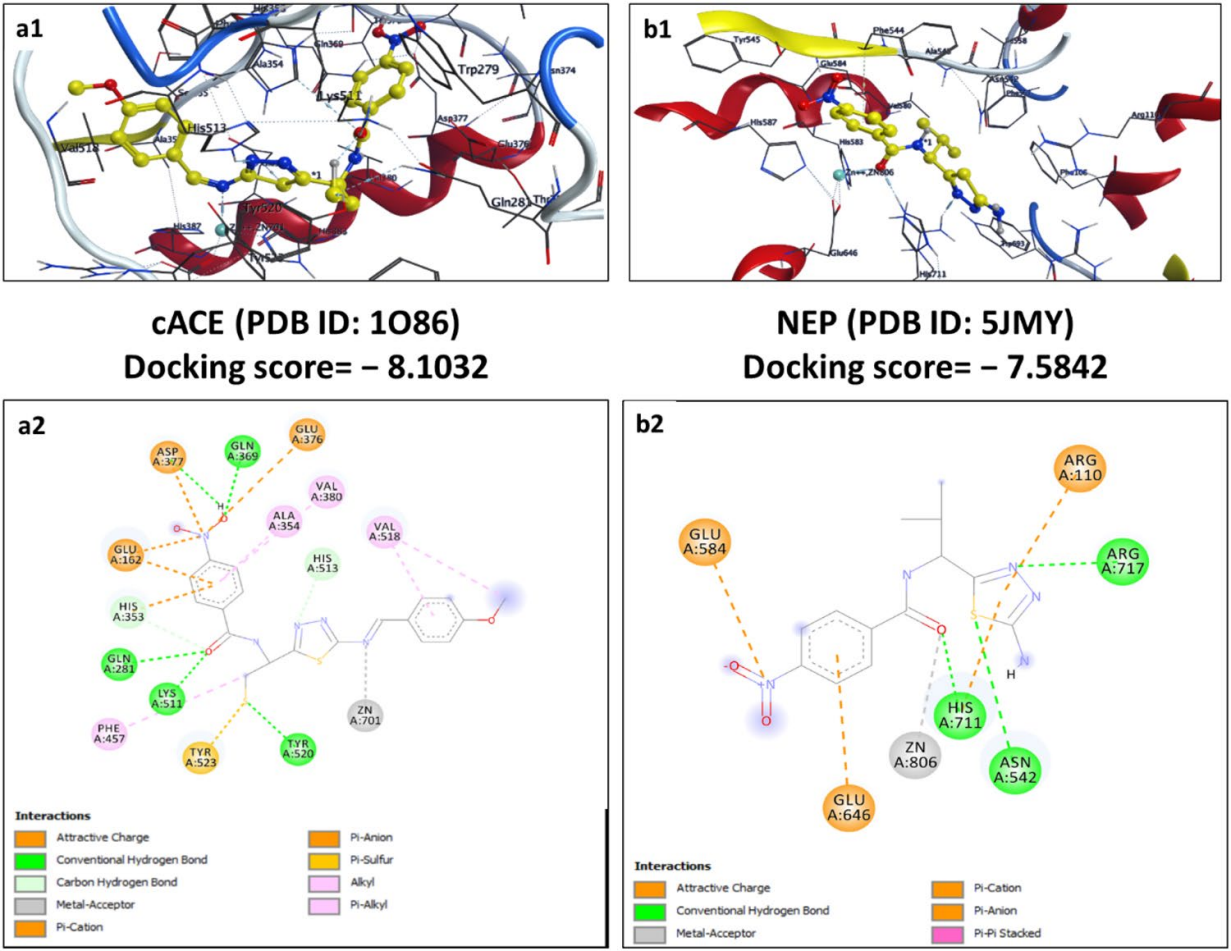
Schematic representation of 2D (**a2** and **b2**) and 3D (**a1** and **b1**) docking poses of 1,3,4-thiadiazole derivative (TD104) against cACE and NEP target, binding to the catalytic region of the active sites via a chelation interaction with the zinc atom.

Visualization of docking poses demonstrated the importance of chelation with the central Zn^2+^, formation of hydrogen (conventional), and hydrophobic (π-π stacking, π-alkyl, and alkyl) interaction required with key residues at S_1_ and S_2_’ for the inhibition both cACE and NEP enzymes. From the docking results, the compounds with functional moiety forming the above interactions appeared to be an ideal scaffold to be dual inhibitors. The final docking poses and binding interactions of selected top ligands which have favorable binding energies and poses are illustrated in Figs. 10, 11, 12, 13.

#### Molecular docking analysis

Omapatrilate is a comprehensively experimented dual ACE/NEP inhibitor showing a binding energy of—5.2088 kcal/mol against cACE because of the metallic interactions with Zn^2+^ and hydrogen bonding interactions with Ala356 and Ser355 through an oxygen atom. Further, the sulfur atom and benzene ring enhanced stability by forming π-sulfur interactions with His513 (subsite S1′) and His353. It also shows alkyl, π-alkyl, and π-π stacked interactions with residue His387, Val518, Val380, Ala354, and His353 respectively. The interaction between the omapatrilat molecule and NEP is particularly stable (binding energy—5.2360 kcal/mol), as seen in Fig. 10. The P1′ carbonyl group of omapatrilat forms a hydrogen bond interaction with Phe106. Although the seven-membered fused ring only partially reaches into the S2′ pocket, it nevertheless interacts hydrophobically with Val580, His583, and Ala543 residue. Both oxygen atoms of the Omapatrilat P2′ peptide bond form metallic interactions with Zn^2+^ and conventional hydrogen bonds with His711 and Arg717 residues.

In the docking simulation between C115 and cACE, it forms three hydrogen bonds with Gln281 (S2’ subsite), Tyr523, and Glu384 residues Fig. 11. It also forms attractive charge interactions (π-cation, π-anion, and others) with five amino acid residues including His353, Asp377, Glu162, Glu376, and Lys511. In addition, four residues (Ala354, Val380, Val379, and His383) were included in hydrophobic interactions (alkyl, π-alkyl, and π-π T-shaped interactions) to give stability to docked pose of the ligand (binding energy,—5.7009 kcal/mol). The molecular interaction pattern of compound C115 showed docking interactions with the NEP enzyme accompanied by a docking score of − 7.1057 kcal/mol. It forms two hydrogen bonds with Glu584 and His583 similar to that of omapatrilate. Further, the stability of the protein–ligand complex, on one hand, is supported by the formation of charged interactions (π-cation, π-anion, and others) with Arg110, His711, Arg717, and Asp650. It also forms hydrophobic interaction with Phe106 (π-π T shaped), and Val580 (π-alkyl) through phenyl ring.

Similar to omapatrilate, compound T3, belonging to the 1,3-thiazole derivative formed two hydrogen bonds with Glu384 (π-donor H-bond) and His353 (conventional H-bond) residues included in the binding pocket. Aside from H-bonds, additional hydrophobic (Glu411 and His387: *π*-anion interactions) and attractive charge interactions (Ala354 residue) were also detected (Fig. 12). On the other hand, hydrophobic cleft formed by π-alkyl, π–π stacked, and π–π T-shaped with His513 and Tyr523 provides additional stability to better fit at the active site of the cACE domain*.* The utmost affinity of T3 with the NEP enzyme accounted for the formation of hydrogen bonds (conventional and carbon-hydrogen) with Arg717, Phe689, Ala543, and Trp693. In addition to hydrogen bonding interaction, ligands form hydrophobic bonding interactions (alkyl, π-alkyl, π-sulfur, and *π–*π T-shaped) with Val580, Met579, and Val692.

The designed 1,3,4-thiadiazole derivative, compound TD104, showed binding affinity − 8.1032 kcal/mol and − 7.5842 kcal/mol with cACE and NEP enzymes respectively. It interacts with amino acids Gln281, Lys511, Tyr520, Gln369, His353, and His513 by forming both conventional and carbon-hydrogen bonds. It also forms charged interactions with Glu376, Asp377, and Glu162 (π-cation, π-anion, and other attractive charge interactions). The formation of hydrophobic interactions (alkyl, π-alkyl, and π-sulfur) with Phe457, Ala354, Val380, Val518, and Tyr523 amino acid residue respectively provides stability to docked poses. The formation of metal-acceptor interaction at the active site of both enzymes again justifies high binding scores for this compound. Compound TD104 also shows favorable binding affinity and forms conventional hydrogen bonding interaction with Arg717, His711, Asn542, and π-charge interaction with Arg110, Glu584, and Glu646 (Fig. 13).

These hydrogen-bonding interactions and hydrophobic interactions between selected designed derivatives (C115, T3, and TD104) and catalytic amino acid [His353 and/or His513 (cACE) and His711 (NEP)] residues of cACE and NEP enzymes make a favorable orientation to interact with zinc ion through metal-chelator interactions. Our docking results indicated that C115, T3, and TD104 could inhibit both targets by inhibiting the active site rather than other secondary sites. Moreover, compound TD104 exhibited greater binding potential for the cACE and NEP compared to C115 and T3.

Further, the selected top ligands that form favorable interactions with targets were screened using ADMET studies. Additionally, molecular dynamics simulations of all selected ligand docking poses were run to verify the key residues (catalytic residue) from docking poses.

### In silico ADME and toxicity studies of selected screened compounds

Prediction of Pharmacokinetic profile (ADME) parameters before experimental studies is among the most vital aspects of the drug design and discovery of drug molecules. These parameters along with toxicity predictions of the compounds are considered important attentive parameters during the transformation of a molecule into a potent drug.

#### Drug-likeness and ADME studies of the selected screened compounds

The drug-likeness capability of compounds can be prophesied using Lipinski, Ghose, Veber, Egan, and Muegge rules which are predicated on specific physicochemical parameters like logP (for oral in range of 1.35–1.8, sub-lingual > 5) tPSA (should be < 140 Å), no. of donors (< 10), acceptors (> 5), etc.^82^. The predicted drug-likeness, PAINS, and synthetic accessibility properties of the selected compounds are shown in Table 5. According to the results, the selected compounds in Table 5 showed no violations of the Lipinski and Ghose rules. However, compounds TD75 and TD104 are acceptable with only one violation according to Veber, Egan, and Muegge rules. All of the different compounds in the PAINS investigation were not exhibiting any alerts, except for compounds C93 and C229.

**Table 5:** Predicted drug-likeness, PAINS study, and synthetic accessibility measures of the screened compounds.

Molecule Id	No. of violations					No. of alerts PAINS	Synthetic accessibility
	Lipinski	Ghose	Veber	Egan	Muegge		
C97	0	0	0	0	0	0	3.31
C105	0	0	0	0	0	0	3.18
C93	0	0	0	0	0	1	3.25
C191	0	0	0	0	0	0	3.32
C146	0	0	0	0	0	0	3.38
C165	0	0	0	0	0	0	3.89
C115	0	0	0	0	0	0	3.73
C229	0	0	0	0	0	1	3.96
T3	0	0	0	0	0	0	2.96
T4	0	0	0	0	0	0	3.05
TD75	0	0	1	1	1	0	3.65
TD104	0	0	1	1	1	0	3.98
TD106	0	0	0	0	0	0	4.03

Table 6 presents the predicted ADME properties of these compounds, including gastrointestinal (GI) absorption, blood–brain barrier permeation (BBB), inhibition of the CYP_450_ system, and permeability glycoprotein (P-gp) substrate. Furthermore, aqueous solubility and BBB values of the ligands preferably lie in the range of − 6.5–0.5 and − 3.0–1.2 respectively^83,84^ Also, p-glycoprotein (P-gp) non-substrate causes drug resistance^85^.

**Table 6:** Predicted pharmacokinetics (ADME) parameters of the screened compounds.

Molecule ID	GI absorption	BBB permeant	Pgp substrate	CYP1A2	CYP2C19	CYP2C9	CYP2D6	CYP3A4	log Kp (cm/s)	Bioavailability score
C97	High	No	No	No	Yes	Yes	No	No	-6.5	0.55
C105	High	Yes	No	No	No	No	No	No	-6.45	0.55
C93	High	Yes	No	No	Yes	No	No	No	-6.45	0.55
C191	High	No	No	No	No	No	No	No	-6.87	0.55
C146	High	No	No	No	No	No	No	No	-6.96	0.55
C165	High	No	Yes	No	No	No	No	No	-7.33	0.56
C115	High	No	Yes	No	No	Yes	No	No	-6.51	0.55
C229	High	No	Yes	No	No	No	No	No	-7.33	0.56
T3	High	No	No	Yes	Yes	Yes	No	No	-5.36	0.55
T4	High	No	No	Yes	Yes	Yes	No	No	-5.07	0.55
TD75	Low	No	No	No	Yes	Yes	No	No	-7.14	0.11
TD104	High	No	No	Yes	Yes	Yes	No	Yes	-6.93	0.55
TD106	Low	No	No	Yes	Yes	Yes	No	Yes	-5.96	0.55

According to the results, all the compounds showed high GI absorption, except for compounds TD75 and TD106. BBB permeation potential was predicted for compounds C93 and C105. Compounds C115, C165, and C229 showed potential to be a substrate of P-gp. The potential to inhibit cytochrome P450 (CYP) isoforms was observed for compounds TD104 and TD106 (for 4 isoforms); T3 and T4 (for 3 isoforms); C97 and TD75 (for 2 isoforms); and C93 (for 1 isoform). Chalcone derivatives C105, C115, C146, C165, C191, and C229 were predicted to show no inhibitory activity against any of the CYP isoforms. The computed bioavailability score for all the compounds placed them within the 55–56% probability class, except for compound TD75 (0.11).

After evaluating drug-likeness, and ADME characteristics, and studying PAINS alerts, the compounds C93, C229, and TD75 have been excluded from consideration for further investigation. A total of 10 compounds are now undergoing additional toxicological studies, utilizing VEGA QSAR and ProTox-II software.

### In silico toxicological results

Predicting the toxicological properties and pharmacokinetic parameters of a compound plays a crucial role in the drug discovery process, as they collectively contribute to 60% of the failures in converting a lead compound into an effective drug^86^.

#### Toxicological properties prediction using VEGA-QSAR models

To evaluate toxicological data, the QSAR modeling method was performed using VEGA-QSAR (https://www.vegahub.eu/portfolio-item/vega-qsar). The software-incorporated algorithm provides the evaluation of reliability prediction as Applicability domain index (ADI) value (Tables 7 and 8). It gives positive results with ADI > 0.5, as indicators of reliability effect; low (0.5 < ADI < 0.6), medium (0.6 < ADI < 0.8), and high (0.8 < ADI < 1).

**Table 7:** Toxicological data of selected designed chalcone derivatives using VEGA-QSAR.

No	Toxicity test	C97	C105	C191	C146	C165	C115
1	Mutagenicity (Ames test) model (CAESAR)						
	Assessment	0.904	0.685	0.735	< 0.5	0.886	0.746
	Prediction	Mutagenic	Mutagenic	Suspect Mutagenic	Mutagenic	NON-Mutagenic	Mutagenic
2	Mutagenicity (Ames test) model (SarPy/IRFMN)						
	Assessment	0.904	0.685	0.735	0.882	0.886	0.691
	Prediction	Mutagenic	Mutagenic	Mutagenic	NON-Mutagenic	NON-Mutagenic	NON-Mutagenic
3	Carcinogenicity model (CAESAR)						
	Assessment	0.758	0.38	0.764	0.369	< 0.5	< 0.5
	Prediction	Carcinogen	Carcinogen	Carcinogen	Carcinogen	Carcinogen	NON-Carcinogen
4	Carcinogenicity model (ISS)						
	Assessment	0.535	< 0.5	0.533	< 0.5	0.446	0.529
	Prediction	Carcinogen	NON-Carcinogen	Carcinogen	NON-Carcinogen	NON-Carcinogen	Carcinogen
5	Developmental/Reproductive toxicity library (PG)						
	Assessment	< 0.5	0.542	0.803	< 0.5	0.377	0.378
	Prediction	NON-Toxicant	NON-Toxicant	NON-Toxicant	NON-Toxicant	NON-Toxicant	NON-Toxicant
6	Estrogen receptor relative binding affinity model (IRFMN)						
	Assessment	0.624	< 0.5	0.618	< 0.5	0.637	0.455
	Prediction	Inactive	Active	Inactive	Inactive	Inactive	Inactive
7	Estrogen receptor-mediated effect (IRFMN/CERAPP)						
	Assessment	0.753	0.623	0.745	0.884	0.529	0.535
	Prediction	Inactive	Inactive	Inactive	Inactive	Inactive	Inactive
8	Androgen receptor-mediated effect (IRFMN/COMPARA) -						
	Assessment	0.887	0.757	0.877	0.886	0.753	0.536
	Prediction	Inactive	Inactive	Inactive	Inactive	Inactive	Inactive
9	Thyroid receptor alpha effect (NRMEA)						
	Assessment	0.908	0.907	0.904	0.903	0.892	0.769
	Prediction	Inactive	Inactive	Inactive	Inactive	Inactive	Inactive
10	Thyroid receptor beta effect (NRMEA)						
	Assessment	0.908	0.907	0.904	0.903	0.892	0.769
	Prediction	Inactive	Inactive	Inactive	Inactive	Inactive	Inactive
11	Skin sensitization model (IRFMN/JRC)						
	Assessment	0.364	0.309	0.358	0.315	< 0.5	0.347
	Prediction	Sensitizer	Sensitizer	Sensitizer	Sensitizer	Sensitizer	Sensitizer
12	Hepatotoxicity model (IRFMN)						
	Assessment	0.635	< 0.5	< 0.5	0.745	Unknown	Unknown
	Prediction	Toxic	Toxic	Toxic	NON-Toxic	Unknown	Unknown

**Table 8:** Toxicological data of selected designed 1,3-thiazole, 1,3,4-thiadiazole derivatives, and standard drug omapatrilate using VEGA-QSAR.

No	Toxicity Test	T3	T4	TD104	TD106	Omapatrilate
1	Mutagenicity (Ames test) model (CAESAR)					
	assessment	0.77	0.72	0.792	0.664	0.916
	prediction	Mutagenic	Mutagenic	NON-Mutagenic	NON-Mutagenic	NON-Mutagenic
2	Mutagenicity (Ames test) model (SarPy/IRFMN) -					
	assessment	< 0.5	< 0.5	0.603	0.791	0.916
	prediction	NON-Mutagenic	NON-Mutagenic	Mutagenic	Mutagenic	NON-Mutagenic
3	Carcinogenicity model (CAESAR)					
	assessment	< 0.5	0.629	0.124	0.368	< 0.5
	prediction	Carcinogen	Carcinogen	NON-Carcinogen	NON-Carcinogen	Carcinogen
4	Carcinogenicity model (ISS)					
	assessment	< 0.5	< 0.5	< 0.5	< 0.5	0.757
	prediction	NON-Carcinogen	NON-Carcinogen	NON-Carcinogen	NON-Carcinogen	NON-Carcinogen
5	Developmental/Reproductive toxicity library (PG)					
	assessment	0.514	< 0.5	< 0.5	< 0.5	< 0.5
	prediction	NON-Toxicant	NON-Toxicant	NON-Toxicant	NON-Toxicant	NON-Toxicant
6	Estrogen receptor relative binding affinity model (IRFMN) -					
	assessment	0.252	0.249	0.204	< 0.5	< 0.5
	prediction	Active	Active	Inactive	Inactive	Inactive
7	Estrogen receptor-mediated effect (IRFMN/CERAPP)					
	assessment	< 0.5	< 0.5	0.743	0.741	0.533
	prediction	Not predicted	Not predicted	Inactive	Inactive	Inactive
8	Androgen receptor-mediated effect (IRFMN/COMPARA) -					
	assessment	0.634	0.749	0.744	0.741	0.539
	prediction	Inactive	Inactive	Inactive	Inactive	Inactive
9	Thyroid receptor alpha effect (NRMEA)					
	assessment	0.899	0.902	0.799	0.796	0.924
	prediction	Inactive	Inactive	Inactive	Inactive	Inactive
10	Thyroid receptor beta effect (NRMEA)					
	assessment	0.899	0.902	0.799	0.796	0.924
	prediction	Inactive	Inactive	Inactive	Inactive	Inactive
11	Skin sensitization model (IRFMN/JRC)					
	assessment	< 0.5	0.246	< 0.5	< 0.5	0.273
	prediction	NON-Sensitizer	Sensitizer	Sensitizer	Sensitizer	Sensitizer
12	Hepatotoxicity model (IRFMN)					
	assessment	Unknown	Unknown	0.743	0.737	0.78
	prediction	Unknown	Unknown	Toxic	Toxic	Toxic

All of the selected chalcone derivatives designed in this study were found to exhibit no developmental toxicity (PG model assessment)^87^, and inactive for estrogen and androgen-mediated effect (IRFMN/CERAPP model)^88,89^. Furthermore, they were confirmed to be non-reactive for Thyroid hormone receptor α/β (NRMEA model). All compounds have the potential for skin sensitivity, though the reliability of this prediction is low. Nevertheless, among them, only compounds C146, C165, and C115 were predicted to be non-mutagenic as indicated either by CAESAR or SarPy/IRFMN model assessment^90^ and non-carcinogenic according to the CAESAR or ISS models assessment^91^ **(**Table 7**).**

Similar to chalcone derivatives, skin sensitization predictions using the IRFMN/JRC model indicated sensitization potential in both thiazole and thiadiazole compounds, except for compound T3. Both thiazole derivatives (T3 and T4) were predicted to be non-mutagenic by the AMES toxicity (SarPy/IRFMN model), non-carcinogenic (ISS model), non-indicative of developmental/reproductive toxicity (PG model), and inactive for both androgen receptor-mediated effects and thyroid receptor effects (IRFMN/CERAPP and NRMEA models). The two selected thiadiazole derivatives and standard drug Omapatrilate pass all screening parameters when evaluated through the applied QSAR model and their assessment scores. However, skin sensitization predictions have indicated sensitization potential in these compounds. Additionally, hepatotoxicity predictions have raised concerns about their potential toxicity** (**Table 8**).**

#### GHS toxicity classification and prediction of LD50 using ProTox-II

The GHS toxicity categorization places thiazole in Class III and the designed derivatives of chalcone and thiadiazole, in Class V. This indicates that compounds may be harmful if swallowed (2000 < LD50 ≤ 5000)^92^. The LD_50_ between 2000–5000 mg/kg indicates a safety range and values showed less potent toxic effects (Table 9).

**Table 9:** GHS toxicity classification of selected designed derivatives.

Compound ID	Smiles	Predicted LD_50_	Predicted toxicity class
C165	O = C1NC(= CC(N1)c1ccc(cc1)O)N1CCCC1C(= O)O	5000 mg/kg	Class V
C115	COc1ccc(cc1)C1NC(= O)NC(= C1)c1cccc(c1)[N +](= O)[O-]	3000 mg/Kg	Class V
T3	Oc1ccc(cc1)/C = N\c1scc(n1)c1ccc(cc1)O	300 mg/kg	Class III
T4	Cc1ccc(cc1)c1csc(n1)/N = C\c1ccc(cc1)O	300 mg/kg	Class III
TD104	COc1ccc(cc1)C = Nc1nnc(s1)C(NC(= O)c1ccc(cc1)[N +](= O)[O])CS	2580 mg/kg	Class V
TD106	CC(C(c1nnc(s1)N = Cc1ccccc1)NC(= O)c1ccc(cc1)[N +](= O)[O])C	2580 mg/kg	Class V

According to the above study, designed compounds C165, C115, T3, T4, TD104, TD106, and Q1934 would make better candidates for further synthesis and development.

### Molecular dynamics and MM-GBSA results

MD simulation studies were carried out to understand the stability of protein–ligand interaction. As discussed earlier, the selected designed compounds with favorable screening properties of AD domain values of mt-QSAR models, docking score, and ADMET were selected for MD simulation studies.

Omapatrilate was considered the standard multi-target inhibitor of ACE and NEP enzymes. Backbone RMSD analysis was evaluated (RMSD difference ≤ 2.0 Å) suggesting the stability of omapatrilate into the binding pocket of cACE (PDB ID: 1O80) and NEP (PDB ID: 5JMY) proteins. When the RMSD data were compared, each simulation including 20 ns revealed stable conformation. It was observed that Omapatrilate formed H-bond with various amino acid residues of cACE such as His353, Ala356, Glu384, Tyr523 and His410 whereas hydrophobic bond interaction with Trp357, Val380, His383, Phe457, Lys511, Phe512, His513, Val518, Tyr520 and Tyr523. The strong affinity of Omapatrilate with cACE was observed due to the formation of ionic interactions with amino acid residues His383, His387, His353, and Glu411. Additionally, it also forms salt bridge interaction with Glu143, His353, Ala354, Trp357, Glu403, and Pro519 with protein.

**Figure 14:**
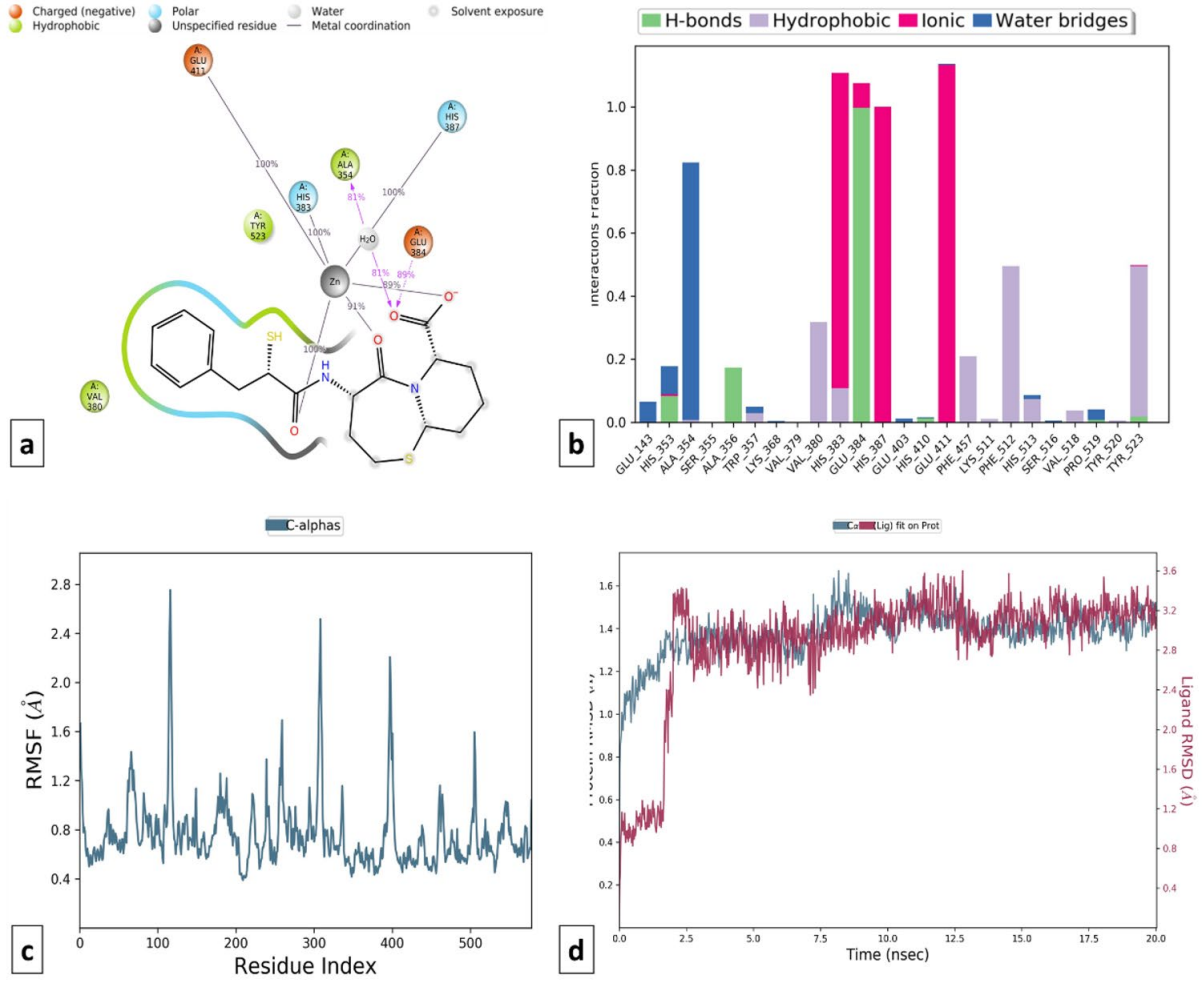
MD simulation analysis of Omapatrilate-cACE complex (**a**) Simulation interactions diagram (**b**) Protein–ligand contacts histogram (**c**) RMSF of the amino acids comprising the cACE (**d**) RMSD of the protein backbone.

**Figure 15:**
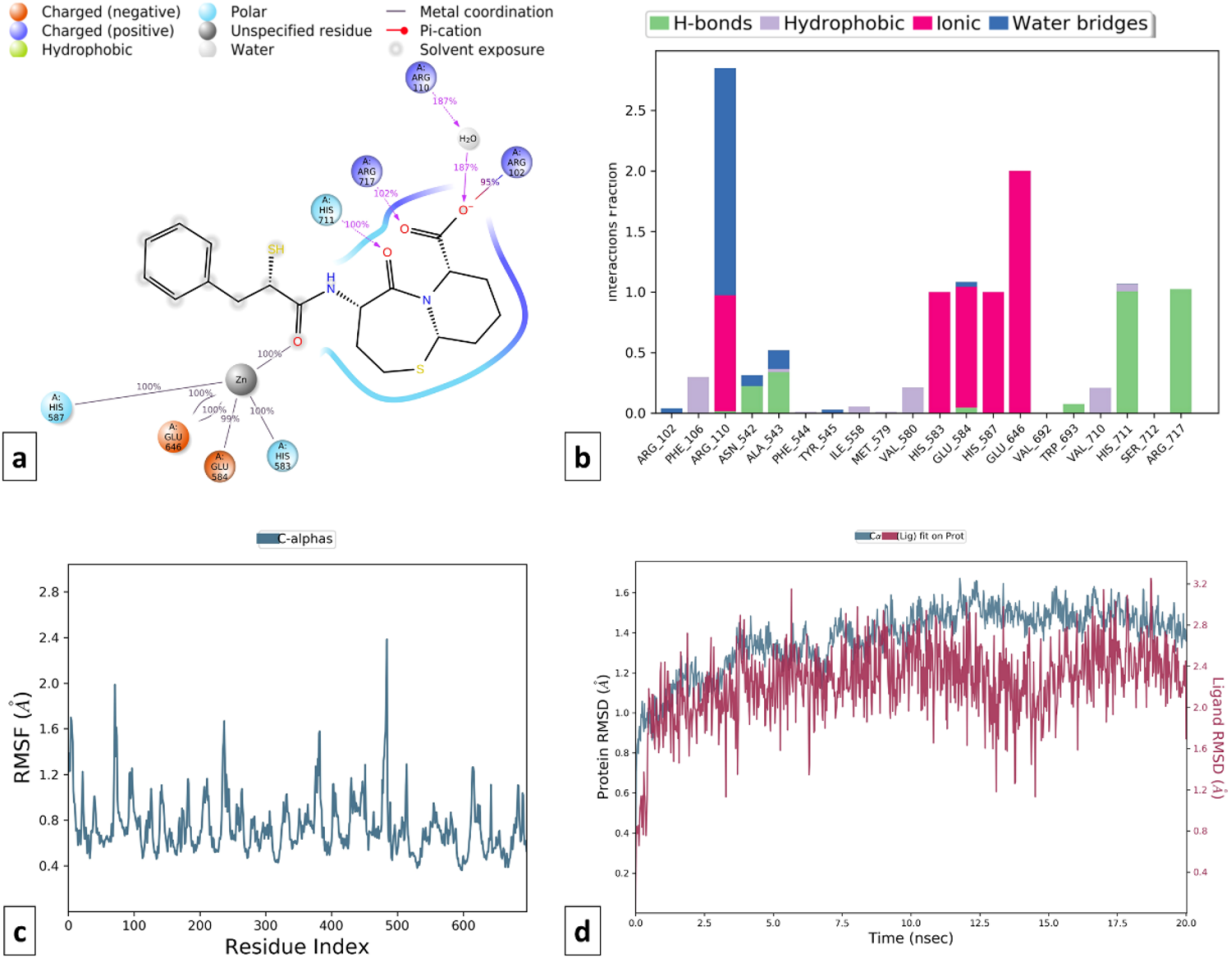
MD simulation analysis of Omapatrilate-NEP enzyme complex (**a**) Simulation interactions diagram (**b**) Protein–ligand contacts histogram (**c**) RMSF of the amino acids comprising the NEP enzyme (**d**) RMSD of the protein backbone.

Similarly, MD simulation of the Omapatrilate-NEP complex reveals the formation of strong ionic interactions with Arg110, His583, Glu584, His587, Glu646 and hydrogen bonding interactions with Asn542, Ala542, Ala543, Glu584, Trp693, His711, Arg717 amino acid residues. The formation of a few hydrophobic interactions with Phe106, Ile558, Val580, and Val710 favored the stability of the complex. Apart from RMSD, the RMSF value of a protein is widely used to access ligand-induced changes in the protein’s internal chains. Figures 14 and 15 show the RMSF plot of the Omapatrilate in complex with cACE and NEP enzymes, respectively.

Likewise, ligand–protein interactions of the selected designed compounds were monitored during the same time trajectory simulation. There are four different categories of contacts viz. as hydrogen bonds, hydrophobic, ionic, and water bridges. The simulation interactions diagram represents more specific subtypes of interactions. Ligands who have maintained contact that occur 10.0% or more of the simulation time are discussed further. Although hydrophobic and hydrogen bonds are weaker compared to ionic bonds, they are too exploited most for the design of new drug candidates^93,94^.

**Figure 16:**
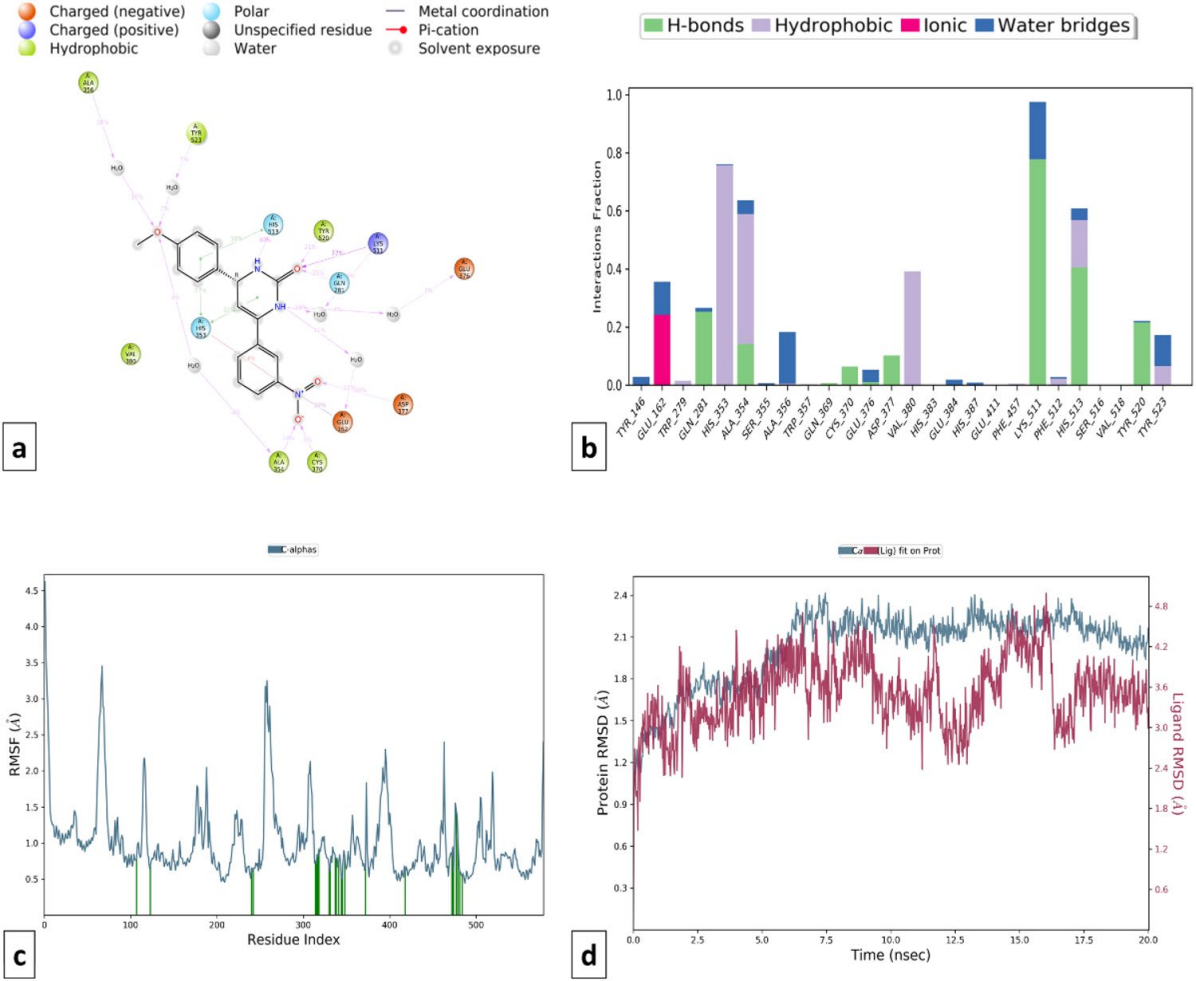
MD simulation analysis of compound C115-cACE complex (**a**) Simulation interactions diagram (**b**) Protein–ligand contacts histogram (**c**) RMSF of the amino acids comprising the cACE (**d**) RMSD of the protein backbone.

**Figure 17:**
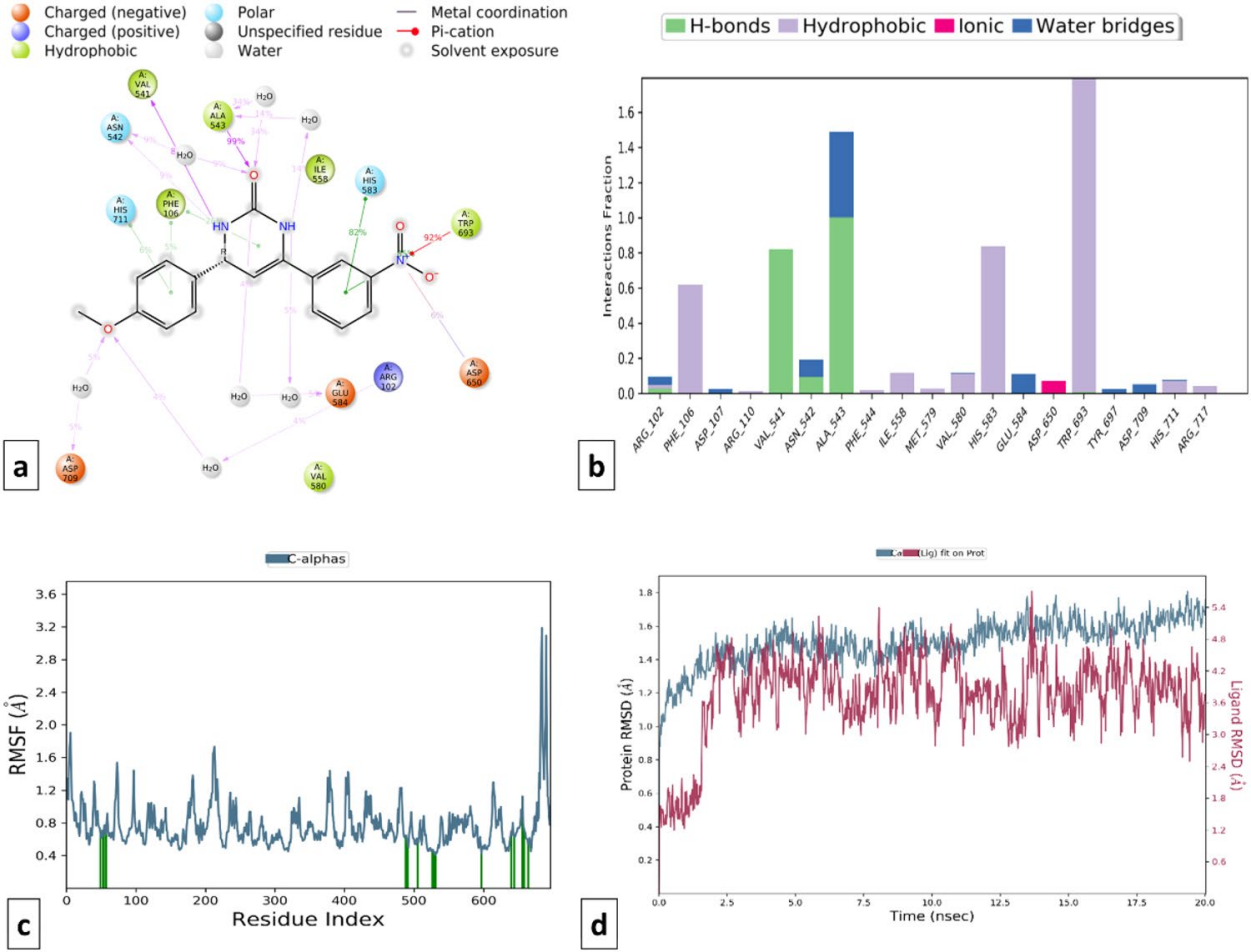
MD simulation analysis of compound C115-NEP enzyme complex (**a**) Simulation interactions diagram (**b**) Protein–ligand contacts histogram (**c**) RMSF of the amino acids comprising the NEP enzyme (**d**) RMSD of the protein backbone.

Ligand interactions of compound C115 at different time intervals were analyzed and checked for stability which showed that the proteins got stabilized and the ligand was forming interaction with the protein (RMSD difference ≤ 2.5 Å). In the cACE domain, C115 was forming H-bond interactions with Gln281, Ala354, Asp377, Lys511, His513, and Tyr520 amino acid residues while hydrophobic interactions with His353, Ala354, Val380, and His513 amino acid residues. Moreover, the salt bridge interaction with Ala356 and Tyr523 gives additional stability to the complex. The RMSD and RMSF plots of protein–ligand and the ligand–protein contacts for compound C115-cACE and compound C115-NEP enzyme complexes are shown in Figs. 16 and 17 respectively. On the other hand, compound C115-NEP complex stabilized by hydrogen bond formation with key amino acid residues Asn 542, and Ala543. Amino acid residues Phe106, Ile558, Val580, His583 and Trp 693 in the S1’ subsites contribute to stability by forming important hydrophobic interactions similar to that of the standard omapatrilate.

**Figure 18:**
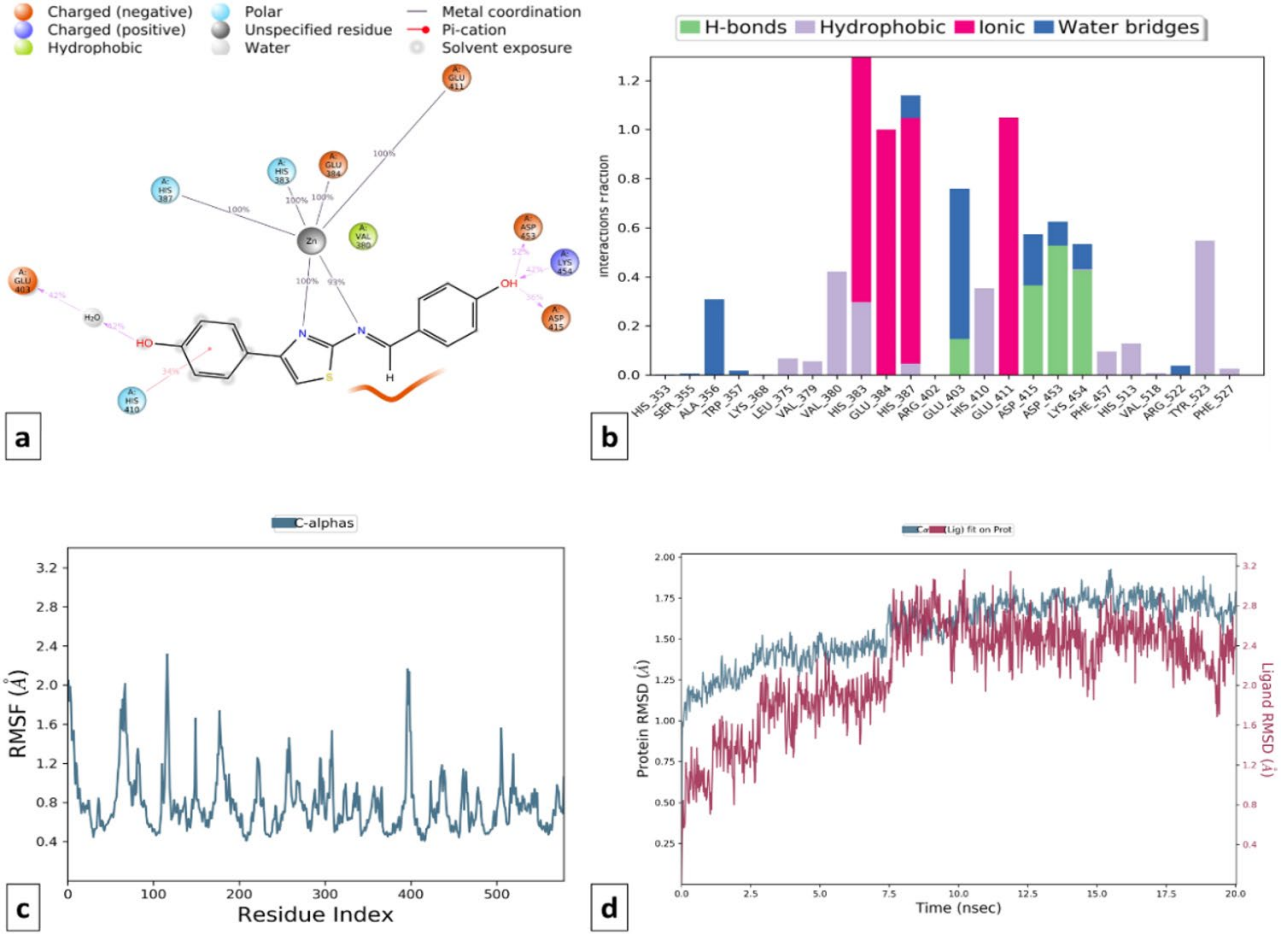
MD simulation analysis of compound T3-cACE complex (**a**) Simulation interactions diagram (**b**) Protein–ligand contacts histogram (**c**) RMSF of the amino acids comprising the cACE (**d**) RMSD of the protein backbone.

**Figure 19:**
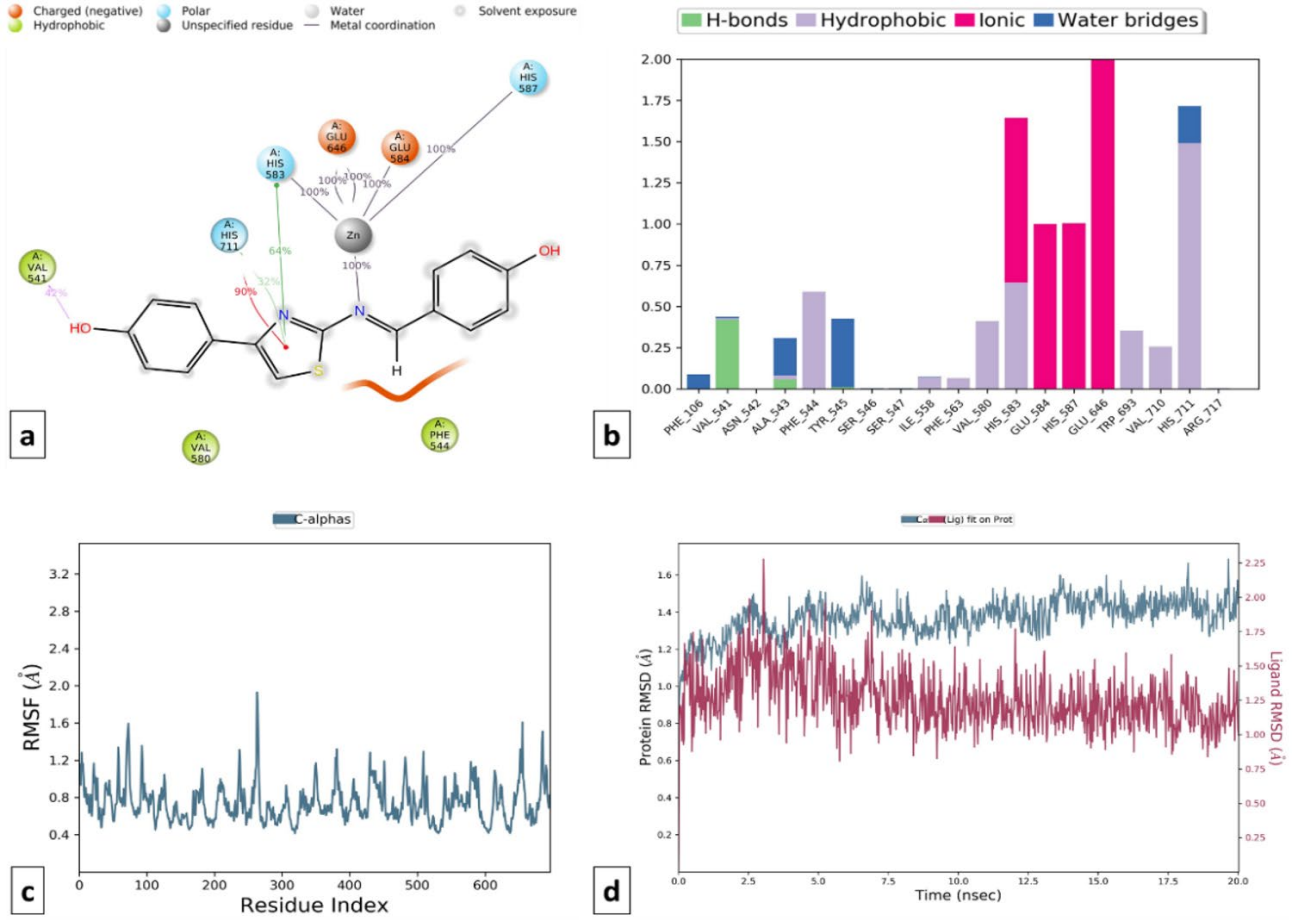
MD simulation analysis of compound T3-NEP enzyme complex (**a**) Simulation interactions diagram (**b**) Protein–ligand contacts histogram (**c**) RMSF of the amino acids comprising the NEP enzyme (**d**) RMSD of the protein backbone.

On the other hand, compound T3 showed stable interactions throughout the simulation period (20.0 ns) which indicates the stability of the ligand in the binding site pocket of the protein (RMSD difference ≤ 2.0 Å). T3 was linked to amino acids like His383, Glu384, His387, and Glu411 by a metal ion coordinated within 3.4 Å of the protein's and ligand's atoms. Additionally, the selectivity towards the cACE domain is due to the formation of hydrogen bonding with Glu403, Asp415, Asp453, and Lys454 and hydrophobic interaction with Val380, His383, His513, and Tyr523 amino acid residues. The simulation interactions of the T3-NEP enzyme complex show that, the ligand occupies tightly in the S1 and S2 pocket of the enzyme through ionic interactions with Glu584, His587, and Glu646 amino acid residue. It also forms, an important hydrophobic interaction with catalytic residue His711 amino acid. Unlike, Ompatrilate the stability is contributed by the formation of multiple type contacts with His583, Val541, Ala543, and Tyr545 amino acid residue (Figs. 18 and 19).

**Figure 20:**
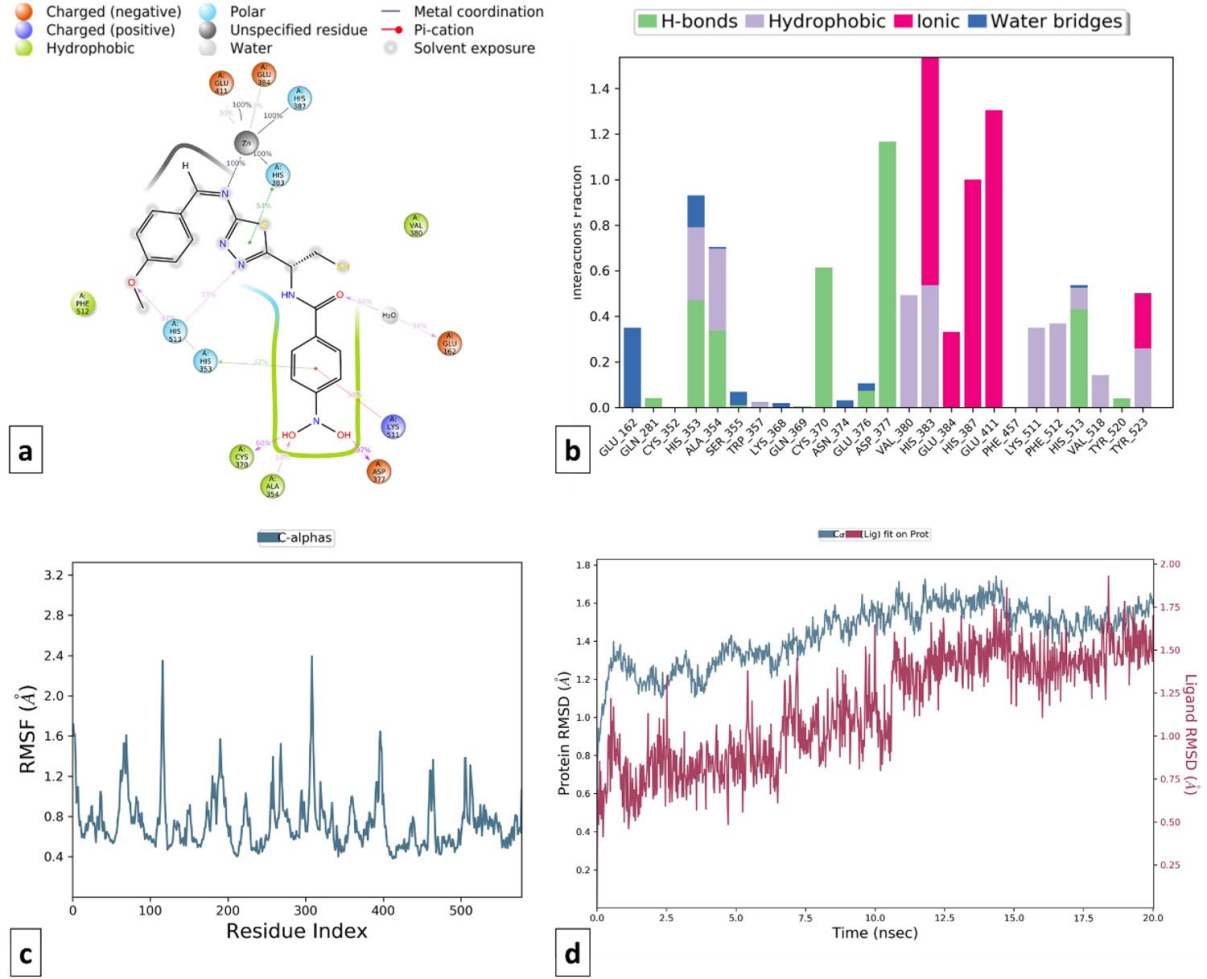
MD simulation analysis of compound TD104-cACE complex (**a**) Simulation interactions diagram (**b**) Protein–ligand contacts histogram (**c**) RMSF of the amino acids comprising the cACE (**d**) RMSD of the protein backbone.

**Figure 21:**
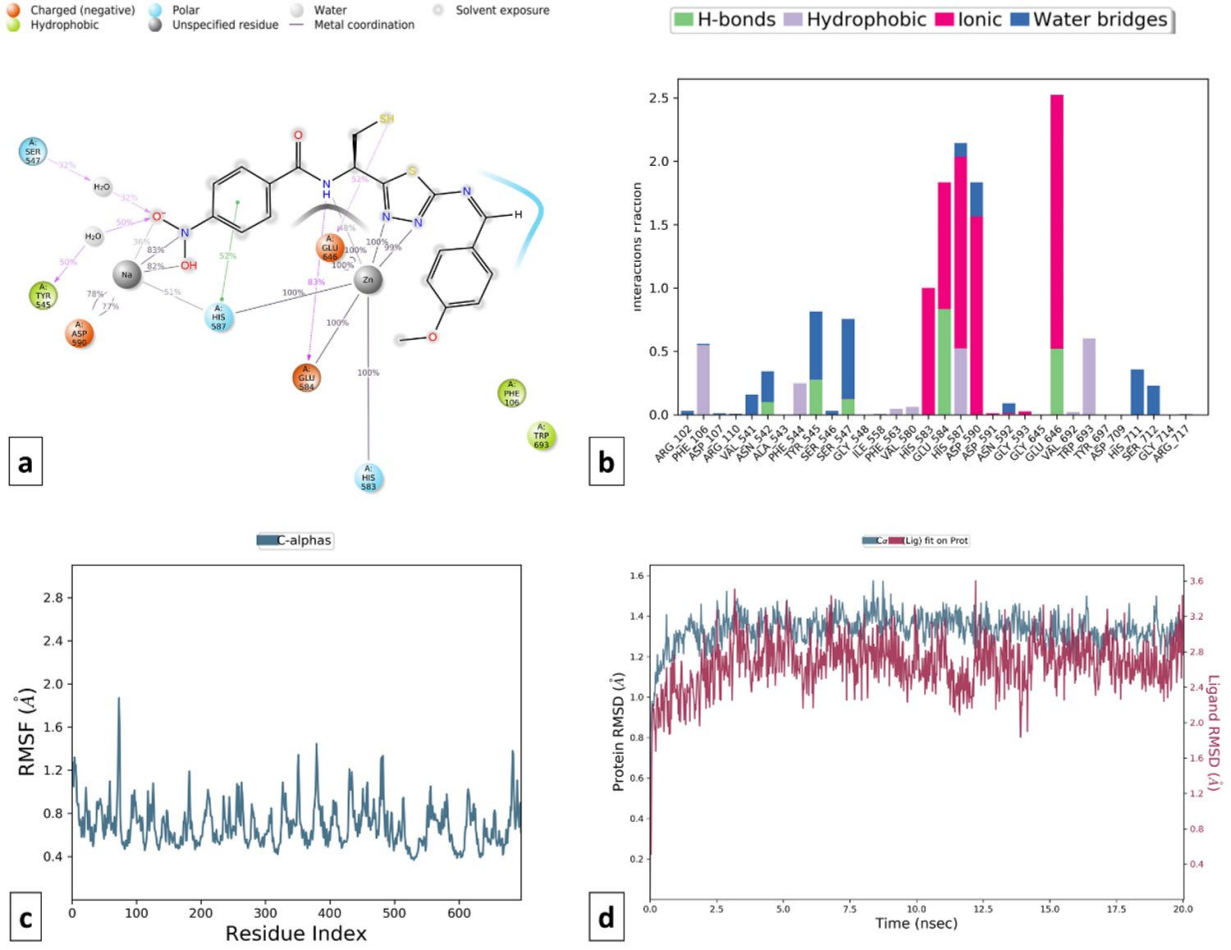
MD simulation analysis of compound TD104-NEP enzyme complex (**a**) Simulation interactions diagram (**b**) Protein–ligand contacts histogram (**c**) RMSF of the amino acids comprising the NEP enzyme (**d**) RMSD of the protein backbone.

MD simulations trajectories revealed that compound TD104 was well stabilized **(**RMSD difference ≤ 2.0 Å) and made favorable metal ionic contacts and hydrogen bonding with an important catalytic site responsible for inhibition of cACE when compared to compound T3 over the entire simulation trajectory. Along with this Gln281, Ala354, Cys370, Asp377, and His513 amino acid residues play key roles in docked pose stability via H-bonding interaction. Also, hydrophobic contacts with Val380, His383, Lys511, Tyr523, and Phe512 are well maintained during the simulation trajectory. Correspondingly, compound TD104 shows the formation of hydrophobic interaction with Phe106, Trp693, and water bridge interaction with His711, and Asn542 in the catalytic domain suggesting its NEP enzyme inhibition probability. Furthermore, stability is provided by ionic interaction made by His583, His587, Glu584, Asp590, and Glu646 amino acid residues **(**Figs. 20 and 21**)**.

Design compounds, C115, T3, and TD104 were found to form significant key interactions similar to omapatrilate towards the substrate binding pocket of cACE and NEP enzymes, and could probably be the novel lead molecules for multi-target inhibition against the target enzymes.

So, the binding potential scores derived from the results of constraint-based docking provide a strong foundation for the MD simulation trajectories. Inferring that the compounds C115, T3, and TD104 have a strong affinity for the cACE and NEP enzymes. Following Lipinski, Ghose, Veber, Egan, and Muegge's rule, all three compounds exhibited acceptable drug-likeness parameters. Furthermore, selected designed compounds (C115, T3, and TD104) do not have AMES mutagenicity and are Non-carcinogenic., no developmental/reproductive toxicity (PG model), and are found to be inactive for hormone receptors (estrogen, androgen, and thyroid α/β). All these parameters suggest compounds are safe to use. Hence, they could be considered for further synthesis and subsequent screening as promising scaffolds for the development of dual inhibitors targeting cACE and NEP enzymes, potentially contributing to the management of hypertension.

The study conducted MM-GBSA calculations to estimate ligand binding energies or affinity (dG Bind), with the results presented in Table 10.

**Table 10:** MM-GBSA values for the selected designed compounds.

Compound ID	MM-GBSA dG Bind (Kcal/mol)	
	cACE	NEP
Omapatrilate	− 15.706	− 7.191
C115	− 16.458	− 8.267
T3	− 12.186	− 5.465
TD104	− 27.755	− 5.574

Based on the MM-GBSA calculations, the ΔG bind values for molecules such as C115, T3, and TD104 were found to be − 16.46, − 12.19, and − 27.76, respectively, for the cACE protein. These binding energies of T3 and C115 closely resemble those of Omapatrilate, indicating a strong binding affinity to the cACE enzyme. Additionally, the ΔG values against NEP were − 8.267, − 5.465, and − 5.574 for the compounds C115, T3, and TD104, respectively, which are in proximity to the ΔG values of Omapatrilate.

Based on the two findings, TD104 and C115 exhibited the highest binding affinities for cACE, with C115 demonstrating strong binding with NEP. Hence, the designed compounds C115 and TD104 show promising potential as novel therapeutics and could serve as valuable leads for the synthesis of cardiovascular agents.

## Conclusion

The study demonstrates the effectiveness of* in-silico*-based mt-QSAR modeling for recognizing structural prerequisites and identifying potential ligands against multiple biological targets under varied experimental conditions. The combinatorial library of three different scaffolds viz. Chalcone and its analogue, 1,3-Thiazole, and 1,3,4-Thiadiazole derivatives were designed and screened utilizing developed classification-based (LDA and RF) QSAR models to get probably potent ligands. Among these, Chalcone (85 compounds), 1,3-Thiazole (8 compounds), and 1,3,4-Thiadiazole (37 compounds) derivatives were identified as potentially effective and potent. Subsequently, molecular docking studies were conducted to understand their molecular interaction capabilities. Molecular simulations confirmed their interaction capabilities, with 13 compounds showing favorable binding scores. Furthermore, ADMET screening was performed on the selected screened molecule, revealing seven identified compounds (C165, C115, T3, T4, TD104, and TD106) that had favorable pharmacokinetic and toxicological profiles, paving the way for subsequent synthesis. The study also suggests that Chalcone and 1,3,4-Thiadiazole scaffolds hold promise for developing multi-targeted cardiovascular agents. Considering further, the research points to the future potential of Chalcone and 1,3,4-Thiadiazole derivatives in developing next-generation multi-target inhibitors. Modifying these scaffolds could lead to the creation of novel molecules with enhanced dual-system blocking activity. The insights gained from this study provide valuable guidance for prospective researchers and medicinal chemists, emphasizing the importance of target-specific binding assays and structure–activity relationship studies to unravel the mechanisms of action and key interactions for multi-target inhibition.

## Supplementary Information

Supplementary Legends.

Supplementary Information 1.

Supplementary Information 2.

Supplementary Information 3.

Supplementary Information 4.

Supplementary Information 5.

Supplementary Information 6.

Supplementary Information 7.

Supplementary Tables.

Supplementary Information 8.

## Data Availability

The data of molecules used in the present work are available as Supporting Information as file name ‘S1’in. xlsx format.
