# Author Correction: Multi-target in-silico modeling strategies to discover novel angiotensin converting enzyme and neprilysin dual inhibitors

**DOI:** 10.1038/s41598-024-71642-6

**Published:** 2024-09-05

**Authors:** Sapan K. Shah, Dinesh R. Chaple, Vijay H. Masand, Rahul D. Jawarkar, Somdatta Chaudhari, A. Abiramasundari, Magdi E. A. Zaki, Sami A. Al‑Hussain

**Affiliations:** 1 Department of Pharmaceutical Chemistry, Priyadarshini J. L. College of Pharmacy, Hingna Road, Nagpur, Maharashtra 440016 India; 2 Department of Chemistry, Vidya Bharati Mahavidyalaya, Amravati, Maharashtra 444602 India; 3 Department of Medicinal Chemistry and Drug Discovery, Dr. Rajendra Gode Institute of Pharmacy, University Mardi Road, Amravati, 444603 India; 4 Department of Pharmaceutical Chemistry, Modern College of Pharmacy, Nigdi, Pune, India; 5 Biobay, Ahmedabad, India; 6 Department of Chemistry, College of Science, Imam Mohammad Ibn Saud Islamic University, 11623 Riyadh, Saudi Arabia

Correction to: *Scientific Reports* 10.1038/s41598-024-66230-7, published online 10 July 2024

The Funding section in the original version of this Article was incorrect.

“Author Magdi Zaki would like to declare the work is funded by the Deanship of Scientific Research at Imam Mohammad Ibn Saud Islamic University.”

now reads:

“This work was supported by the Deanship of Scientific Research at Imam Mohammad Ibn Saud Islamic University (IMSIU).”

In addition, the original version of this Article contained an error in the spelling of the author Dinesh R. Chaple which was incorrectly given as Dinesh D. Chaple.

The original article has been corrected.

