# Supplementary material for: Multi-Target In-Silico modeling strategies to discover novel angiotensin converting enzyme and neprilysin dual inhibitors
Source: Sci Rep. 2024 Jul 10;14:15991. doi: 10.1038/s41598-024-66230-7 (PMC11237057; doi:10.1038/s41598-024-66230-7)
Supplement: Supplementary file 1 — Supplementary Legends. [file 41598_2024_66230_MOESM1_ESM.docx]

[Figure 1. Sequential steps involved in the development of mt-QSAR models for dual endpoint detection (ACE and NEP enzyme inhibition)](#_Toc168580245)

[Figure 2: Scheme utilized for designing of novel chalcone derivatives, thiazole derivatives, and thiadiazole derivatives](#_Toc168580246)

[Figure 3: Crystal structures of the selected target for molecular simulations study i) cACE (PDB ID: 1O86) ii) ii) NEP (PDB ID: 5JMY)](#_Toc168580247)

[Figure 4. An illustration of the metal chelation and positional constraints created and implemented for docking chemical compounds against cACE (PDB ID: 1o86) and NEP (PDB ID: 5JMY).](#_Toc168580248)

[Figure 5. ROC (using 10-fold cross-validation) plots for the best LDA model for dual inhibition of ACE and NEP enzymes](#_Toc168580249)

[Figure 6. Y-randomization test results for the developed LDA model for dual inhibition of ACE and NEP enzymes](#_Toc168580250)

[Figure 7. ROC (using 10-fold cross-validation) plots for the best RF model](#_Toc168580251)

[Figure 8. Screening of designed compounds using developed mt-QSAR models (LDA and RF). Compounds found active through both models are only selected for molecular docking study](#_Toc168580252)

[Figure 9. Validation of docking protocol by re-docking the native ligands (Lisinopril and LBQ657) at active binding site and interacting amino acid residues (Magenta color original poses, green color redocked pose of lisinopril and cyan color redocked pose of LBQ657)](#_Toc168580253)

[Figure 10. Schematic representation of 2D (a2 and b2) and 3D (a1 and b1) docking poses of standard drug omapatrilate against cACE and NEP target, binding to the catalytic region of the active sites via a chelation interaction with the zinc atom.](#_Toc168580254)

[Figure 11. Schematic representation of 2D (a2 and b2) and 3D (a1 and b1) docking poses of Chalcone derivative (C115) against cACE and NEP target, binding to the catalytic region of the active sites via a chelation interaction with the zinc atom.](#_Toc168580255)

[Figure 12. Schematic representation of 2D (a2 and b2) and 3D (a1 and b1) docking poses of 1,3-thiazole derivative (T3) against cACE and NEP target, binding to the catalytic region of the active sites via a chelation interaction with the zinc atom.](#_Toc168580256)

[Figure 13. Schematic representation of 2D (a2 and b2) and 3D (a1 and b1) docking poses of 1,3,4-thiadiazole derivative (TD104) against cACE and NEP target, binding to the catalytic region of the active sites via a chelation interaction with the zinc atom.](#_Toc168580257)

[Figure 14. MD simulation analysis of Omapatrilate-cACE complex a) Simulation interactions diagram b) Protein-ligand contacts histogram c) RMSF of the amino acids comprising the cACE d) RMSD of the protein backbone](#_Toc168580258)

[Figure 15. MD simulation analysis of Omapatrilate-NEP enzyme complex a) Simulation interactions diagram b) Protein-ligand contacts histogram c) RMSF of the amino acids comprising the NEP enzyme d) RMSD of the protein backbone](#_Toc168580259)

[Figure 16. MD simulation analysis of compound C115-cACE complex a) Simulation interactions diagram b) Protein-ligand contacts histogram c) RMSF of the amino acids comprising the cACE d) RMSD of the protein backbone](#_Toc168580260)

[Figure 17. MD simulation analysis of compound C115-NEP enzyme complex a) Simulation interactions diagram b) Protein-ligand contacts histogram c) RMSF of the amino acids comprising the NEP enzyme d) RMSD of the protein backbone](#_Toc168580261)

[Figure 18. MD simulation analysis of compound T3-cACE complex a) Simulation interactions diagram b) Protein-ligand contacts histogram c) RMSF of the amino acids comprising the cACE d) RMSD of the protein backbone](#_Toc168580262)

[Figure 19. MD simulation analysis of compound T3-NEP enzyme complex a) Simulation interactions diagram b) Protein-ligand contacts histogram c) RMSF of the amino acids comprising the NEP enzyme d) RMSD of the protein backbone](#_Toc168580263)

[Figure 20. MD simulation analysis of compound TD104-cACE complex a) Simulation interactions diagram b) Protein-ligand contacts histogram c) RMSF of the amino acids comprising the cACE d) RMSD of the protein backbone](#_Toc168580264)

[Figure 21. MD simulation analysis of compound TD104-NEP enzyme complex a) Simulation interactions diagram b) Protein-ligand contacts histogram c) RMSF of the amino acids comprising the NEP enzyme d) RMSD of the protein backbone](#_Toc168580265)
