# Supplementary material for: Multi-Target In-Silico modeling strategies to discover novel angiotensin converting enzyme and neprilysin dual inhibitors
Source: Sci Rep. 2024 Jul 10;14:15991. doi: 10.1038/s41598-024-66230-7 (PMC11237057; doi:10.1038/s41598-024-66230-7)
Supplement: Supplementary file 9 — Supplementary Tables. [file 41598_2024_66230_MOESM9_ESM.docx]

**Table S1. Molecular docking results of selected screened designed Chalcone Derivatives against cACE (PDB ID: 1O86) and NEP (PDB ID: 5JMY) enzyme**

| **Sr. No.** | **ID** | **Docking score against cACE** | **2D Interaction Diagram of ligands with cACE enzyme** | **Docking score against NEP** | **2D Interaction Diagram of ligands with NEP enzyme** |
| --- | --- | --- | --- | --- | --- |
| **1** | **C14** | −6.6276 | 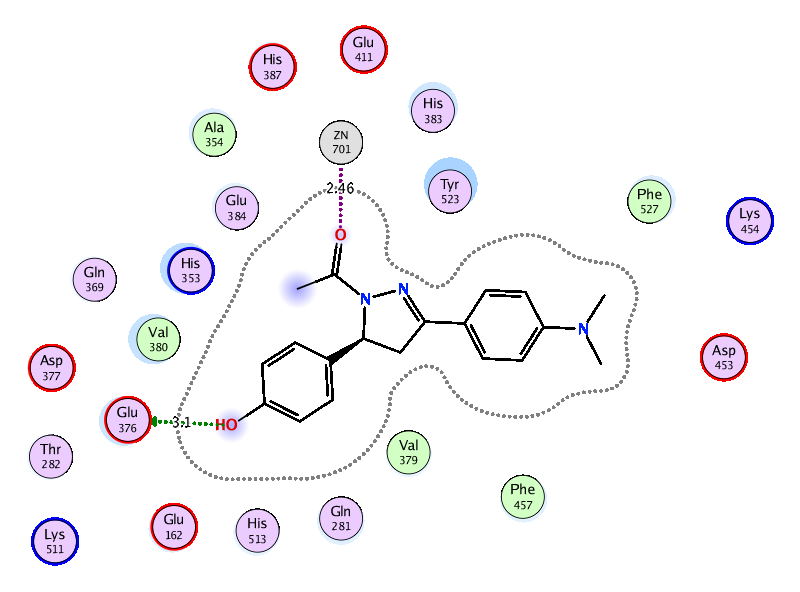 | −6.1051 | 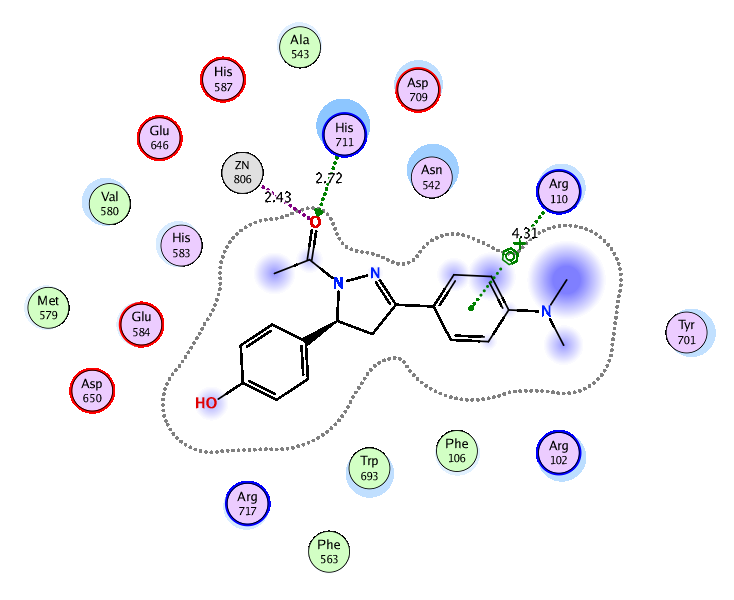 |
| **2** | **C24** | −5.9405 | 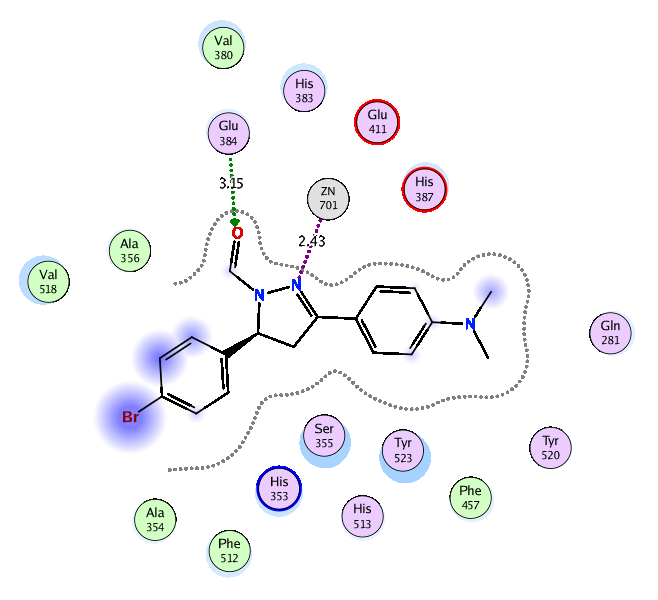 | −6.3306 | 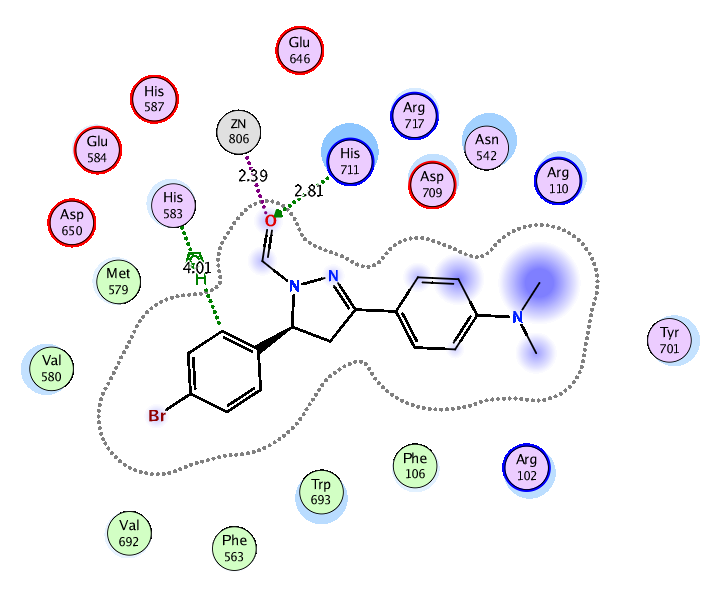 |
| **3** | **C26** | −6.0520 | 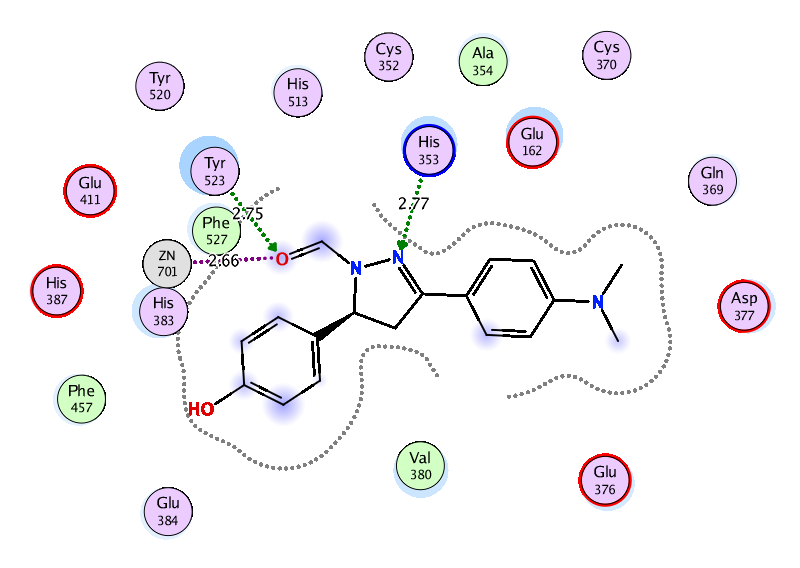 | −6.1498 | 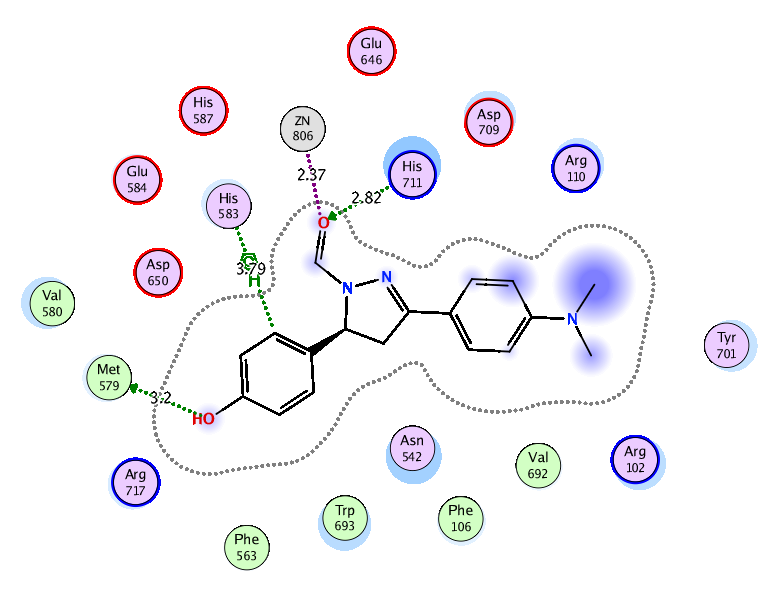 |
| **4** | **C93** | −5.6422 | 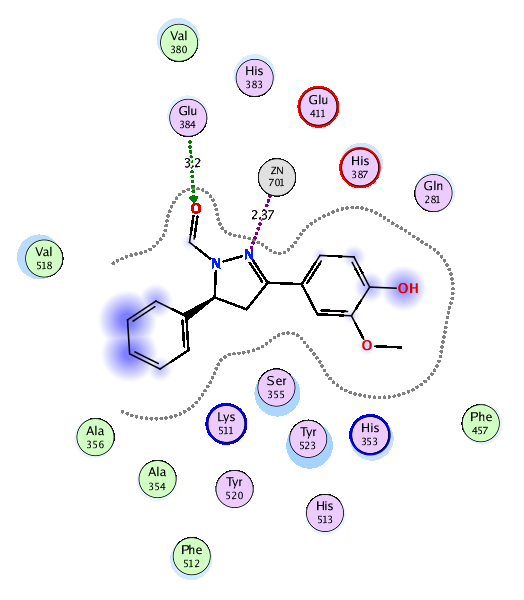 | −6.4874 | 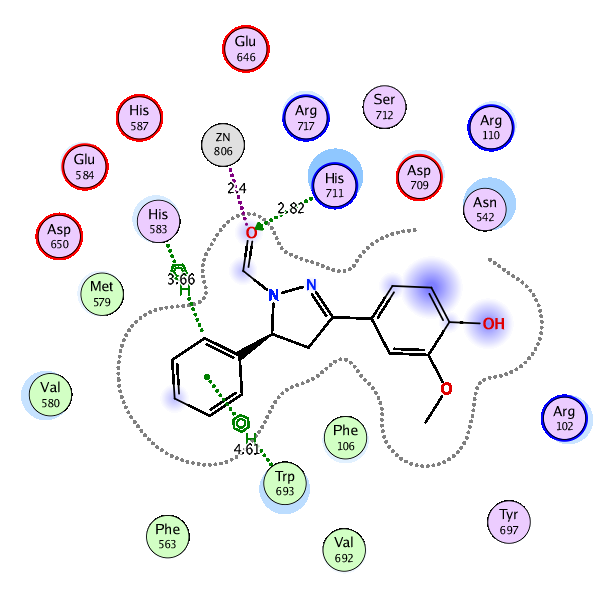 |
| **5** | **C96** | −6.2587 | 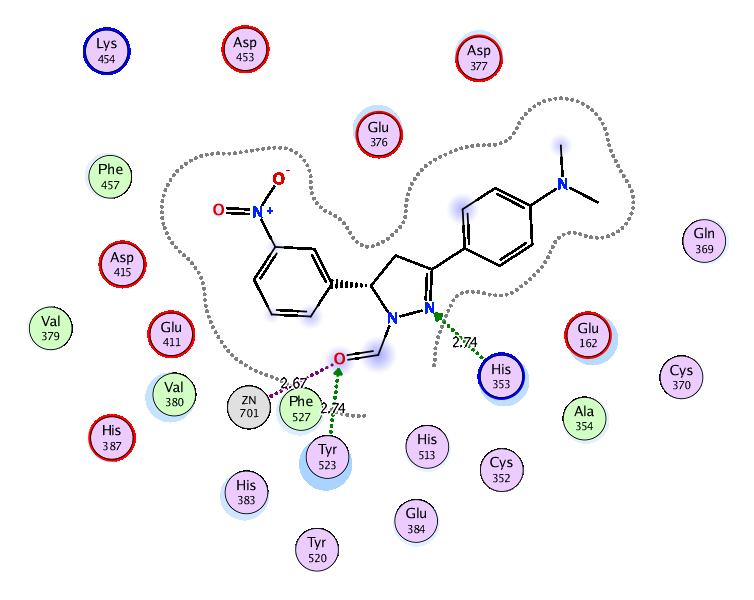 | −5.8640 | 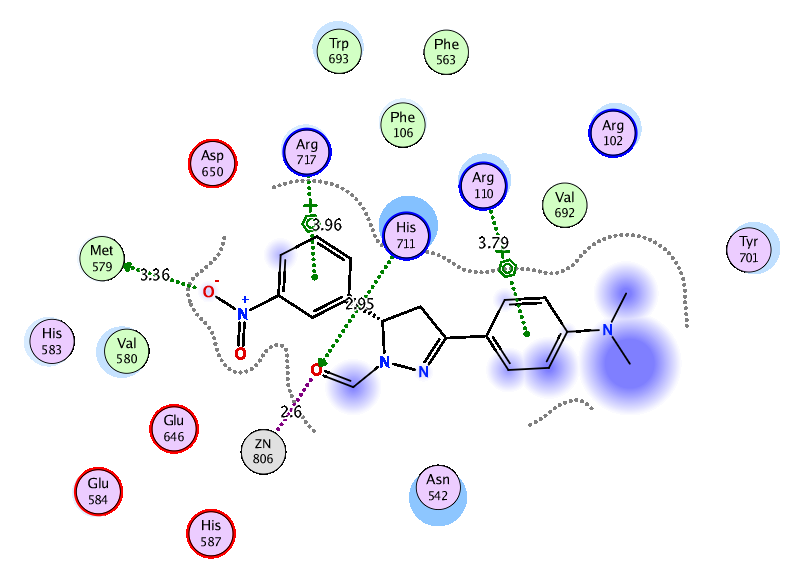 |
| **6** | **C97** | −6.2998 | 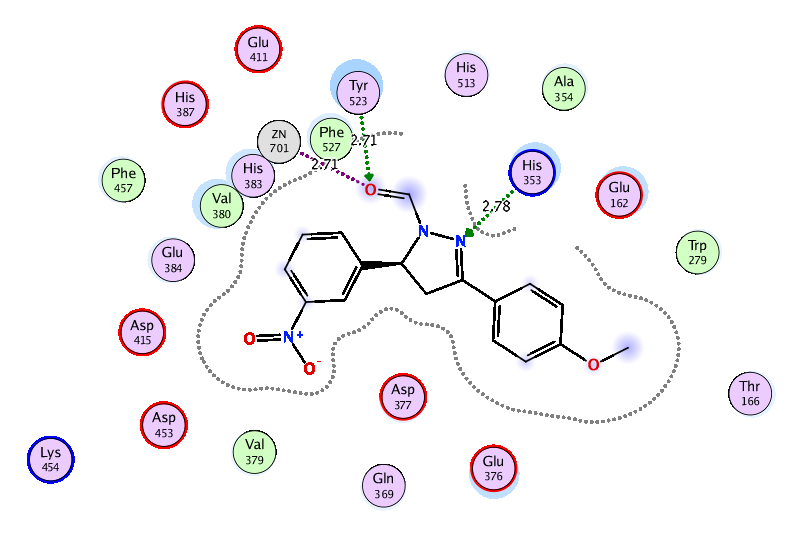 | −6.2006 | 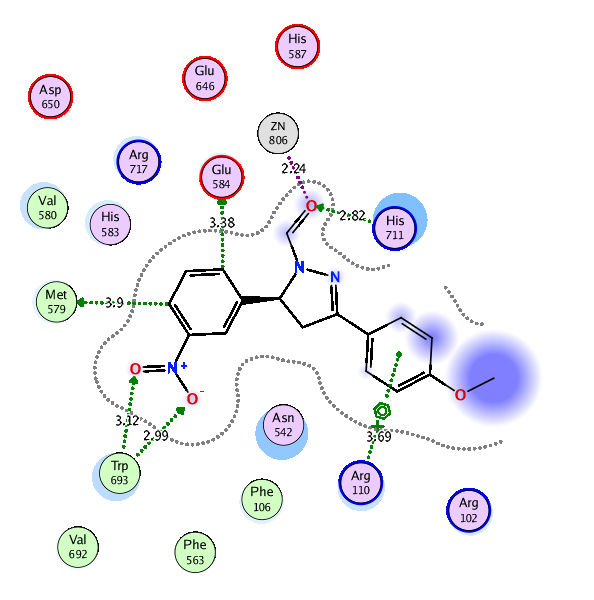 |
| **7** | **C98** | −5.8513 | 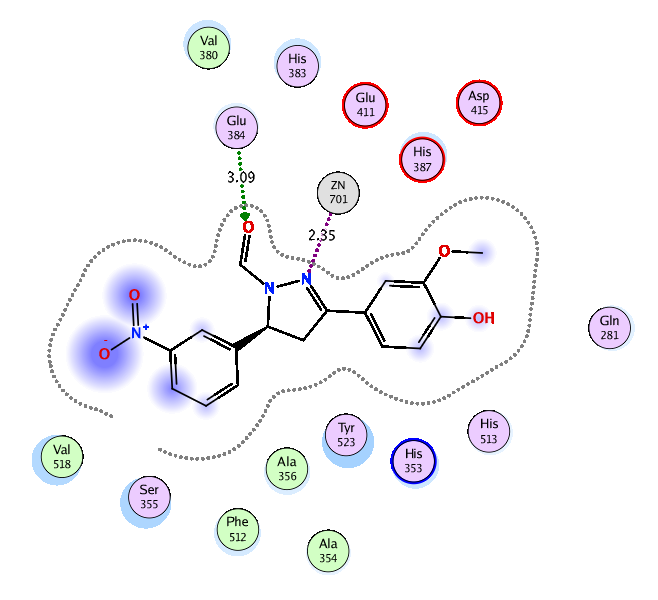 | −7.1859 | 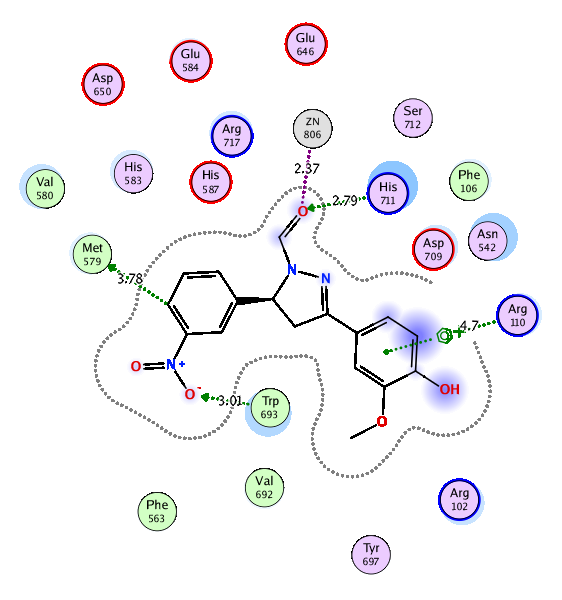 |
| **8** | **C99** | −5.7909 | 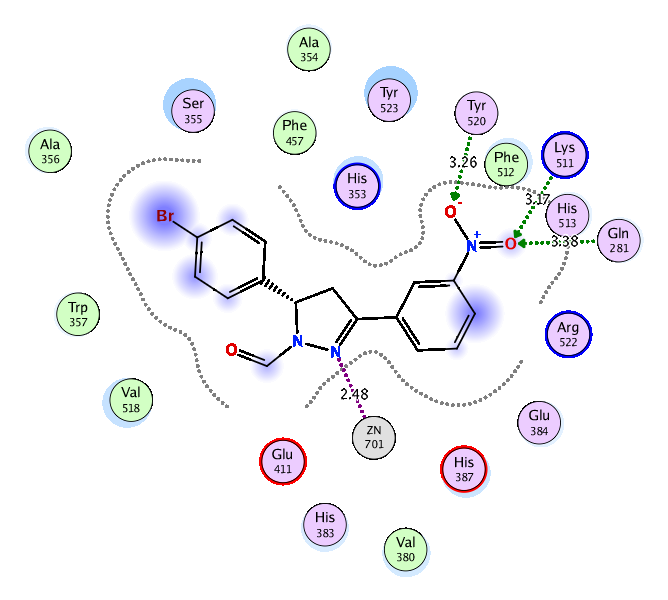 | −6.7488 | 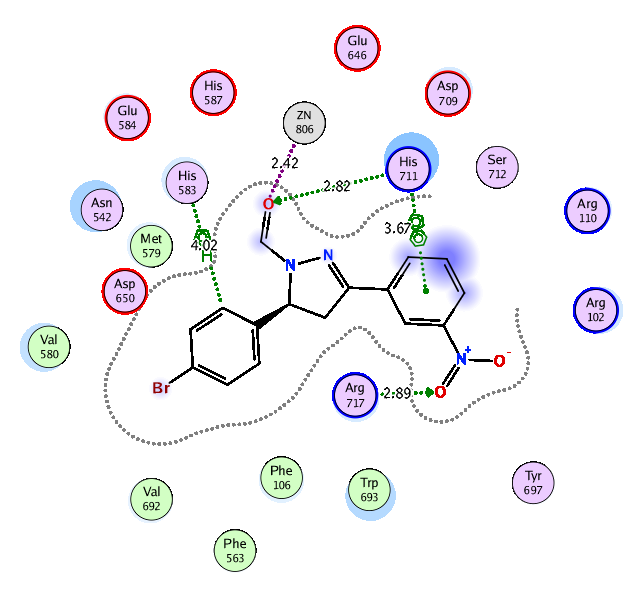 |
| **9** | **C101** | −5.8123 | 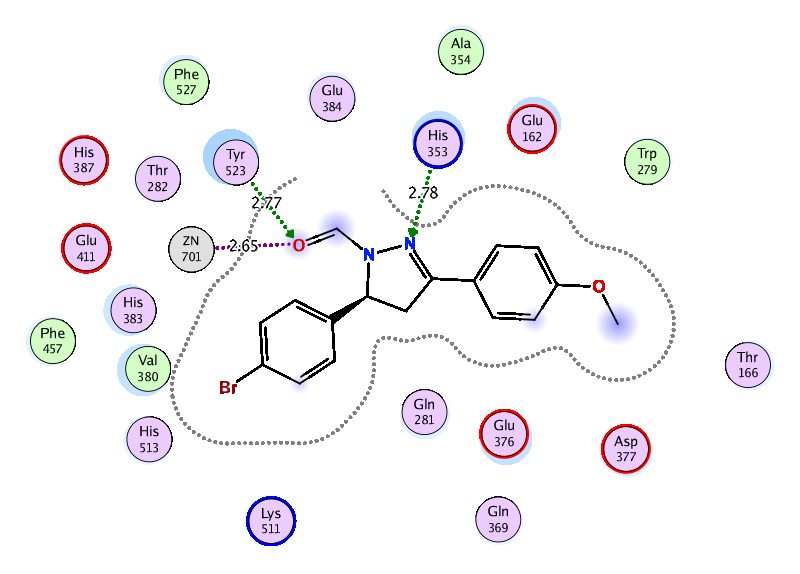 | −6.2563 | 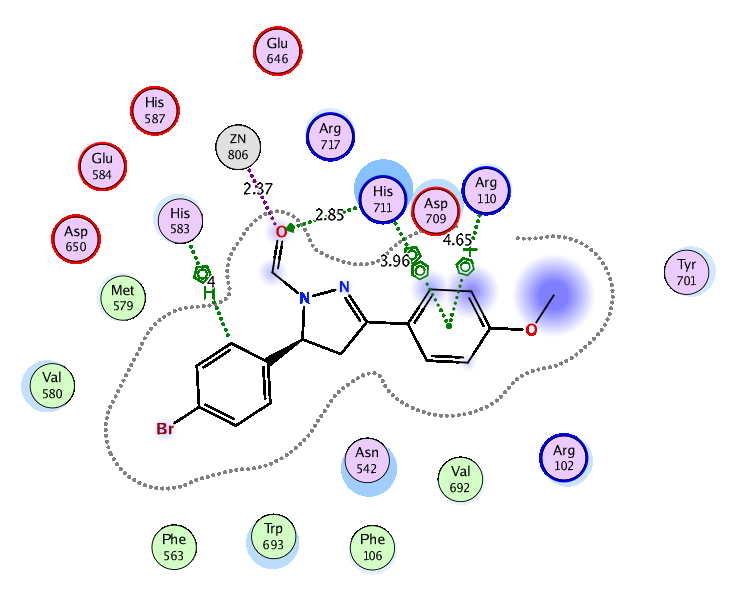 |
| **10** | **C102** | −5.9095 | 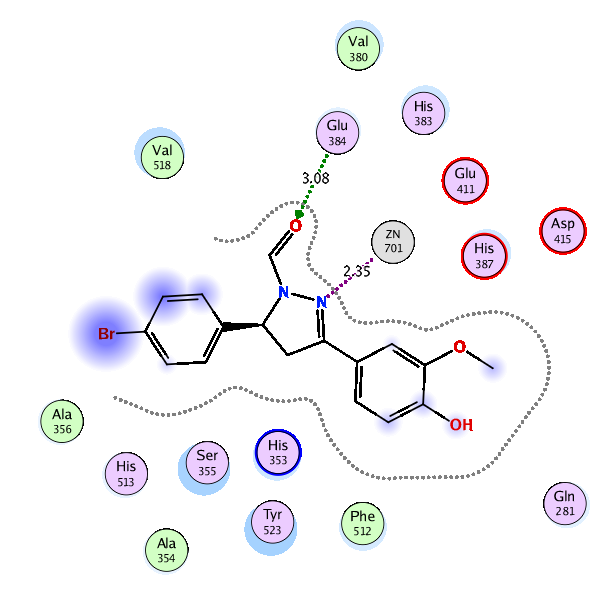 | −6.8404 | 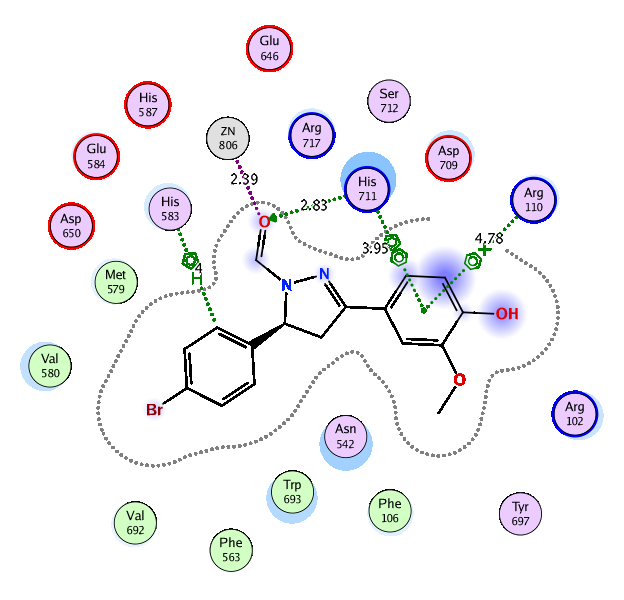 |
| **11** | **C105** | −5.6880 | 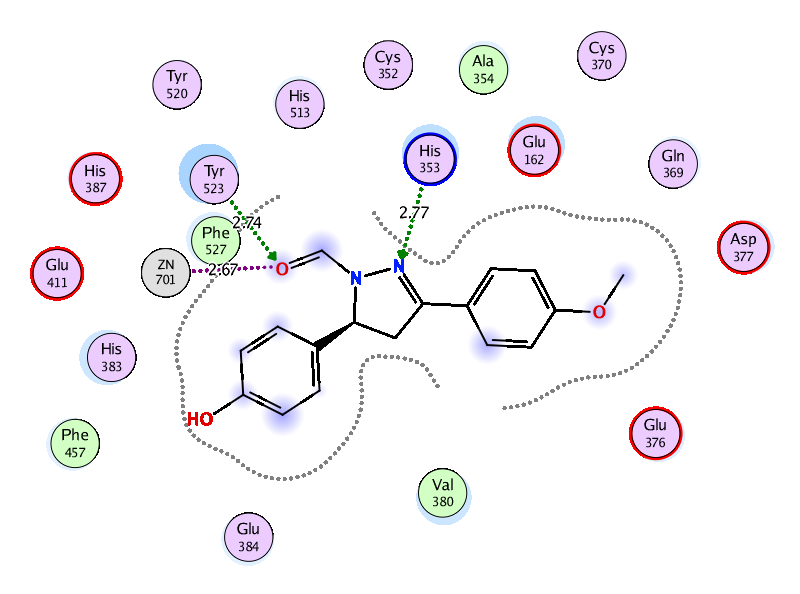 | −6.0413 | 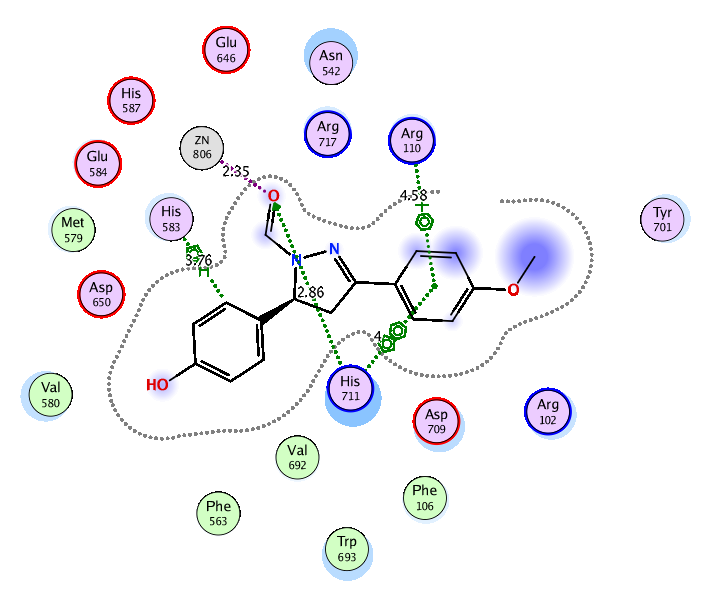 |
| **12** | **C115** | −5.7009 | 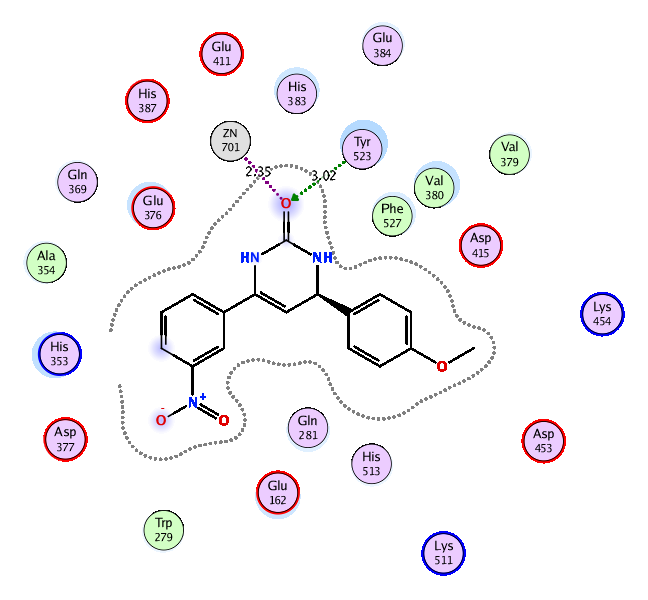 | −7.1057 | 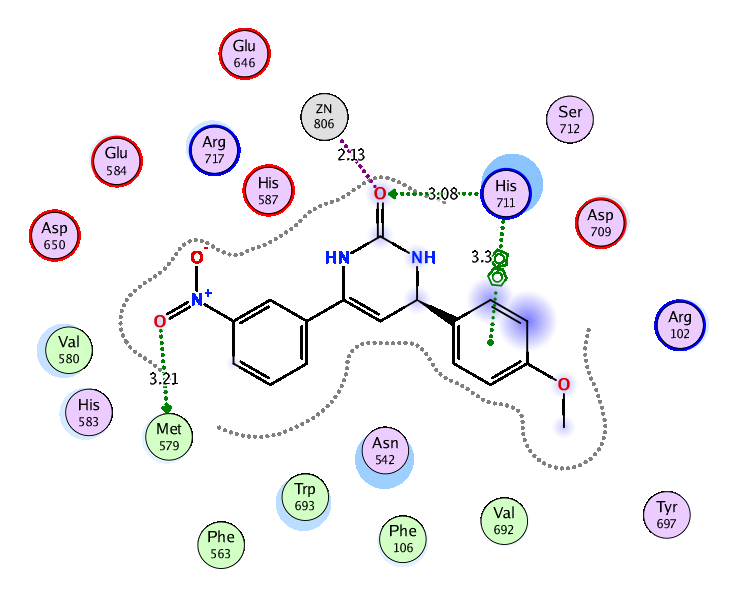 |
| **13** | **C145** | −6.0280 | 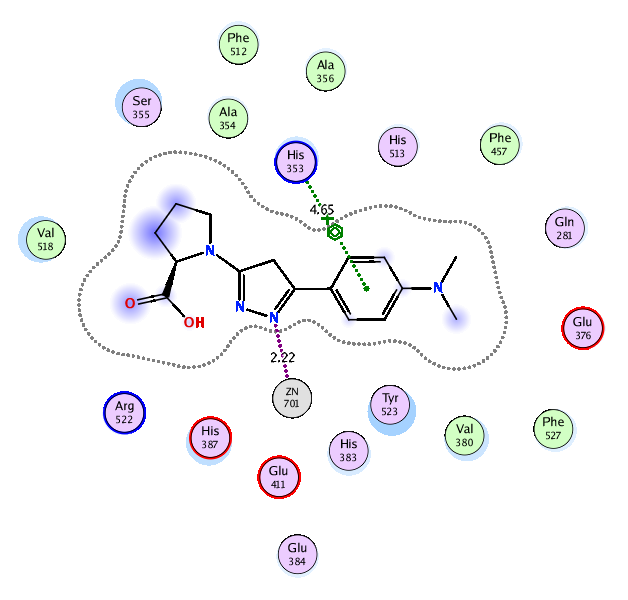 | −6.7257 | 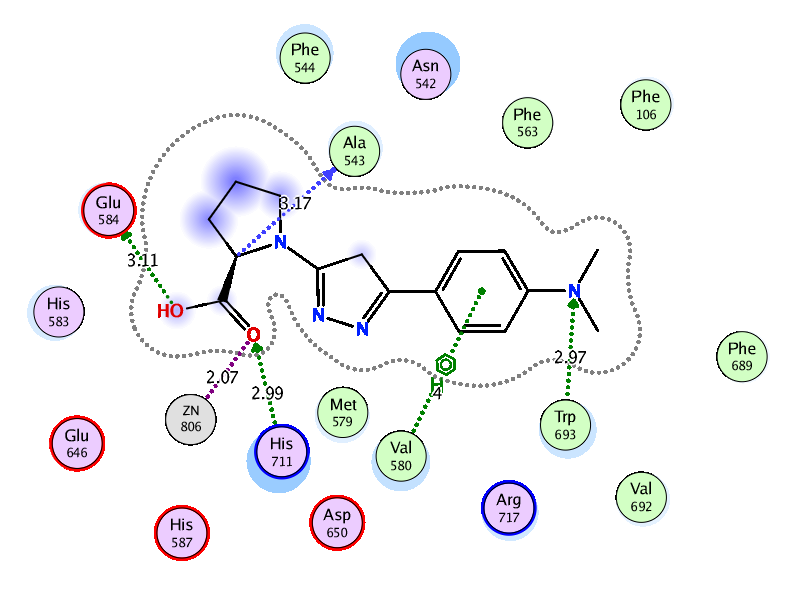 |
| **14** | **C146** | −5.5313 | 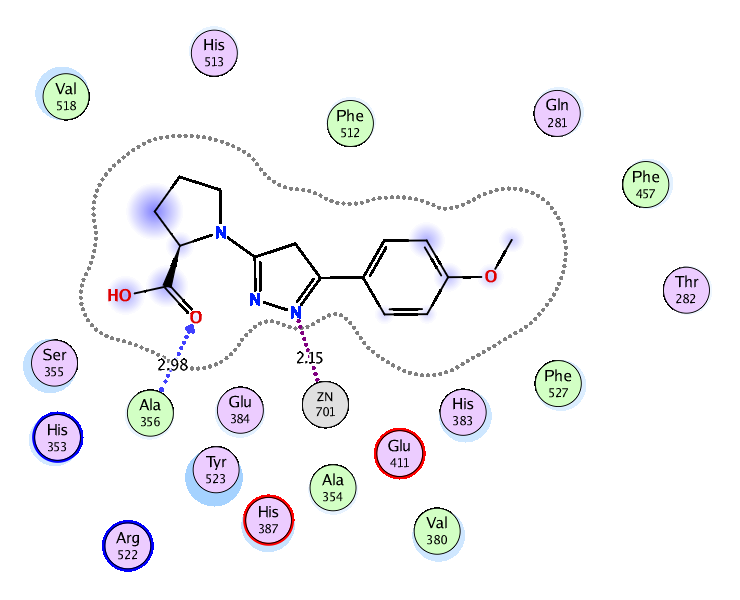 | −5.8255 | 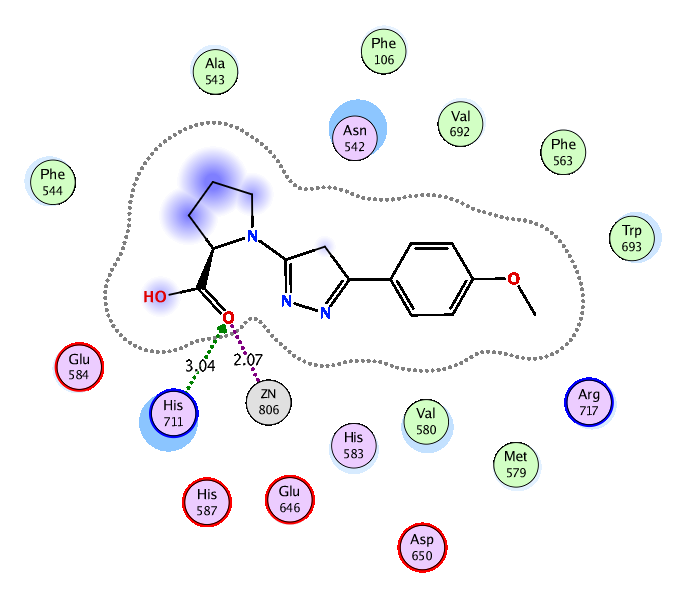 |
| **15** | **C148** | −5.6612 | 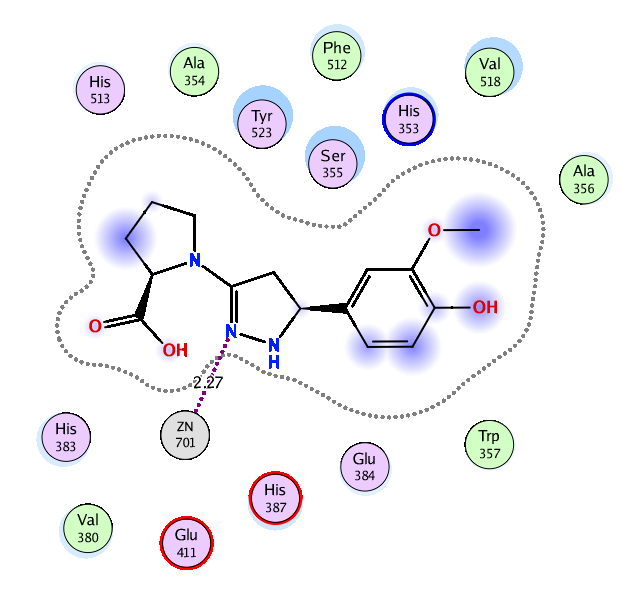 | −7.1215 | 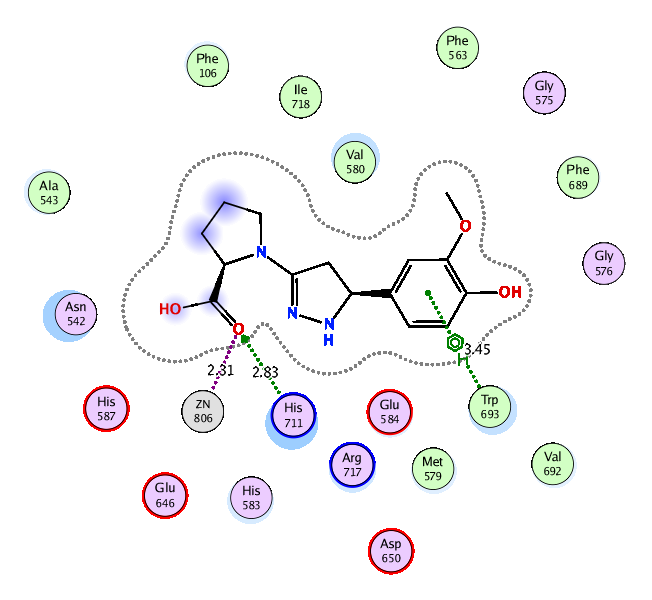 |
| **16** | **C164** | −6.3250 | 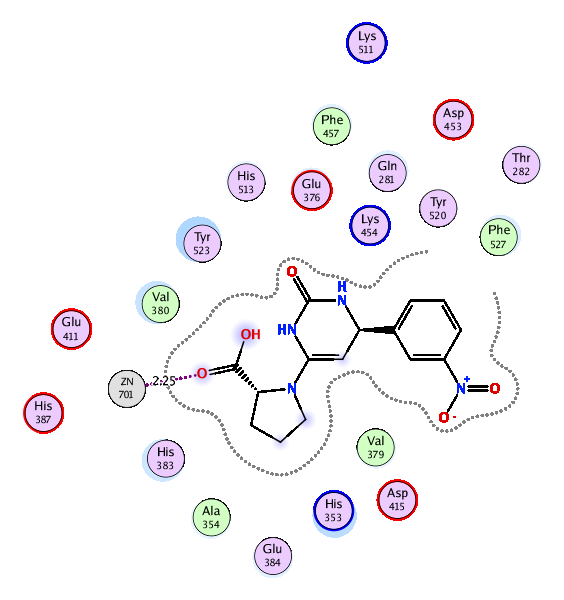 | −6.4206 | 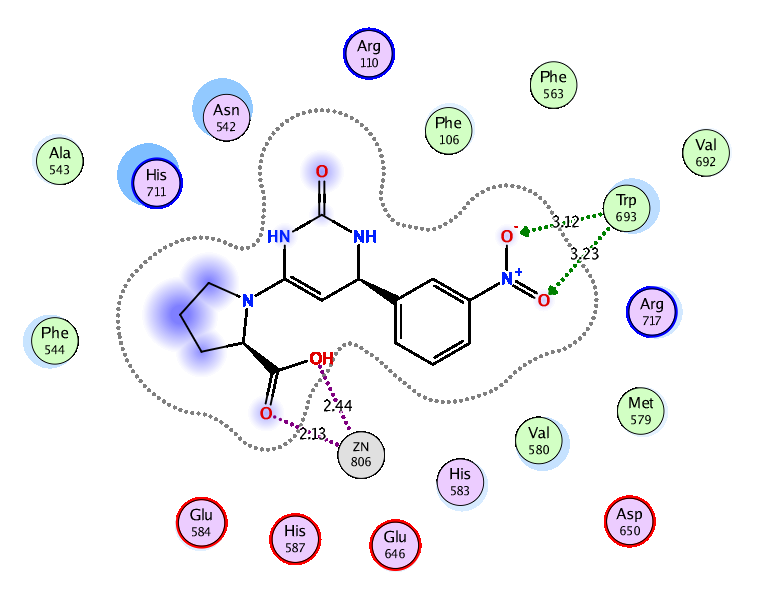 |
| **17** | **C165** | −5.8290 | 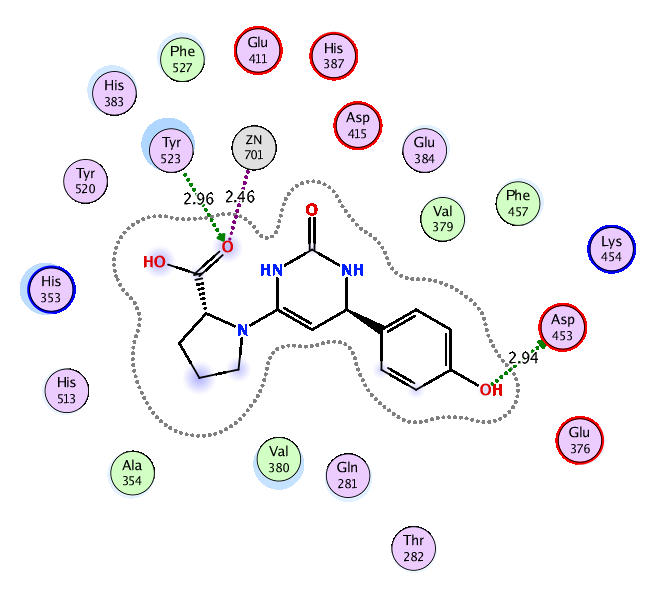 | −5.9617 | 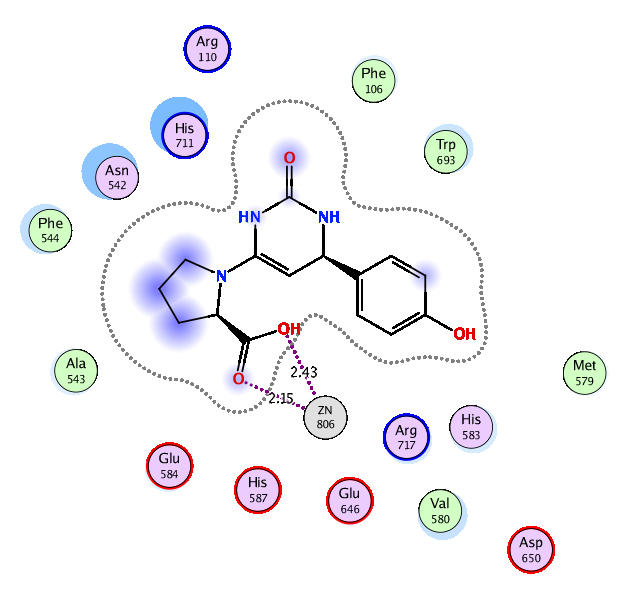 |
| **18** | **C167** | −6.0243 | 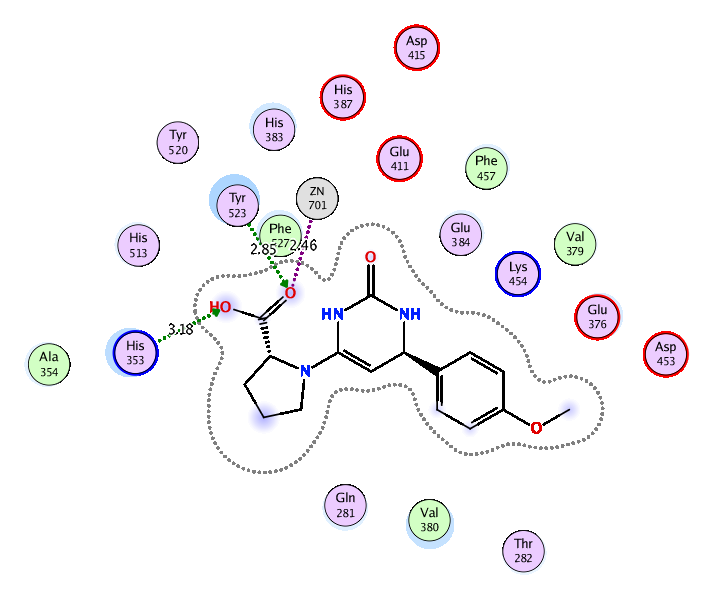 | −6.2916 | 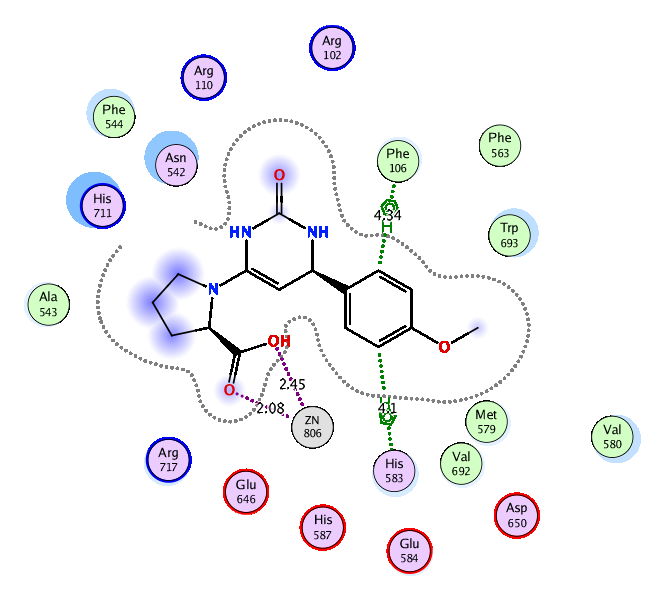 |
| **19** | **C169** | −5.5621 | 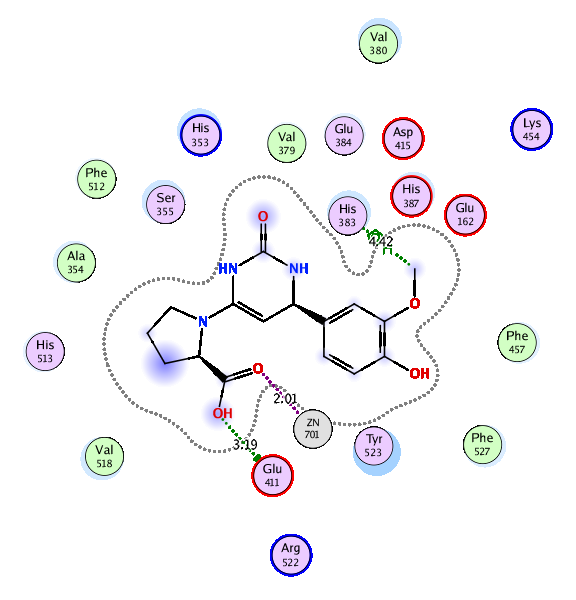 | −6.4222 | 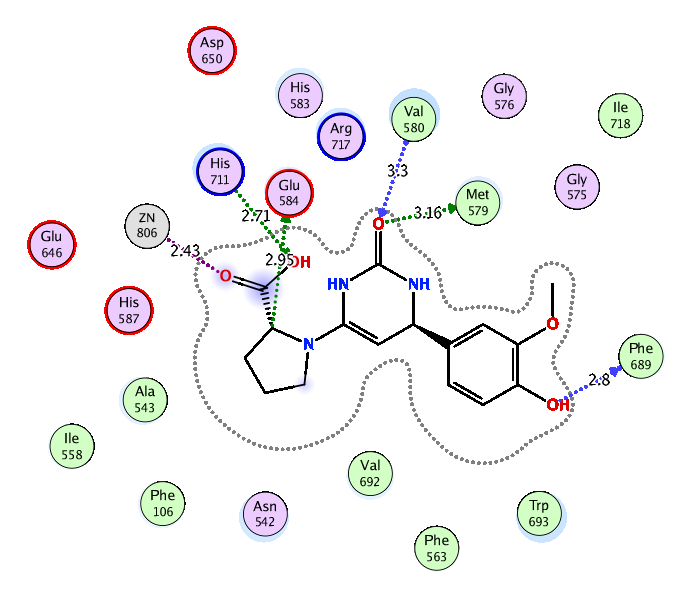 |
| **20** | **C191** | −5.8248 | 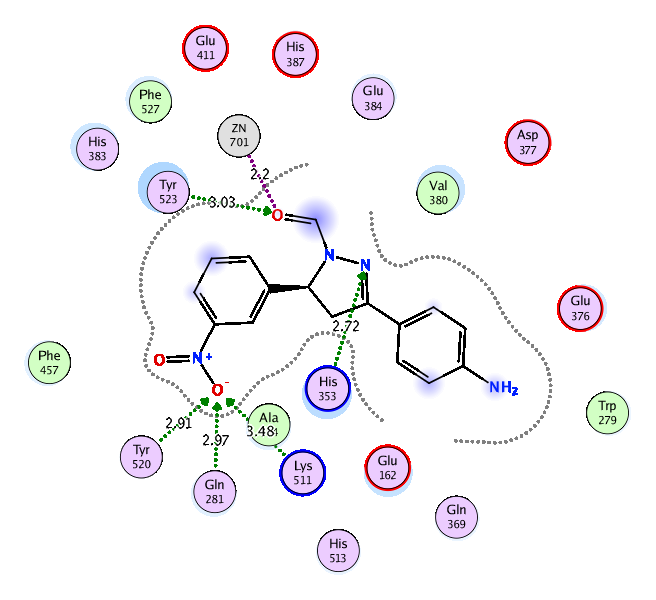 | −6.1590 | 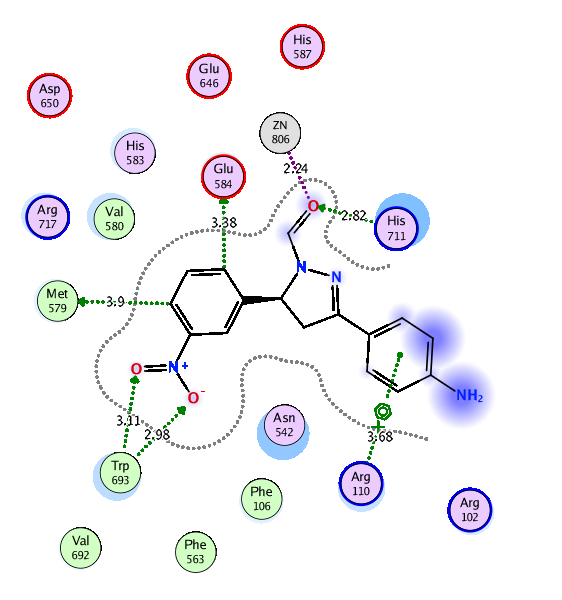 |
| **21** | **C229** | −6.4089 | 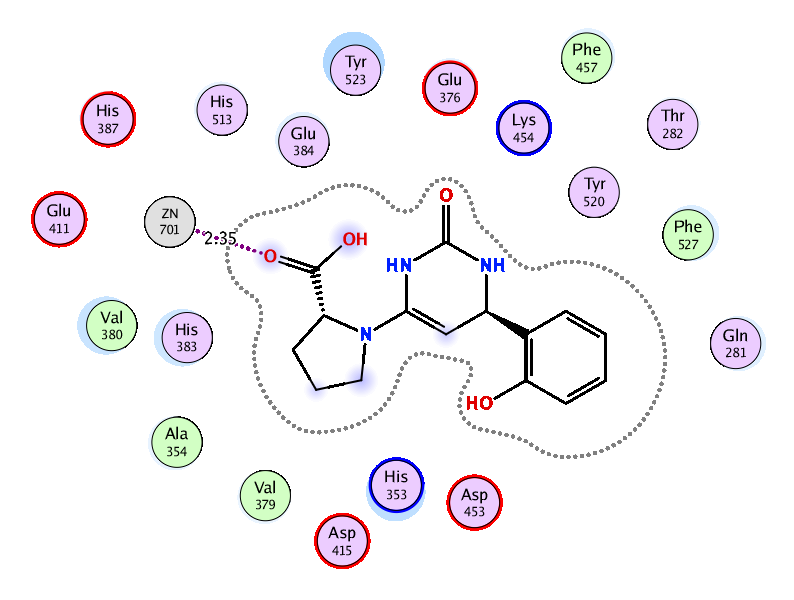 | −6.5466 | 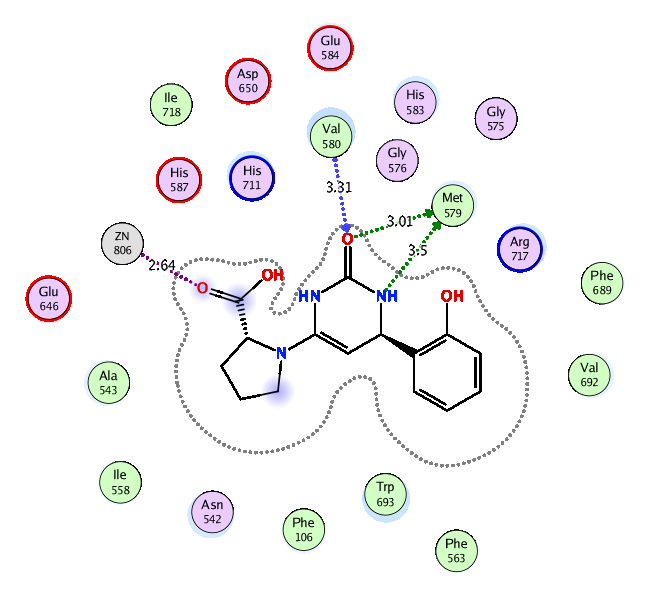 |

**Table S2: Molecular docking results of selected screened designed 1,3−Thiazole derivatives against cACE (PDB ID: 1O86) and NEP (PDB ID: 5JMY) enzyme**

| **Sr. No.** | **ID** | **Docking score against cACE** | **2D Interaction Diagram of ligands with cACE enzyme** | **Docking score against NEP** | **2D Interaction Diagram of ligands with NEP enzyme** |
| --- | --- | --- | --- | --- | --- |
|  | **T1** | −7.7185 | 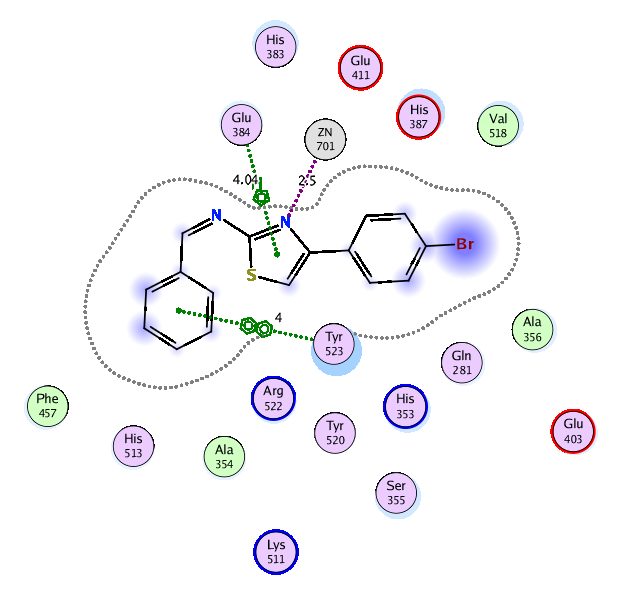 | −6.5466 | 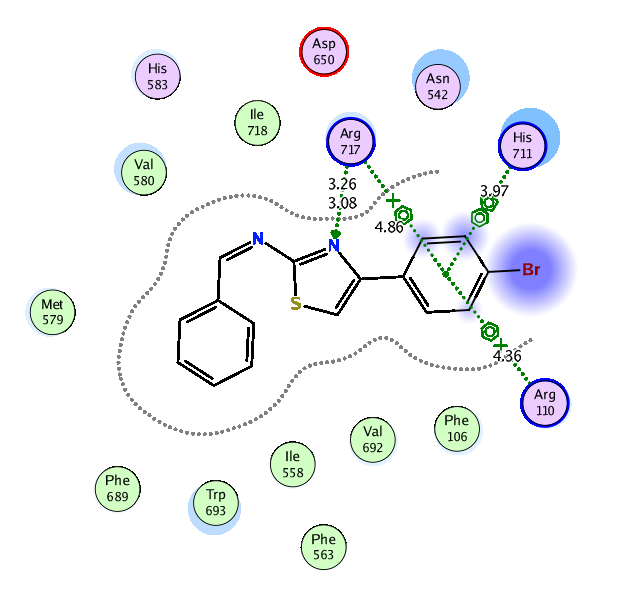 |
|  | **T3** | −6.9584 | 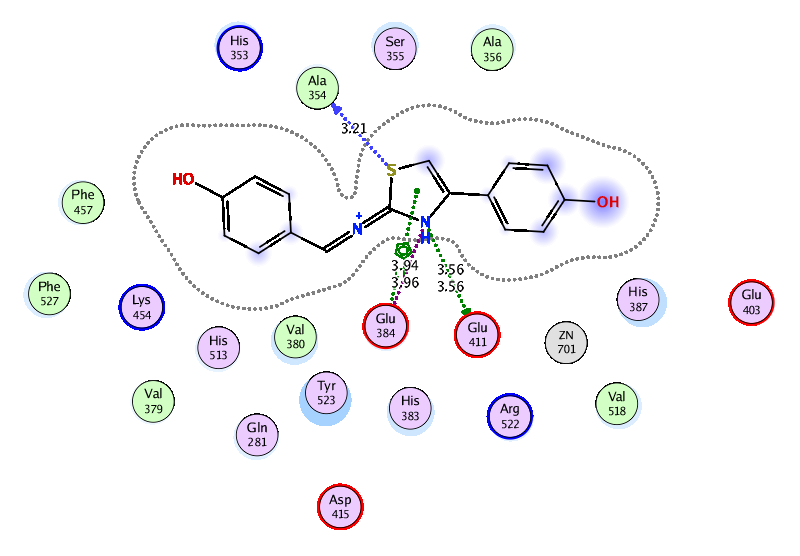 | −7.1859 | 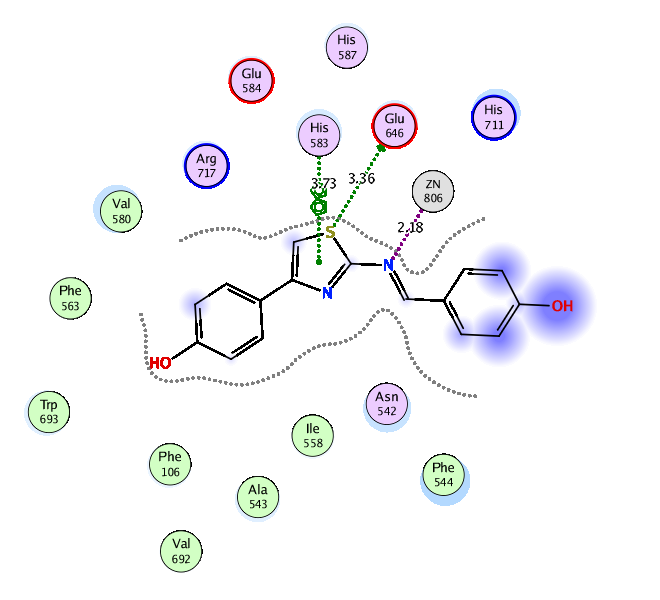 |
|  | **T9** | −4.7256 | 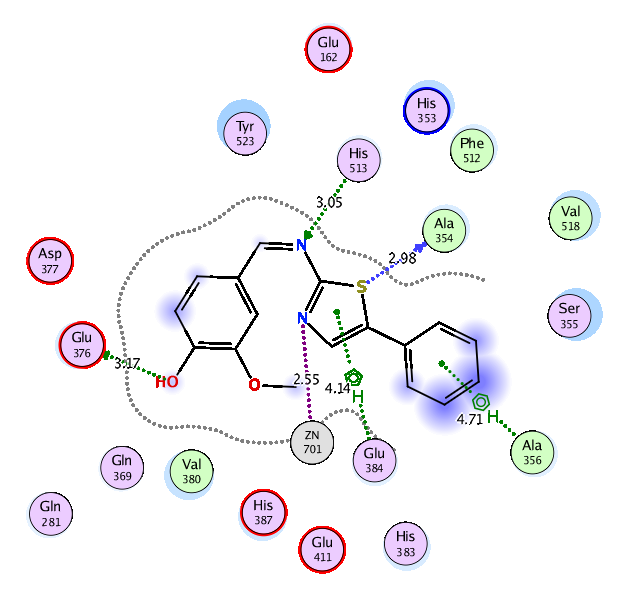 | −5.1339 | 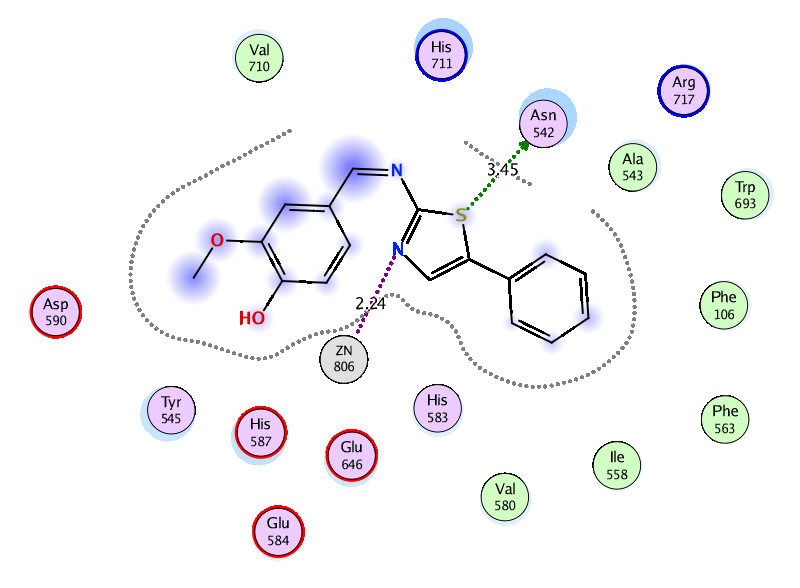 |
|  | **T10** | −4.9357 | 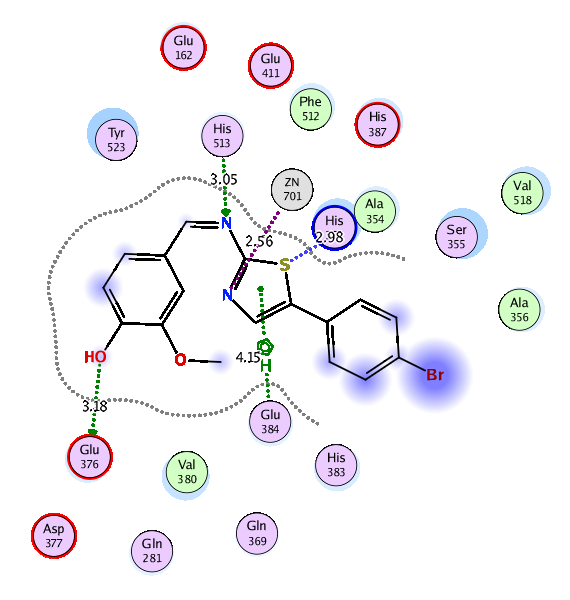 | No pose |  |
|  | **T11** | −5.6430 | 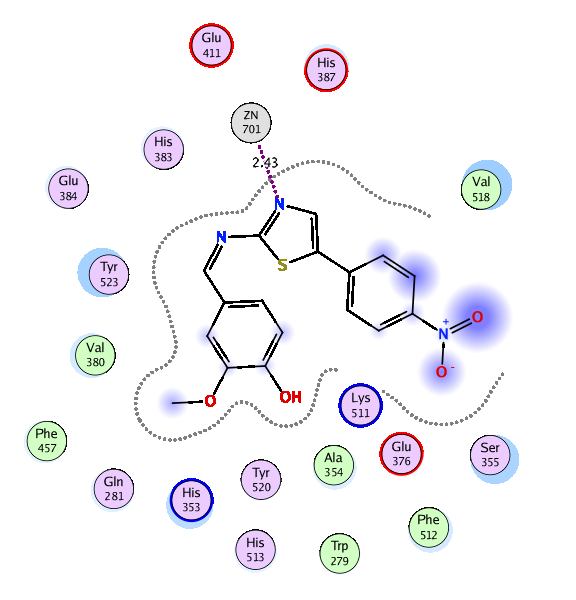 | −5.8640 | 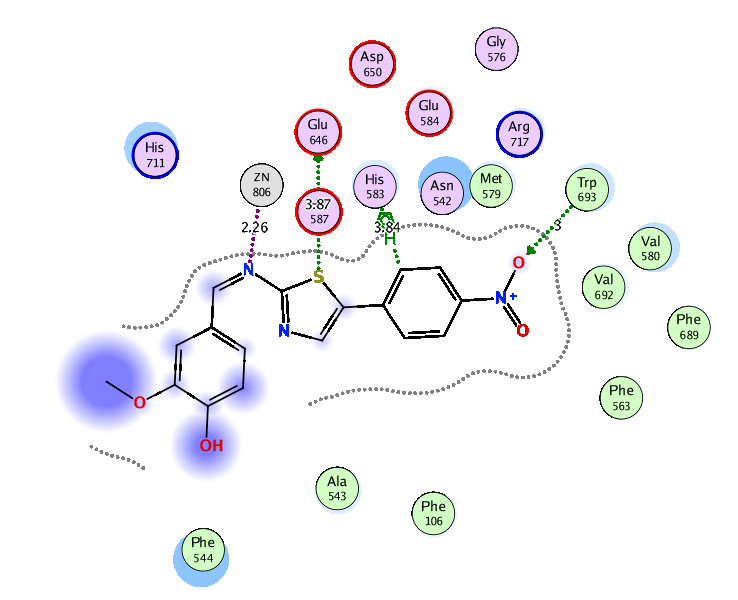 |
|  | **T16** | No pose |  | −5.1430 | 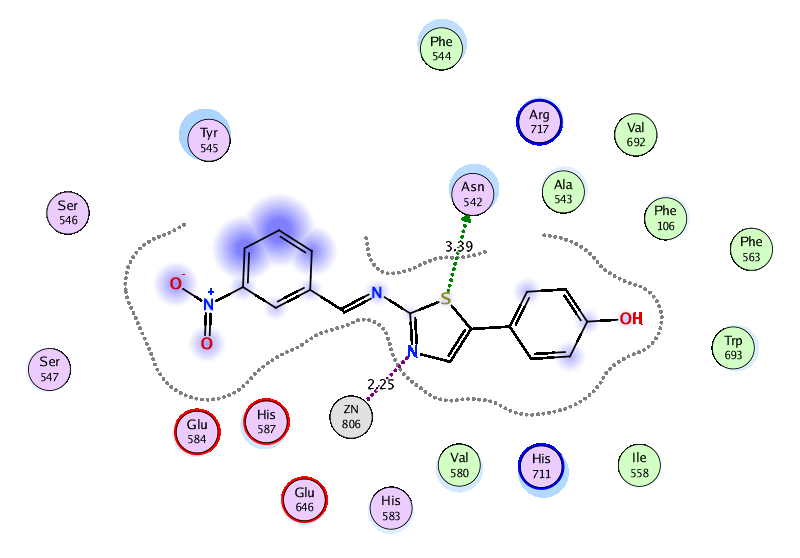 |
|  | **T17** | −4.2001 | 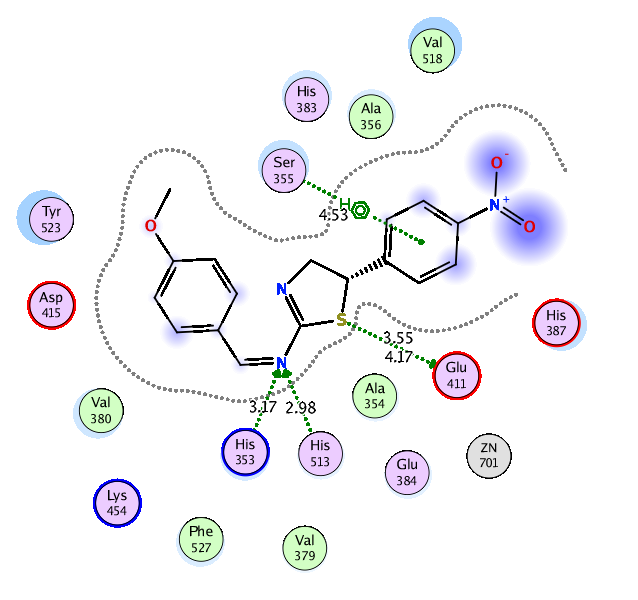 | −4.6230 | 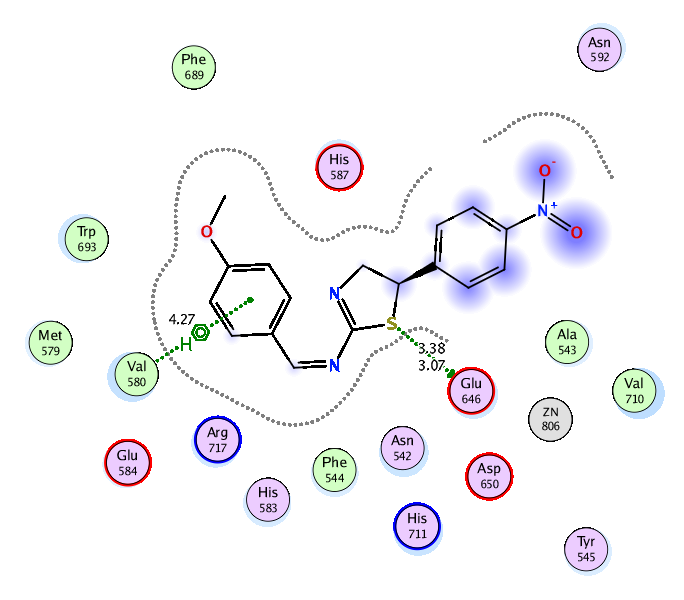 |
|  | **T20** | No pose |  | −4.7410 | 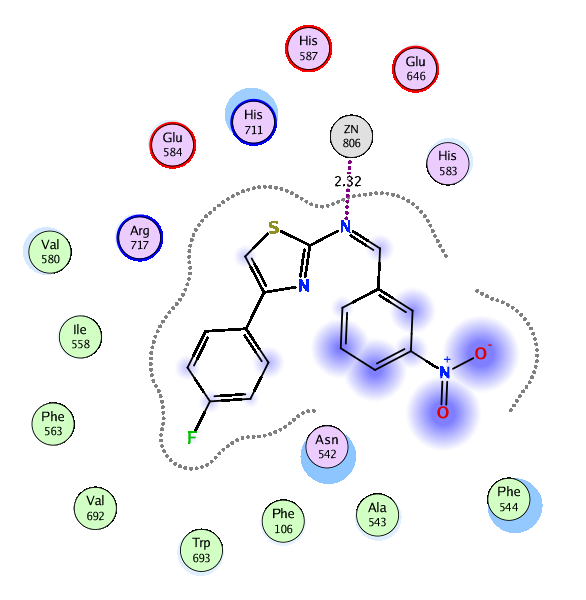 |
|  | | | | | |

**Table S3: Molecular docking results of selected screened designed 1,3,4−Thiadiazole derivatives against cACE (PDB ID: 1O86) and NEP (PDB ID: 5JMY) enzyme**

| **Sr. No.** | **ID** | **Docking score against cACE** | **2D Interaction Diagram of ligands with cACE enzyme** | **Docking score against NEP** | **2D Interaction Diagram of ligands with NEP enzyme** |
| --- | --- | --- | --- | --- | --- |
|  | **TD6** | −5.5672 | 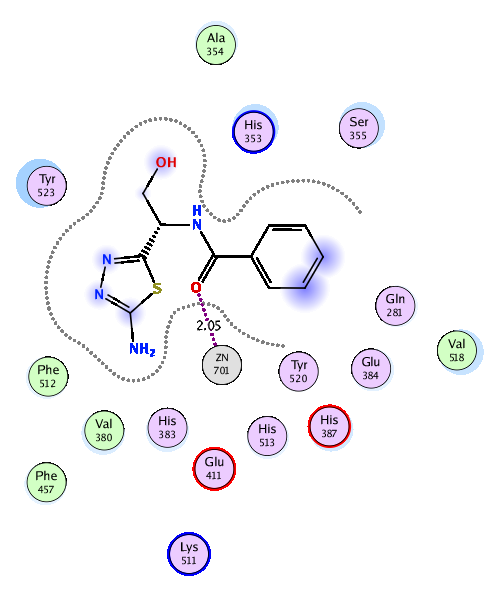 | −5.1171 | 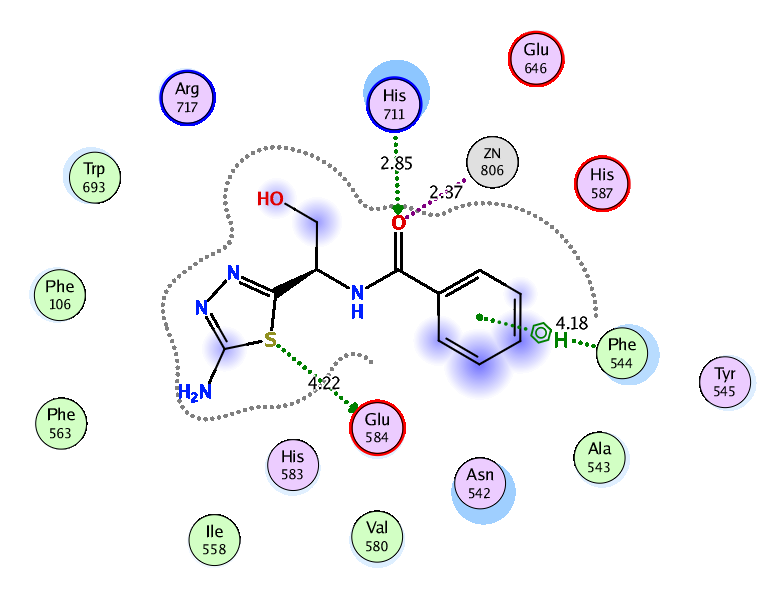 |
|  | **TD7** | −5.4932 | 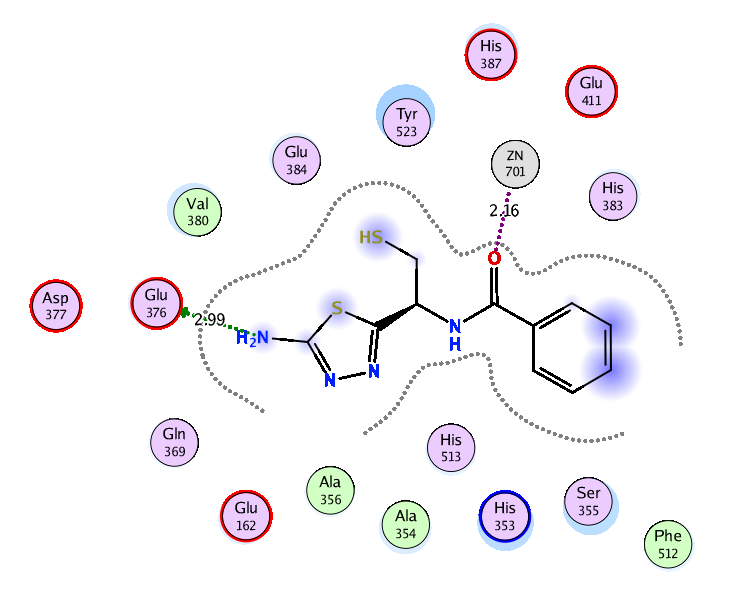 | −5.2422 | 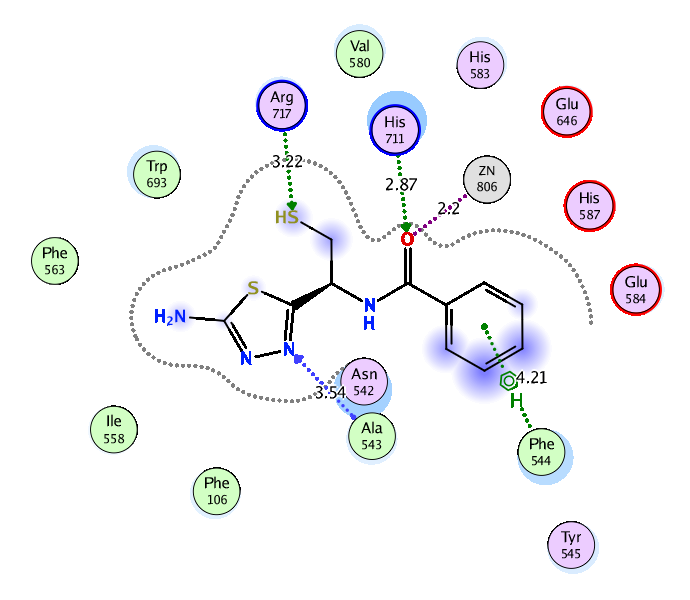 |
|  | **TD33** | −6.3711 | 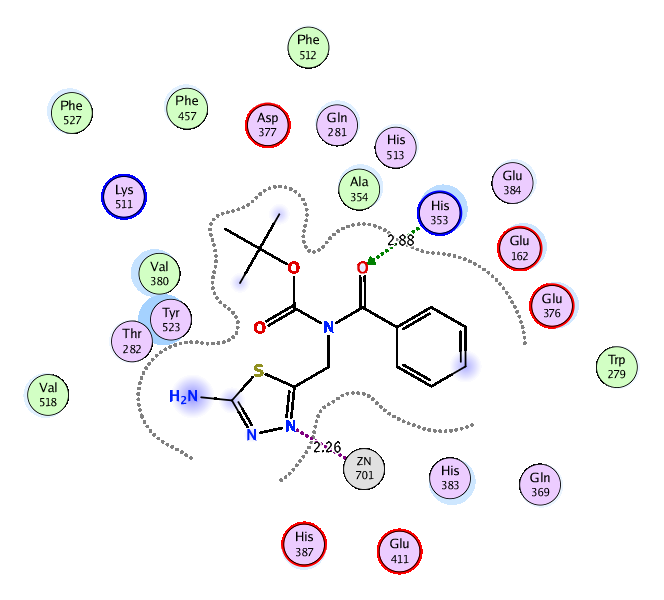 | −5.8966 | 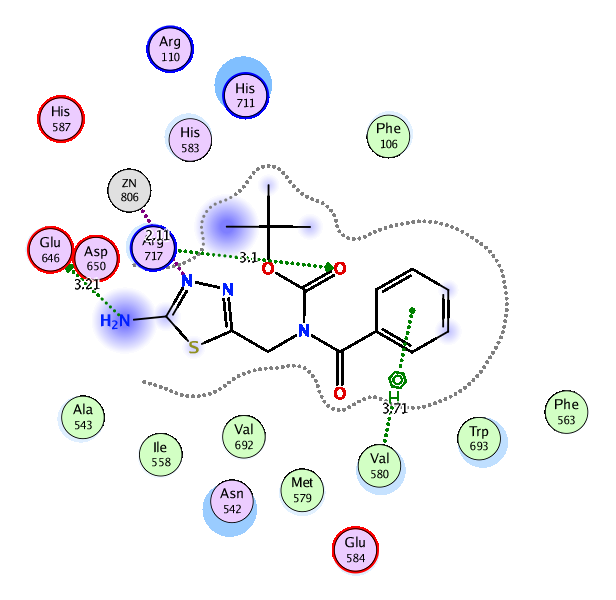 |
|  | **TD64** | −6.1358 | 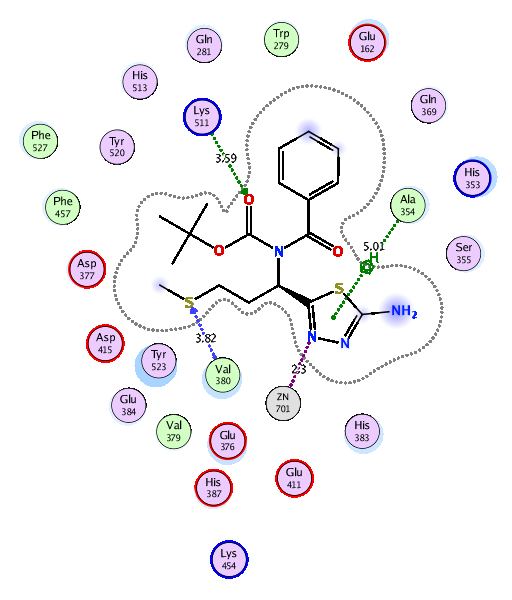 | −6.1683 | 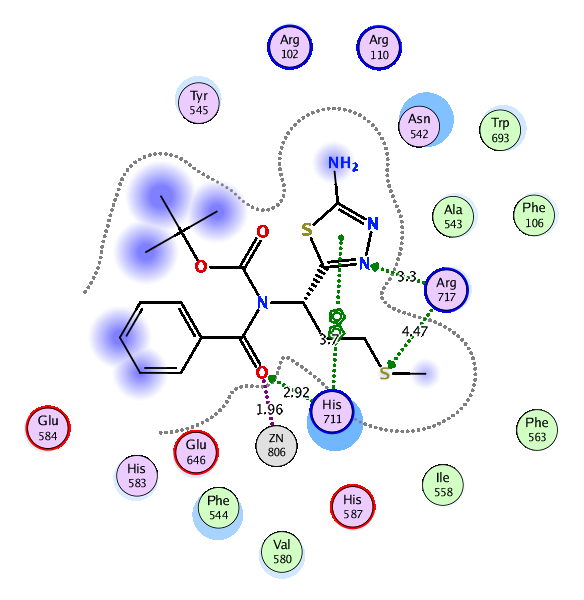 |
|  | **TD75** | −7.6001 | 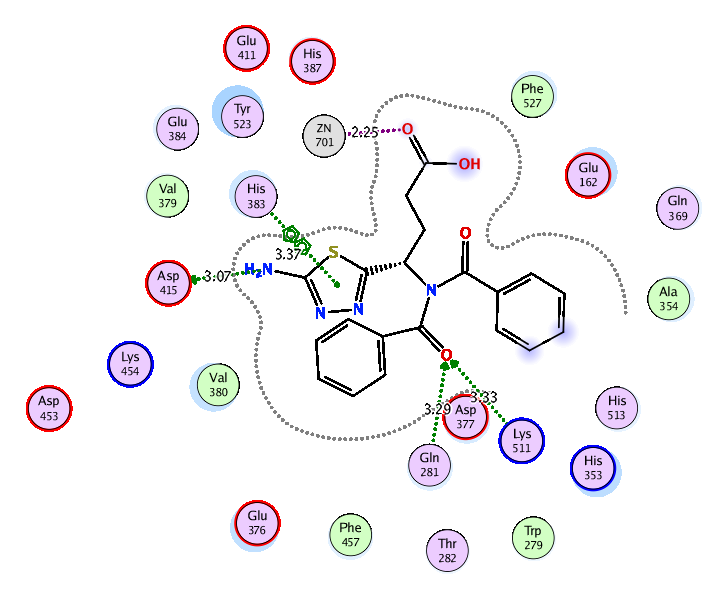 | −5.3231 | 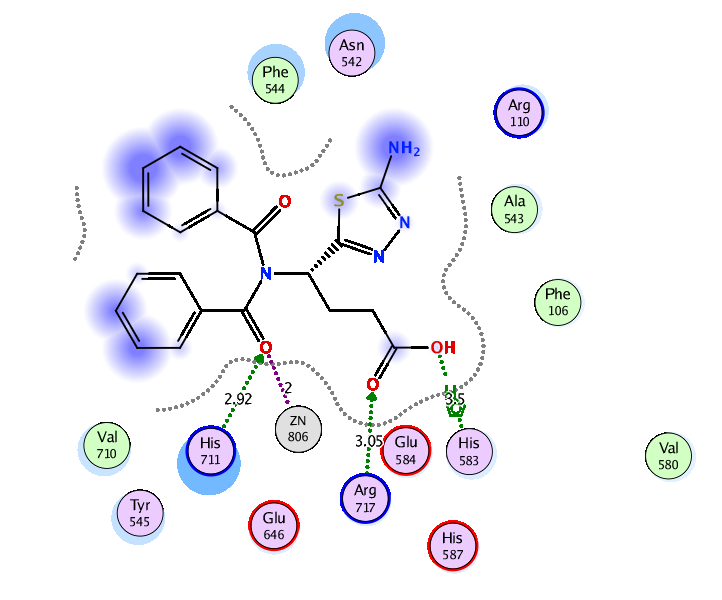 |
|  | **TD98** | −9.4353 | 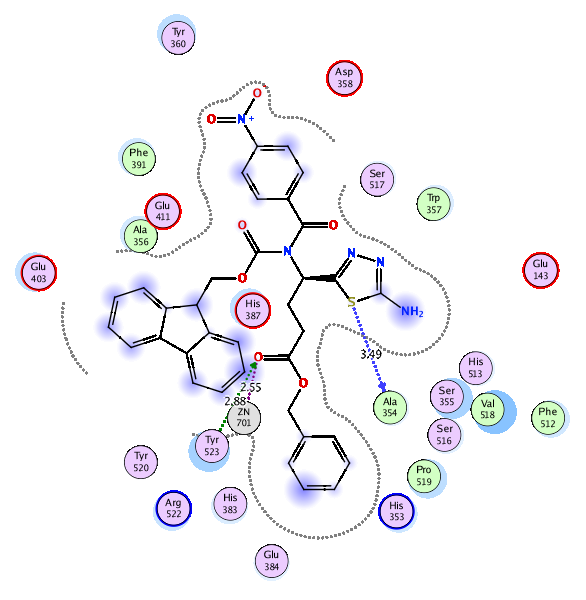 | −8.7272 | 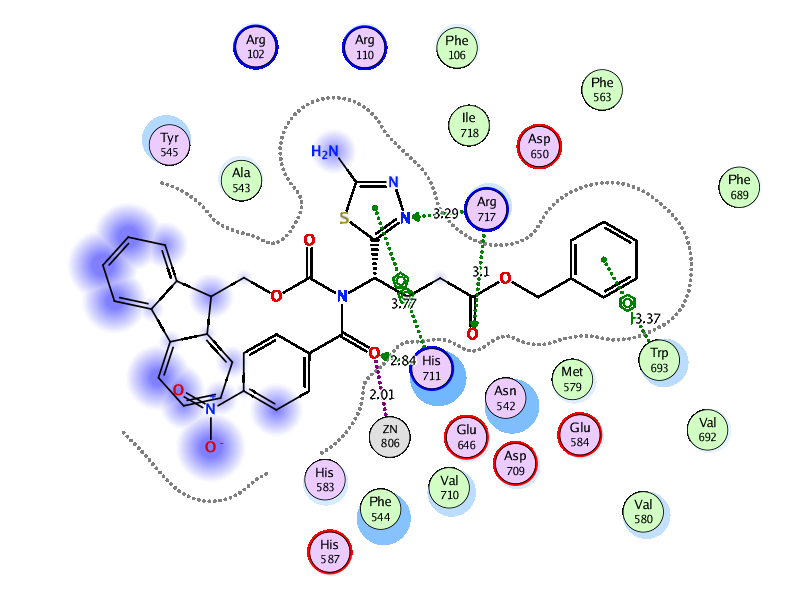 |
|  | **TD101** | −0.0854 | 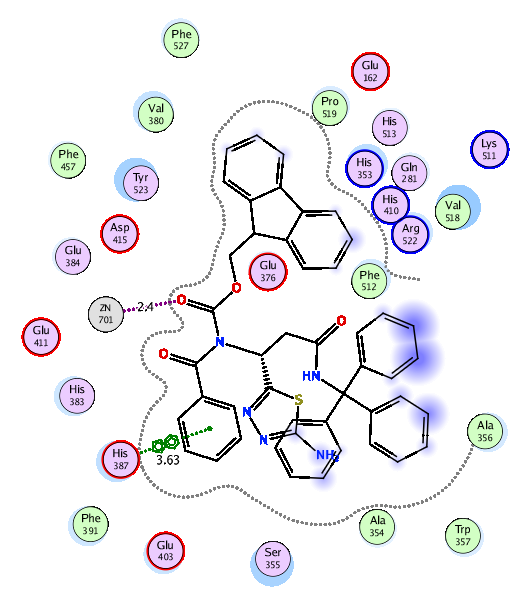 | −7.4954 | 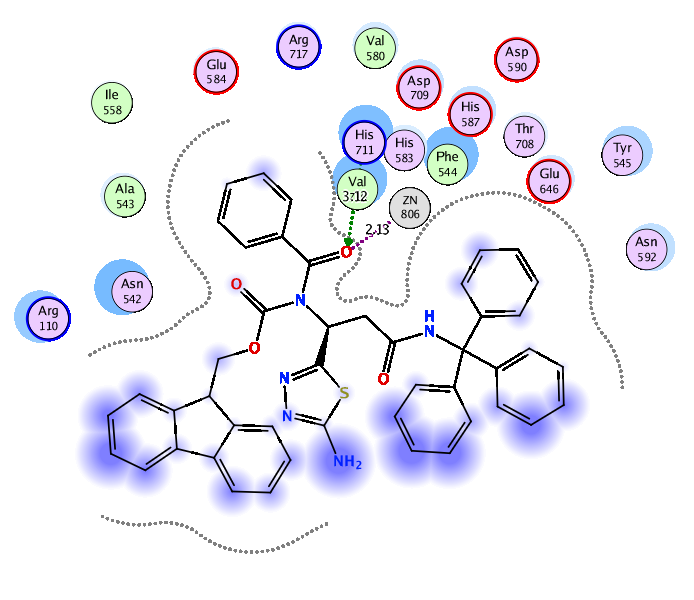 |
|  | **TD103** | −5.2566 | 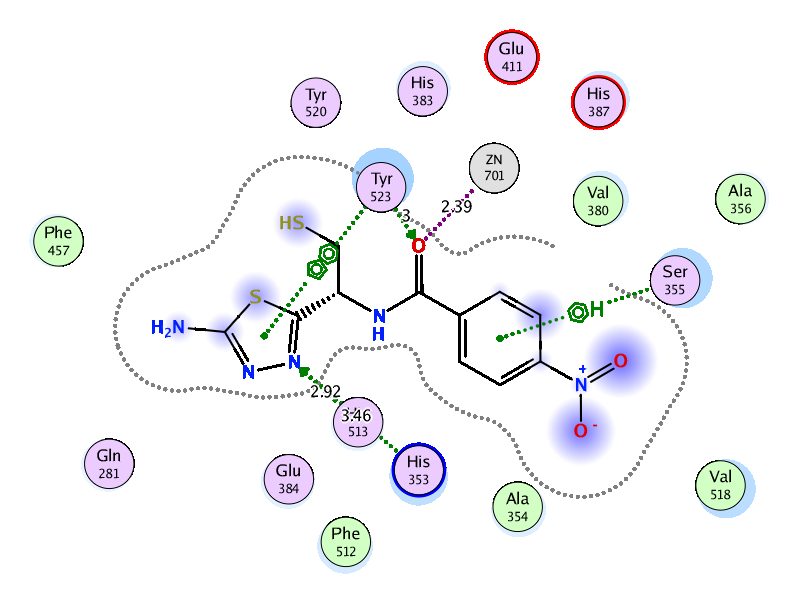 | No pose |  |
|  | **TD104** | −8.1032 | 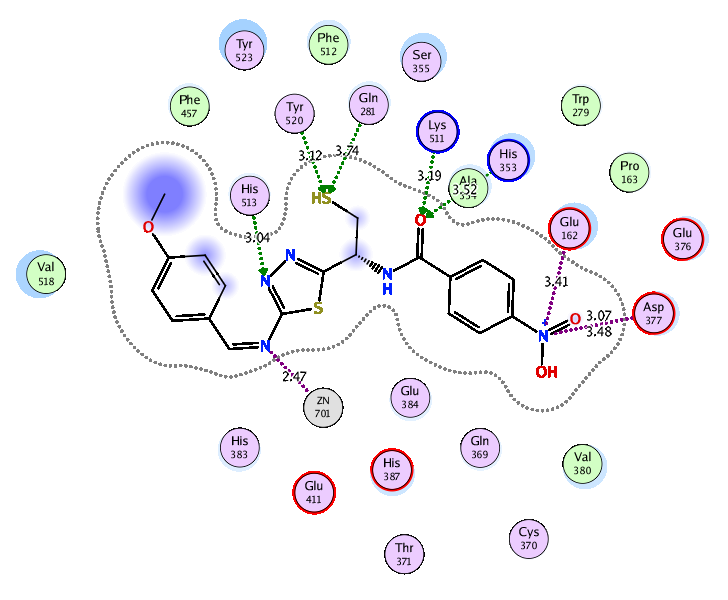 | −7.5842 | 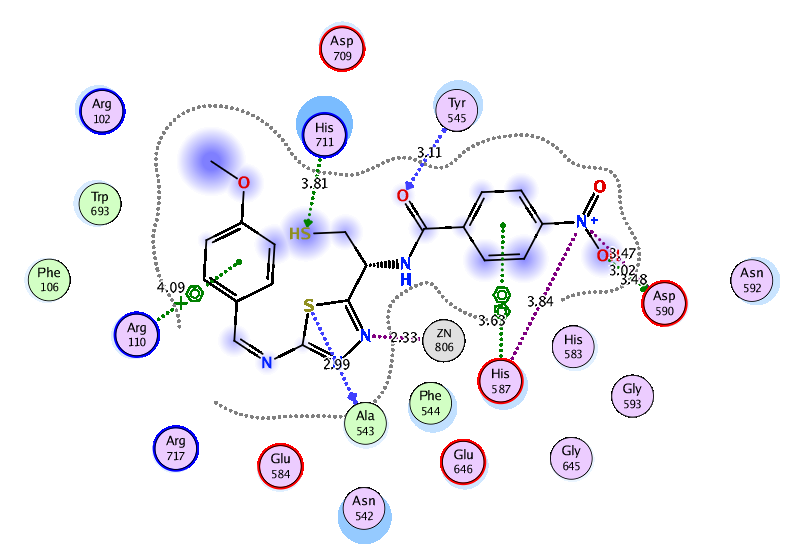 |
|  | **TD105** | −5.1424 | 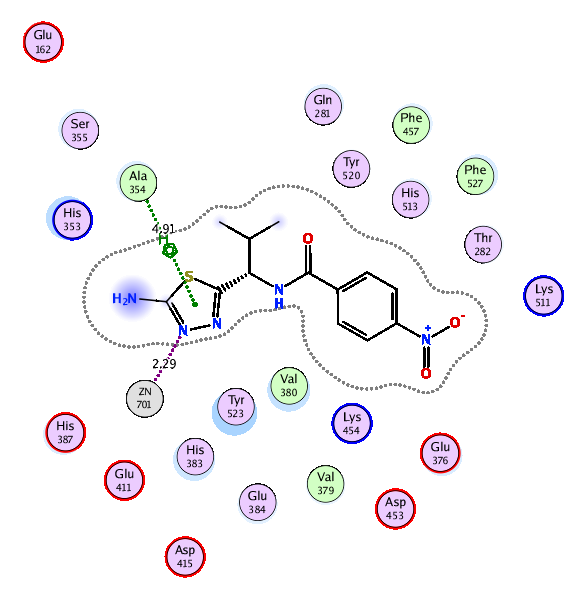 | −8.3799 | 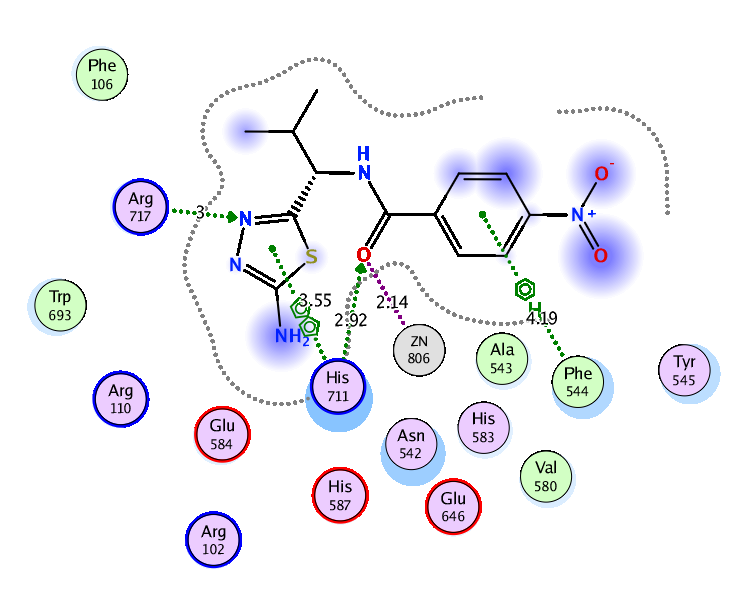 |
|  | **TD106** | −5.7235 | 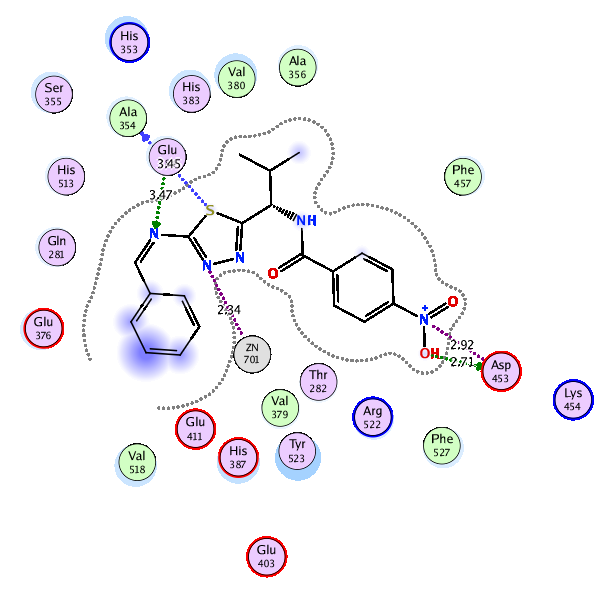 | −7.7640 | 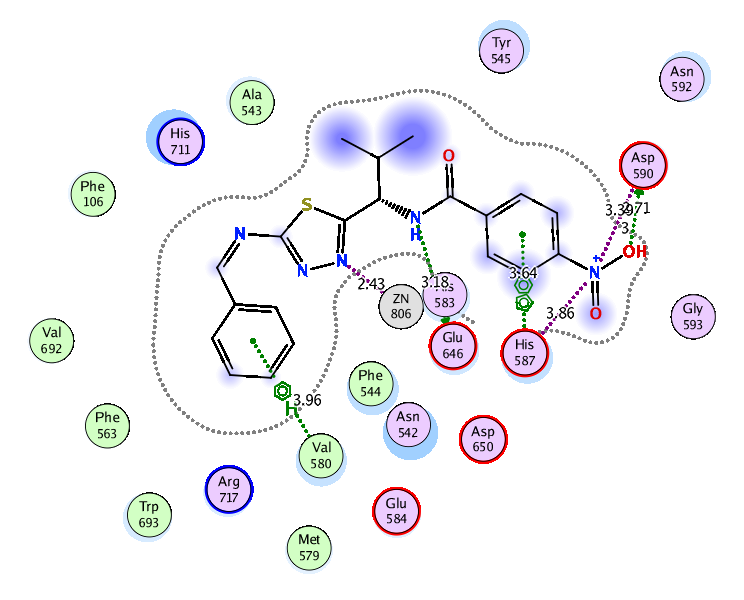 |
