# Supplementary material for: Multi-Target In-Silico modeling strategies to discover novel angiotensin converting enzyme and neprilysin dual inhibitors
Source: Sci Rep. 2024 Jul 10;14:15991. doi: 10.1038/s41598-024-66230-7 (PMC11237057; doi:10.1038/s41598-024-66230-7)
Supplement: Supplementary file 10 — Supplementary Information 8. [file 41598_2024_66230_MOESM10_ESM.docx]

Manuscript type: Full Paper

Submission to: Scientific Reports

Multi-Target *In-Silico* Modeling Strategies to Discover Novel Angiotensin Converting Enzyme and Neprilysin Dual Inhibitors

Sapan Shah[a], Dinesh Chaple[a], Vijay H. Masand[b], Rahul Jawarkar[c], Somdatta Chaudhari[d], A. Abiramsundari [e] Magdi E.A. Zaki [f], Sami A. Al-Hussain[f]

[a]Department of Pharmaceutical Chemistry, Priyadarshini J. L. College of Pharmacy, Hingna Road, Nagpur-440016, Maharashtra, India.

[b]Department of Chemistry, Vidya Bharati Mahavidyalaya, Amravati-444601, Maharashtra, India. Orcid ID: 0000-0001-9300-4147

[c]Department of Medicinal Chemistry and Drug Discovery, Dr. Rajendra Gode Institute of Pharmacy, University Mardi Road, Amravati 444603, India; Email: rahuljawarkar@gmail.com. Orcid ID: 0000-0003-3563-6642

[d] Department of Pharmaceutical Chemistry, Modern College of Pharmacy, Nigdi, Pune

[e]Biobay, Ahmedabad, India

[f]Department of Chemistry, Faculty of Science, Imam Mohammad Ibn Saud Islamic University, Riyadh 13318, Saudi Arabia.

***Supporting File S1:*** ACE and NEP enzymes inhibitors dataset collected from ChEMBL for development of mt-QSAR models

***Supporting File S2:*** Curated dataset containing 715 compounds (ACE-357 and NEP-358) and 1560 compounds (AT1-1091 and NEP-469)

***Supporting File S3:*** Details of the calculated PyDescriptors, Alvadesc and Padel for 715 compound dataset

***Supporting File S4:*** Modified descriptors prepared by using Box–Jenkins operators

***Supporting File S5:*** Details of the total designed chalcones and its derivatives (235 compounds), 1, 3- Thiazole (24 compounds) and 1,3,4-Thiadiazole (107 compounds)

***Supporting File S6:*** Details of this screened dataset of chalcone, 1,3-thiazole and 1,3,4-thiadiazole derivatives as well as the results of the predictions using mt-QSAR models

***Supporting File S7:*** Molecular docking results of selected screened designed chalcone derivatives (total 85 compounds) against cACE (PDB ID: 1O86) and NEP (PDB ID: 5JMY) enzyme
